# Synthesis, Molecular Docking Analysis and Biological Evaluations of Saccharide-Modified Thiadiazole Sulfonamide Derivatives

**DOI:** 10.3390/ijms22115482

**Published:** 2021-05-22

**Authors:** Zuo-Peng Zhang, Ye Zhong, Zhen-Bin Han, Lin Zhou, Hua-Sheng Su, Jian Wang, Yang Liu, Mao-Sheng Cheng

**Affiliations:** Key Laboratory of Structure-Based Drug Design & Discovery of Ministry of Education, School of Pharmaceutical Engineering, Shenyang Pharmaceutical University, Shenyang 110016, China; zzp2354172@gmail.com (Z.-P.Z.); zymc666@gmail.com (Y.Z.); wuwudiyi@gmail.com (Z.-B.H.); zevangeline101@gmail.com (L.Z.); huashengsu375@gmail.com (H.-S.S.); 102030118@syphu.edu.cn (J.W.)

**Keywords:** saccharide, tail approach, pH of the tumor cell microenvironment, CA IX inhibitors

## Abstract

A series of saccharide-modified thiadiazole sulfonamide derivatives has been designed and synthesized by the “tail approach” and evaluated for inhibitory activity against carbonic anhydrases II, IX, and XII. Most of the compounds showed high topological polar surface area (TPSA) values and excellent enzyme inhibitory activity. The impacts of some compounds on the viability of HT-29, MDA-MB-231, and MG-63 human cancer cell lines were examined under both normoxic and hypoxic conditions, and they showed certain inhibitory effects on cell viability. Moreover, it was found that the series of compounds had the ability to raise the pH of the tumor cell microenvironment. All the results proved that saccharide-modified thiadiazole sulfonamides have important research prospects for the development of CA IX inhibitors.

## 1. Introduction

Carbonic anhydrases (CAs, EC 4.2.1.1) are lyases that are widely distributed in all organisms from archaea to higher animals. According to the different coding genes, CAs can be divided into eight families: α-, β-, γ-, δ-, ζ-, η-, θ-, and ι-CAs. Among the eight genetically different CA families, human carbonic anhydrases (*h*CAs) belong to the α-family [1,2,3,4]. *h*CAs are a class of zinc metalloenzymes with 15 subtypes. In the human body, their main physiological roles are as catalysts for the reversible interconversion of carbon dioxide and bicarbonate [5,6,7]. CA IX is a special isoform of the *h*CAs family. Its expression in normal tissues is limited to gastrointestinal epithelial cells but it is overexpressed in many solid tumors, contributing to the growth advantage of cancer cells [8,9,10,11]. Therefore, CA IX is considered an important target for tumor treatment, and *h*CA modulators are considered promising drugs in clinical applications [12]. At present, CA IX-targeted drugs have been studied in clinical research. The CA IX inhibitor SLC-0111 combined with gemcitabine for the treatment of metastatic pancreatic ductal cancer in patients overexpressing the CA IX isoform has entered clinical phase II [13,14,15]. 

**Figure 1:**
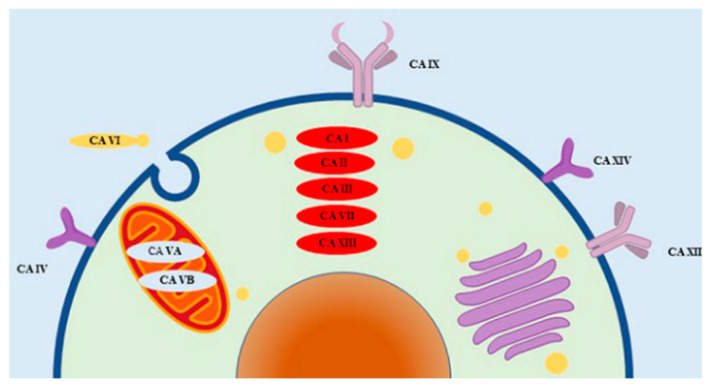
Subcellular distribution of *h*CAs.

X-ray crystallography studies showed that the catalytic domains of the *h*CA family are highly similar, which causes great difficulties for the search for selective CA inhibitors [6]. To alleviate the clinical side effects, carbonic anhydrase inhibitors such as brinzolamide, a drug for glaucoma, are administered locally [16]. Local administration is difficult for CA IX inhibitors, and the resulting side effects seriously hinder the development of selective CA IX inhibitors. Until the “tail approach” was proposed, it provided a favorable weapon for selective CA IX inhibitors [17]. The “tail approach” is a simple and effective method that refers to appending a “tail” with a wide spectrum of chemical functionalities to the Zn^2+^ binding group through a simple chemical reaction to change the mode of action of the inhibitor in different isoforms of CAs. A proper “tail” structure is of great significance for the research on selective CA IX inhibitors. One of the characteristics of CA IX is that its subcellular localization is outside the cell membrane, while its common off-target isoform CA II is located in the cytoplasm (Figure 1). The “tail” modification of appropriate fragments can improve the polarity of inhibitors to reduce cell membrane permeability, which is conducive to obtaining CA IX selectivity. Saccharides have the characteristics of low toxicity, high polarity, and high solubility. In the area of medicinal chemistry, glycol derivatives and glycosyl modification compounds have shown a wide range of pharmacological activities [18,19,20,21,22]. In the research on CA IX inhibitors, saccharide-modified compounds have achieved remarkable success, and various molecules of saccharide-modified CA IX inhibitors have been reported [23,24,25]. The high polarity of saccharide-modified compounds puts them at a disadvantage when they penetrate the cell membrane, which is conducive to the selectivity. At the same time, the hydrogen bonding between the saccharide fragment and the residues in the hydrophilic pocket at the rim of the enzymatic cavity is beneficial to improvement of the activity.

To investigate the application of saccharide modifications in CA IX inhibitors, we designed and synthesized sixteen novel compounds in this work by linking glucose fragments and thiadiazole sulfonamides. These compounds were then tested for their inhibitory effects on the tumor-associated proteins *h*CA IX and XII and off-target *h*CA II subtypes. Their inhibitory activities were comparable to that of the positive control AZM in the low nanomolar region. The study of molecular docking studies and the summary of preliminary structure–activity relationships have provided a basis for further research.

## 2. Results and Discussion

### 2.1. Chemistry

**Scheme 1:**
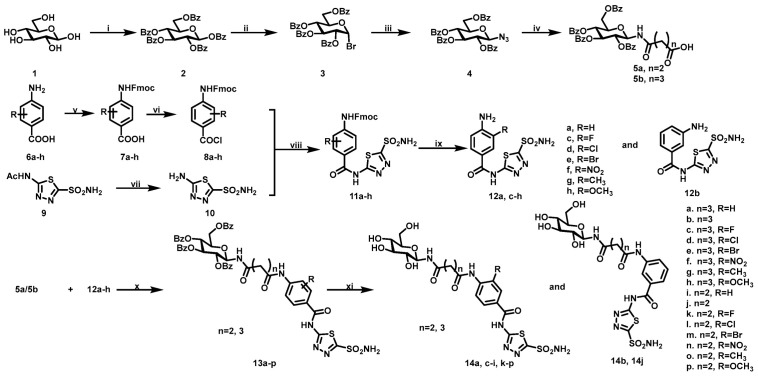
The general synthesis route of compounds **14a–p**. Reagents and conditions: (i) BzCl, 4-dimethylaminopyridine (DMAP), Py, r.t.; (ii) HBr-HOAc, dichloromethane (DCM), 0 °C -r.t.; (iii) NaN_3_, *N, N*-Dimethylformamide (DMF), 50 °C; (iv) Pd/C, H_2_, succinic anhydride/glutaric anhydride, r.t.; (v) Tetrahydrofuran (THF)/CHCl_3_, Fmoc-Cl, 60 °C; (vi) SOCl_2_, reflux; (vii) EtOH, HCl, reflux; (viii) acetone, Py, 0 °C-r.t.; (ix) diethylamine, DMF, r.t.; (x) *N*-ethyl-*N*′-(3-dimethylaminopropyl)carbodiimide hydrochloride (EDCI), Py, r.t.; (xi) MeOH/Et_3_N/H_2_O, r.t.

The general synthetic route for the synthesis of the target saccharide-modified thiadiazole sulfonamide derivatives is shown in Scheme 1. We adopted a convergent strategy to complete the syntheses of compounds, which mainly include two parts: saccharide fragments and small molecule fragments. In the saccharide fragment, glucose was chosen as the starting material, and intermediate **4** was synthesized from glucose by benzoylation (**2**), bromination (**3**) and azide substitution. After hydrogen reduction with Pd/C as the catalyst, the product underwent a one pot reaction with succinic anhydride or glutaric anhydride to generate saccharide intermediates **5a** and **5b**. In the small molecule fragment, different substituted aminobenzoic acids **6a–h** were converted into the corresponding acid chlorides after protection of the amino groups. Acid chlorides **8a–h** reacted with intermediate **10** to give the corresponding intermediates **11a–h**. Intermediates **11a–h** were deprotected to obtain small molecule intermediates **12a–h**.

In the presence of EDCI, saccharide intermediates (**5a**/**5b**) and small molecule intermediates (**12a–h**) were condensed to obtain intermediates **13a–p**. Finally, the target compounds **14a–p** were obtained by deprotection (some compounds contained a few amounts of stereoisomers).

### 2.2. Carbonic Anhydrase Inhibition

The inhibitory activities of the novel saccharide-modified thiadiazole sulfonamide derivatives **14a–p** and small molecule intermediates **12a–h** toward three physiologically relevant *h*CA isoforms, *h*CA II (common off-target cytoplasmic isoform), *h*CA IX, and XII (tumor-associated membrane-bound isoforms), were evaluated by monitoring the hydrolysis of 4-nitrophenyl acetate (4-NPA). AZM was chosen as the positive control drug. The inhibition data are summarized in Table 1.

Referring to the data in Table 1, the structure–activity relationships (SAR) of intermediates **12a–h** and compounds **14a–p** are preliminarily summarized below.

(i). The IC_50_ values of intermediates **12a–h** against *h*CA II and IX were 79.1–701.3 and 36.4–65.9 nM, respectively. Among them, **12b** showed the strongest inhibitory activity against *h*CA II, with an IC_50_ of 79.1 nM. The substitution of halogen or electron withdrawing groups led to a decrease in *h*CA II inhibitory activity. Intermediate **12h** showed the strongest inhibitory activity with *h*CA IX, with an IC_50_ of 36.4 nM. The effect of different substitutions on *h*CA IX inhibitory activity was not obvious. In the in vitro enzyme inhibitory activity test, **12a–h** generally showed selectivity for *h*CA IX, and **12f** exhibited the best selectivity for *h*CA IX; its inhibitory activity for *h*CA IX was 10 times higher than that for *h*CA II;
(ii). The IC_50_ values of saccharide-modified compounds **14a–p** against *h*CA II and IX were 80.3–1403.1 and 29.0–1082.0 nM, respectively; **14b** exhibited the strongest inhibitory activity against *h*CA II, with an IC_50_ of 80.3 nM, while **14a** and **14b** had the best inhibitory activity against *h*CA IX, with an IC_50_ of 29.0 nM. Compared with **12a–h**, the TPSA values of compounds **14a–p** were significantly increased, which was beneficial to improving the selectivity through the differences in subcellular localization of isoforms. However, the inhibitory activity against *h*CA II and IX decreased to varying degrees, and the inhibitory activity against *h*CA II decreased significantly;
(iii). Among the compounds **14a–p**, **14a–b** and **14i–j**, which did not feature substitution on the benzene ring, were the compounds with the best *h*CA II inhibitory activities when the aliphatic chain lengths were n = 2 and 3, respectively. Whether the benzene ring was substituted by an electron-donating group or electron-donating group, the inhibitory activity of the compounds against *h*CA II decreased, especially for the substituents Cl, Br and NO_2_. The activity data showed that for *h*CA II, the inhibitory activities of compounds **14a–h** with aliphatic chain length n = 3 were generally higher than those of compounds **14i–p** with n = 2;
(iv). Compared with *h*CA II, compounds **14a–p** generally had higher inhibitory activity against *h*CA IX, among which **14a–c**, **14g–j** and **14p** exhibited more desirable IC_50_ values of less than 100 nM. When the benzene ring was substituted by F or an electron donating group, the inhibitory activity of the compounds toward *h*CA IX did not change significantly, but when the substitution was for Cl, Br, or -NO_2_, the inhibitory activity was obviously reduced. Similar to *h*CA II, the inhibitory activities toward *h*CA IX for the compounds with aliphatic chain length n = 3 were higher than those of the compounds with n = 2;
(v). Compounds **14a–c**, **14g–j**, and **14p** with good inhibitory activity with *h*CA IX were tested for their inhibitory activities with *h*CA XII. The values of IC_50_ ranged from 40.6 to 140.8 nM. All tested compounds showed strong *h*CA XII inhibitory activity, of which that of **14j** was the most prominent.

In summary, this series of compounds showed significant inhibition against the tumor-related isoforms *h*CA IX and XII, with high TPSA and potential selective qualifications, which are worthy of further study.

### 2.3. Studies on Docking into the Active Site of hCA IX

**Figure 2:**
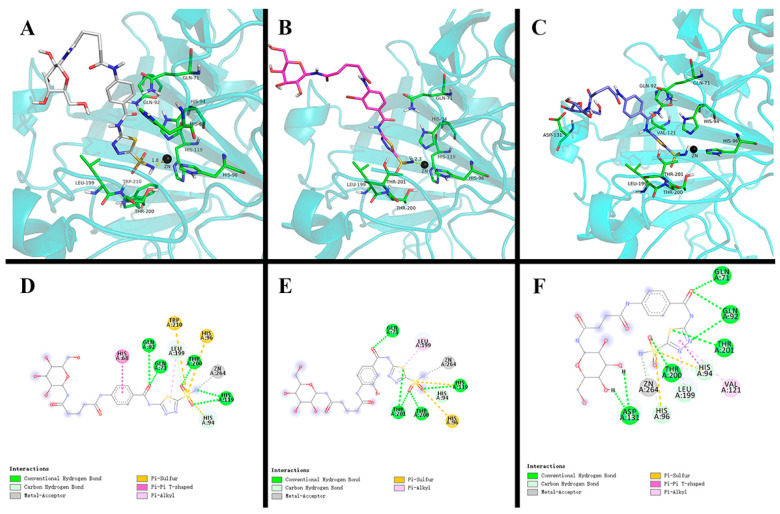
The modes of binding for compounds in *h*CA IX (**A**,**D**); compound **14a** (grey) (**B**,**E**); compound **14e** (pink) (**C**,**F**); compound **14i** (blue).

We firstly re-docked the native ligands into CA IX (PDB ID: 5FL4) with optimized docking parameters, and successfully produced the binding modes observed in the crystal structures, with RMSD values of 0.619 Å for the docked and experimental poses. As compounds **14a**, **14e**, and **14i** were CA IX inhibitors; their binding mode to the active site of CA IX was investigated by a docking simulation using Autodock 4(Zn). The results are shown in Figure 2.

By analyzing the docking results of **14a**, the oxygen atom of benzamide formed two hydrogen bonds interaction with Gln71 and Gln92 in CA IX (Figure 2A,D). The sulfonamide group was positioned in the bottom of the cavity, interacting strongly H-bonding interactions with His119 and Thr200, respectively. The ring of benzamide formed strong π–π stacking interaction with His68. Meanwhile, the region of the thiazole sulfonamide formed hydrophobic interactions with His94, His96, Leu199, and Trp210. In the docking results of **14e** (Figure 2B,E), the oxygen atom of benzamide formed two hydrogen bonds interaction with Gln71 in CA IX. The thiazolesulfonamide group was positioned in the bottom of the cavity, having strong H-bonding interactions with His119, Thr200, and Thr201, respectively. Meanwhile, the thiazolesulfonamide group also formed hydrophobic interactions with His94, His96 and Leu199. In the docking results of **14i** (Figure 2C,F), the oxygen atom of hydroxyl formed hydrogen bonds interaction with Asp131. The oxygen atom of benzamide interacted H-bonding interactions with Gln71 and Gln92. The thiadiazole group locating in the middle of the cavity formed hydrogen bonds interaction with Gln92 and Thr201. The sulfonamide group was positioned in the bottom of the cavity, interacting strongly H-bonding interactions with Thr200. The ring of thiadiazole formed strong π–π stacking interaction with His94. At the same time, the region of the thiazolesulfonamide formed hydrophobic interactions with His94, His96, Val121, and Leu199. Importantly, the sulfonamide group formed a coordination bond with the zinc atom, which was reflected in the three compounds playing a crucial role in the activity. Afterwards, in order to verify the accuracy of the docking, we conducted dynamic simulations on them.

### 2.4. Molecular Dynamics Simulations

The 100 ns Molecular dynamics (MD) simulations were performed with three selected CA IX-compound complexes (**14a**, **14e**, and **14i**) to examine their interactions between receptors and ligands and elucidate the contribution of key residues during the binding process.

**Figure 3:**
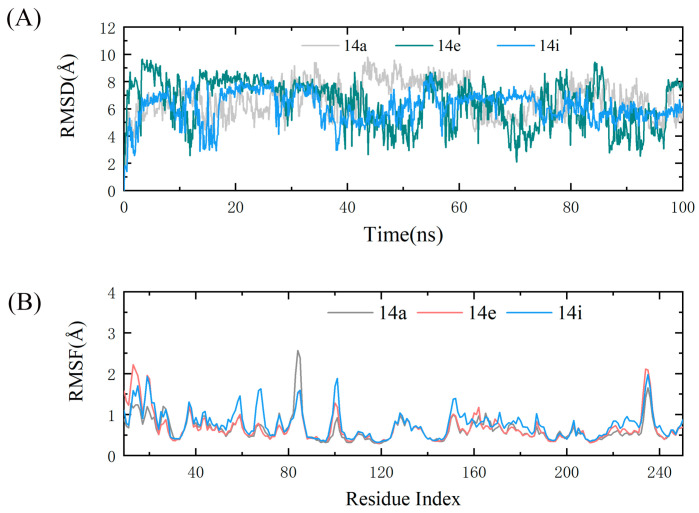
The Root-mean-square deviation (RMSD) and Root-mean-square fluctuations (RMSF) of CA IX protein during the whole MD simulations. (**A**) RMSD plots of CA IX-compound **14a** complex (grey), CA IX-compound **14e** complex (green) and CA IX-compound **14i** complex (blue). (**B**) RMSF plots of CA IX-compound **14a** (grey), CA IX-compound **14e** complex (orange) and CA IX-compound **14i** complex (blue).

As shown in Figure 3, the RMSD and RMSF values of three complexes were monitored during the whole MD simulations. In Figure 3A, the CA IX-compound **14a** complex reached equilibrium after 65 ns, where the protein RMSD value fluctuated around 5.5–7.0 Å. The CA IX-compound **14i** complex reached equilibrium after 60 ns, where the protein RMSD value fluctuated around 6.0–7.0 Å. Throughout the MD simulation, **14e** showed obvious fluctuations and did not reach equilibrium, indicating that **14e** had poor binding affinity and the result was consistent with enzyme inhibitory activity. The RMSF plots were displayed in Figure 3B, the fluctuations of majority residues were less than 2 Å, and several regions with huge fluctuations located in the active site.

**Figure 4:**
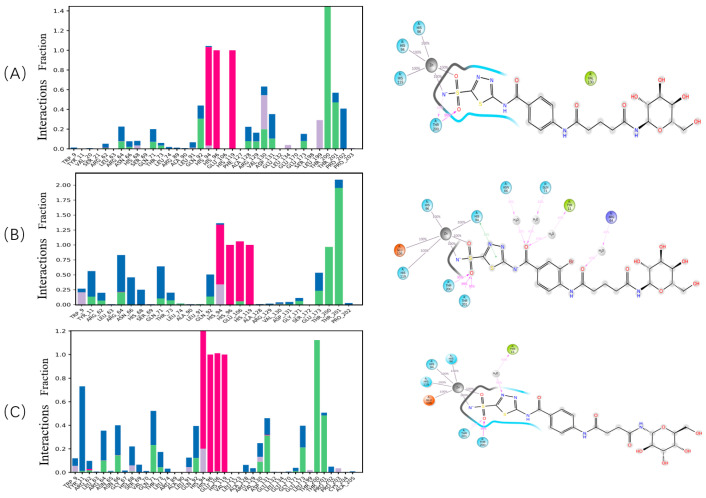
Protein-ligand contacts during the whole MD simulations. (**A**) Interaction fraction of CA IX-compound **14a** complex. (**B**) Interaction fraction of CA IX-compound **14e** complex. (**C**) Interaction fraction of CA IX-compound **14i** complex.

As shown in Figure 4, the histogram showed the type and ratio of interactions between receptors and ligands, including hydrogen bonds, hydrophobic contacts, ionic interactions, and water bridges. In Figure 4A, compound **14a** formed hydrogen bonds with residues Arg64, Gln71, Gln92, Ala128, Arg129, Val130, Asp131, Glu173, Thr200, and Thr201, and formed water bridges with residues Arg64, Gln71, Gln92, Ala128, Arg129, Val130, Asp131, and Pro202. The hydrophobic interactions were with residues Val130 and Leu199. Among them, **14a** formed strong hydrogen bonds with Thr200, which accounted for 99% of the simulation time, which played a crucial role in activities. In Figure 4B, compound **14e** formed hydrogen bonds with residues Tyr11, Arg64, Gln71, Gln92, Glu173, Thr200, and Thr201, and formed water bridges with residues Tyr11, Arg64, Asn66, Asn68, Gln71, Gln92, Glu173, and Thr201. The hydrophobic interactions were with residues Trp9 and His94. Among them, **14e** formed strong hydrogen bonds with Thr200, which accounted for 99% of the simulation time. In addition, water bridges were also significant for the interactions, such as the carbonyl oxygen formed water bridges with Tyr11, Arg64, Asn66, and Gln71, which accounted for 40%, 40%, 35% and 42% of the simulation time, respectively. In Figure 4C, compound **14i** formed hydrogen bonds with residues Arg64, Asn66, Gln71, Gln92, Val130, Asp131, Glu173, Thr200, and Thr201, and formed water bridges with residues Tyr11, Arg64, Asn66, Asn68, Gln71, Gln92, Val130, Asp131, and Glu173. The hydrophobic interactions were with residues Trp9, His94, and Val130. Among them, **14i** formed strong hydrogen bonds with Thr200, which accounted for 99% of the simulation time. Meanwhile, residue Tyr 11 formed water bridge with **14i** accounting for 50% of the simulation time.

The results of MD simulations indicated **14a** and **14i** were more stable during MD simulations and revealing that **14a** and **14i** might have stronger interactions with CA IX. In addition, compound **14e** had the largest RMSD fluctuation, this might be due to its weak contact with CA IX which made the complex unstable, and could explain its lowest activity among three compounds.

### 2.5. In Vitro Cytotoxicity Studies on Cancer Cells

**Figure 5:**
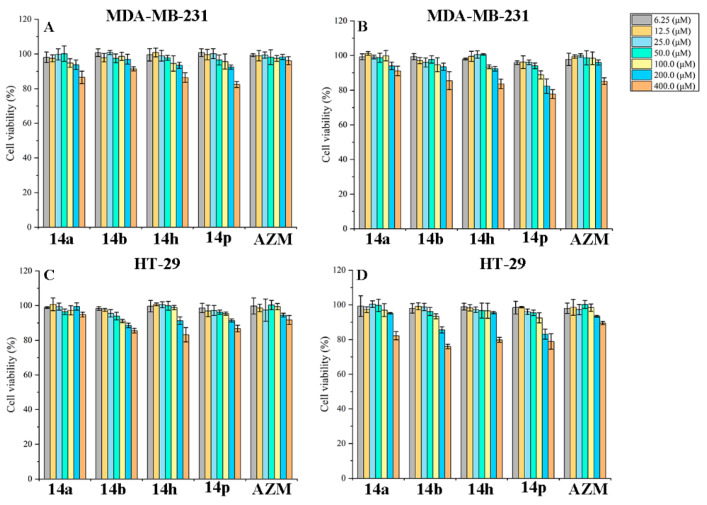
Effects of compounds **14a**, **14b**, **14h**, **14p**, and AZM on the cell viability of HT-29 and MDA-MB-231 under normoxic (**A**,**C**) and hypoxic (**B**,**D**) conditions.

To evaluate the ability of novel saccharide-modified compounds to inhibit the viability of tumor cells, several compounds that exhibited the best inhibitory profiles were chosen to evaluate their effects against human colon cancer HT-29 cells and breast adenocarcinoma MDA-MB-231 cells via CCK-8 assay. The results are shown in Figure 5.

As shown in Figure 5, compounds **14a**, **14b**, **14h**, **14p**, and AZM were evaluated for their effects against two cancer cell lines that overexpress CA IX under normoxic and hypoxic conditions. Based on the results, we summarize the following conclusions:

(i). The inhibitory activities of compounds **14a**, **14b**, **14h**, and **14p** on the two tumor cell lines were better than those of the positive control drug AZM;
(ii). Under hypoxic conditions, the inhibitory activities of the tested compounds and AZM on the tumor cell lines were all higher than those under normoxia;
(iii). Compared with the breast adenocarcinoma MDA-MB-231, the tested compounds generally showed higher inhibitory efficacy with the colon cancer cell line HT-29.

**Figure 6:**
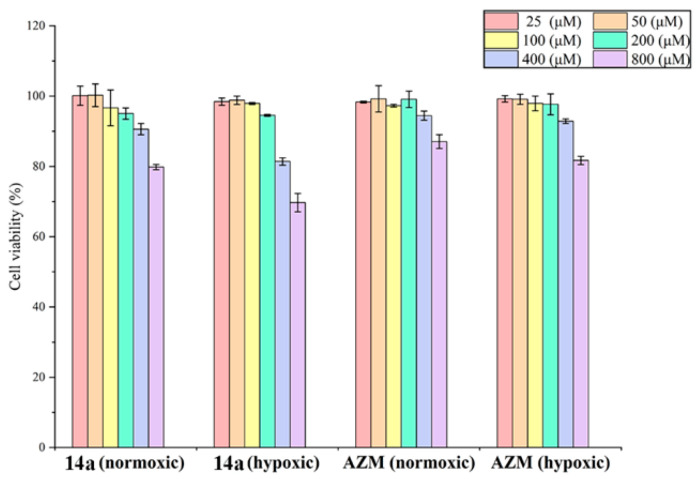
Effects of compounds **14a** and AZM on the cell viability of MG-63 under normoxic and hypoxic conditions.

Then, considering the high inhibitory activity of **14a** on the two isoforms of CA IX and XII, we evaluated the inhibitory activity of **14a** on human osteosarcoma MG-63 featuring high expression of CA IX and XII. The results are shown in Figure 6.

Similar to the previous conclusions, the effect of **14a** in inhibiting the vitality of MG-63 cells under hypoxic conditions was more obvious than that under normoxic conditions. Under hypoxic conditions, the administration of 800 μM 14**a** reduced the viability of MG-63 cells by approximately 30%, and the effect was significantly better than that of the positive control AZM.

Although these novel saccharide-modified CA IX inhibitors showed promising isoform inhibitory activities, their inhibitory effect on the cell viability of tumor-related cells was not obvious. This is a common problem in research on CA IX inhibitors [26,27]. This might indicate that CA IX inhibitors are not effective enough to be used alone in cancer therapy [25].

### 2.6. Extracellular pH Measurement in the Presence of Compound ***14b***, ***14h***, and ***14p***

**Figure 7:**
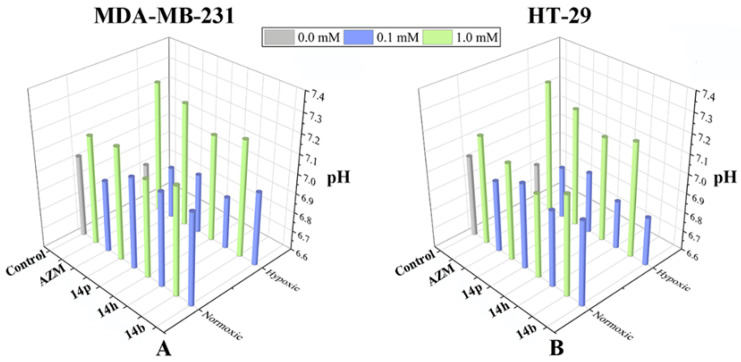
Extracellular pH measurement of MDA-MB-231 (**A**) and HT-29 (**B**) under normoxic and hypoxic conditions.

The high expression of CA IX plays an important role in the maintenance of the tumor cell microenvironment, and inhibiting its activity might have a potential impact on the pH of the tumor microenvironment. To evaluate the effect of compounds on the pH of the microenvironment, the pH values under normoxic and hypoxic conditions were determined in the presence of compounds **14b**, **14h**, and **14p**. The results showed that **14b**, **14h**, and **14p** increased tumor pH to varying degrees at concentrations of 0.1 and 1 mM (Figure 7). The tested compounds had no obvious effect on cells under normal conditions, but they significantly improved extracellular acidosis induced by hypoxia. In addition, this improvement in pH was dose-dependent.

## 3. Materials and Methods

### 3.1. Chemistry

All the reagents (Energy Chemical, Shanghai, China) were used without further purification unless otherwise specified. Solvents were dried and redistilled prior to use in the usual manner. Analytical TLC was per-formed using silica gel HF_254_ (Qingdao Haiyang Chemical, Qingdao, Shandong, China). Preparative column chromatography was performed with silica gel H. Melting points were obtained on a Büchi melting point B-540 apparatus (Büchi Labortechnik, Flawil, Switzerland). ^1^H and ^13^C NMR spectra (details of raw data for compounds, see Figures S1–S54) were recorded on a Bruker ARX 600 or 400 MHz spectrometer (Bruker, Zurich, Switzerland) HRMS were obtained on an Agilent ESI-QTOF instrument (Agilent, Santa Clara, CA, USA).

#### 3.1.1. Synthesis of Intermediate **2**–**4**

Intermediates **2–4** were prepared according to the literature procedures [28]. Intermediate 4 [α]${}_{D}^{20}$ +22.7 (*c* 1.0, acetone).

#### 3.1.2. General Procedure of (((2R,3R,4S,5R,6R)-3,4,5-Tris(benzoyloxy)-6-((benzoyloxy)methyl)tetrahydro-2H-pyran-2-yl)amino) Acid (**5a** and **5b**)

Intermediate **4** (1.50 g, 2.4 mmol), corresponding acid anhydride (2.4 mmol), 4-dimethylaminopyridine (15.00 mg, 0.12 mmol) and Pd/C (0.15 g) were dispersed in 45 mL THF. Then, in the presence of hydrogen, the reaction was stirred at room temperature for 6 h. After the reaction was completed, the solution was concentrated under reduced pressure. Purification by column chromatography (75: 1, dichloromethane-methanol) to obtain intermediate **5a** and **5b**.

##### 4-Oxo-4-(((2*R*,3*R*,4*S*,5*R*,6*R*)-3,4,5-tris(benzoyloxy)-6-((benzoyloxy)methyl)tetrahydro-2*H*-pyran-2-yl)amino)butanoic Acid (**5a**)

Yield 64.1%. m.p. 88.2–90.0 °C, [α]${}_{D}^{20}$ +39.7 (*c* 1.0, acetone), ^1^H-NMR (DMSO-*d_6_*, 600 MHz) *δ* 12.12 (s, 1H), 8.93 (d, *J* = 9.5 Hz, 1H), 7.97 (d, *J* = 7.2 Hz, 2H), 7.84 (d, *J* = 7.3 Hz, 2H), 7.79 (d, *J* = 7.5 Hz, 2H), 7.72 (d, *J* = 7.4 Hz, 2H), 7.69 (t, *J* = 7.2 Hz, 1H), 7.61 (dd, *J* = 16.8, 4.1 Hz, 2H), 7.55 (q, *J* = 7.3 Hz, 3H), 7.46 (dd, *J* = 9.4, 6.1 Hz, 4H), 7.41 (t, *J* = 7.7 Hz, 2H), 6.06 (t, *J* = 10.1 Hz, 1H), 5.81 (t, *J* = 9.3 Hz, 1H), 5.60 (t, *J* = 9.7 Hz, 1H), 5.35 (t, *J* = 9.4 Hz, 1H), 4.61 (d, *J* = 9.8 Hz, 1H), 4.52 (d, *J* = 11.1 Hz, 1H), 4.46 (dd, *J* = 12.4, 3.5 Hz, 1H), 2.29 (tdd, *J* = 23.4, 20.7, 7.6 Hz, 4H) ppm. ^13^C-NMR (DMSO-*d_6_*, 151 MHz) *δ* 173.92, 173.08, 172.26, 165.80, 165.54, 165.25, 134.21, 133.99, 129.64, 129.25, 129.03, 77.75, 74.40, 72.73, 72.16, 69.39, 62.92, 30.47, 29.14 ppm.

##### 5-Oxo-5-(((2*R*,3*R*,4*S*,5*R*,6*R*)-3,4,5-tris(benzoyloxy)-6-((benzoyloxy)methyl)tetrahydro-2*H*-pyran-2-yl)amino)pentanoic Acid (**5b**)

Yield 70.0%.m.p. 94.4–96.8 °C, [α]${}_{D}^{20}$ +51.0 (*c* 1.0, acetone), ^1^H-NMR (DMSO-*d*_6_, 600 MHz) δ 12.03 (s, 2H), 8.87 (d, J = 9.5 Hz, 1H), 7.97 (dd, J = 8.2, 1.2 Hz, 2H), 7.84 (dd, J = 8.3, 1.1 Hz, 2H), 7.79 (dd, J = 8.3, 1.2 Hz, 2H), 7.72 (dd, J = 8.2, 1.1 Hz, 2H), 7.70–7.65 (m, 1H), 7.61 (td, J = 7.4, 1.6 Hz, 2H), 7.55 (q, J = 8.0 Hz, 3H), 7.46 (dd, J = 14.0, 7.6 Hz, 4H), 7.41 (t, J = 7.8 Hz, 2H), 6.06 (t, J = 9.5 Hz, 1H), 5.81 (t, J = 9.4 Hz, 1H), 5.59 (t, J = 9.7 Hz, 1H), 5.36 (t, J = 9.4 Hz, 1H), 4.64–4.57 (m, 1H), 4.52 (dd, J = 12.4, 2.4 Hz, 1H), 4.45 (dd, J = 12.5, 3.8 Hz, 1H), 2.25 (t, J = 7.4 Hz, 3H), 2.17–2.02 (m, 4H) ppm. ^13^C-NMR (DMSO-*d*_6_, 151 MHz) δ 174.56, 172.88, 165.79, 165.53, 165.23, 134.50, 134.11, 133.99, 129.81, 129.65, 129.48, 129.15, 129.02, 77.66, 74.36, 72.77, 72.14, 69.43, 62.95, 60.23, 34.83, 33.22, 20.75 ppm.

#### 3.1.3. General Procedure of 4-((((9H-Fluoren-9-yl)methoxy)carbonyl)amino)benzoic Acid Derivatives (**7a–h**)

To a solution of different aminobenzoic acid (1.0 mmol) in THF (20 mL), Fmoc-Cl (3.88 g, 1.5 mmol) in CHCl_3_ was added by drop. After the addition was complete, reacted at 60 °C for 12 h. Once the reaction was complete, the reaction solution was cooled to room temperature, 20 mL of ether was added, filtered, and the solid was collected to obtain intermediate **7a–h** with a yield of 77.2–86.5%. The products were directly used without purification.

#### 3.1.4. General Procedure of (9H-Fluoren-9-yl)methyl (4-(chlorocarbonyl)phenyl)carbamate Derivatives (**8a**–**h**)

Intermediate **7a–h** was dispersed in 20 mL of SOCl_2_, and the reaction was refluxed for 5 h until the reaction solution was clear. Then, the reaction solution was cooled to room temperature, and the solvent was removed under reduced pressure to obtain intermediate **8a–h** with a yield of 88.2–93.8%. The products were directly used without purification.

#### 3.1.5. Synthesis of 5-Amino-1,3,4-thiadiazole-2-sulfonamide (**10**)

Acetazolamide (5.00 g, 22.5 mmol) was dispersed in 50 mL of EtOH, 8 mL of concentrated hydrochloric acid was added, and the reaction was refluxed for 8 h until the reaction solution was clear. After cooling to room temperature, the solvent was removed under reduced pressure. Slowly added sodium bicarbonate solution until the pH of solution was neutral. Filtrated, collected solids to obtain 3.77 g intermediates **10** with a yield of 93.0%. ^1^H-NMR (600 MHz, DMSO-*d*_6_) *δ* 8.06 (s, 2H), 7.81 (s, 2H). ^13^C-NMR (151 MHz, DMSO-*d*_6_) *δ* 172.13, 158.34.

#### 3.1.6. General Procedure of (9H-Fluoren-9-yl)methyl (4-((5-sulfamoyl-1,3,4-thiadiazol-2-yl)carbamoyl)phenyl)carbamate Derivatives (**11a**–**h**)

Intermediates **10** (1.80 g, 1.0 mmol) was dispersed in 30 mL of acetone, 0.24 mL pyridine was added, then intermediate **7a–h** was added gradually. The mixture was stirred at room temperature for 8 h. After the reaction was completed, filtered and collected the solid to obtain intermediate **11a–h** with a yield of 52.6–73.9%. The products were directly used without purification.

#### 3.1.7. General Procedure of 4-Amino-N-(5-sulfamoyl-1,3,4-thiadiazol-2-yl)benzamide Derivatives (**12a–h**)

To a solution of intermediates **11a–h**, 5 mL of diethylamine was added, and the reaction was stirred at room temperature for 5 h. After the reaction was completed, the solution was concentrated under reduced pressure. Then, purification by column chromatography (25:1, dichloromethane-methanol) to obtain intermediates **12a–h**.

##### 4-Amino-*N*-(5-sulfamoyl-1,3,4-thiadiazol-2-yl)benzamide (**12a**)

Yield 64.1%, ^1^H-NMR (600 MHz, DMSO-*d_6_*) *δ*13.30 (s), 8.34 (s), 7.31 (d, *J* = 7.7 Hz), 7.26 (s), 7.20 (t, *J* = 7.8 Hz), 6.85 (dd, *J* = 7.9, 1.4 Hz), 5.46 (s). ^13^C-NMR (151 MHz, DMSO-*d_6_*) *δ* 165.61, 164.02, 161.51, 148.48, 130.86, 128.58, 118.01, 114.82, 112.86.

##### 3-Amino-*N*-(5-sulfamoyl-1,3,4-thiadiazol-2-yl)benzamide (**12b**)

Yield 59.7%, ^1^H-NMR (600 MHz, DMSO-*d_6_*) *δ*13.30 (s), 8.34 (s), 7.31 (d, *J* = 7.7 Hz), 7.26 (s), 7.20 (t, *J* = 7.8 Hz), 6.85 (dd, *J* = 7.9, 1.4 Hz), 5.46 (s). ^13^C-NMR (151 MHz, DMSO-*d_6_*) *δ* 165.61, 164.02, 161.51, 148.48, 130.86, 128.58, 118.01, 114.82, 112.86.

##### 4-Amino-3-fluoro-*N*-(5-sulfamoyl-1,3,4-thiadiazol-2-yl)benzamide (**12c**)

Yield 59.7%, ^1^H-NMR (600 MHz, DMSO-*d_6_*) *δ* 13.06 (s), 8.31 (s), 7.88 (dd, *J* = 12.7, 1.8 Hz), 7.80 (dd, *J* = 8.5, 1.9 Hz), 6.83 (t, *J* = 8.7 Hz), 6.23 (s). ^13^C-NMR (151 MHz, DMSO-*d_6_*) *δ* 164.77, 164.63, 163.05, 150.38, 148.80, 142.64, 142.56, 127.00, 115.84, 115.71, 115.23, 115.20.

##### 4-Amino-3-chloro-*N*-(5-sulfamoyl-1,3,4-thiadiazol-2-yl)benzamide (**12d**)

Yield 48.1%, ^1^H-NMR (400 MHz, DMSO-*d_6_*) *δ*13.09 (s, 1H), 8.29 (s, 2H), 8.13 (s, 1H), 7.88 (dd, *J* = 8.6, 2.1 Hz, 1H), 6.86 (d, *J* = 8.6 Hz, 1H), 6.37 (s, 2H). ^13^C-NMR (151 MHz, DMSO-*d_6_*) *δ* 164.86, 164.38, 162.89, 150.03, 130.61, 129.51, 118.28, 116.63, 114.66.

##### 4-Amino-3-bromo-*N*-(5-sulfamoyl-1,3,4-thiadiazol-2-yl)benzamide (**12e**)

Yield 37.0%, ^1^H-NMR (600 MHz, DMSO-*d_6_*) *δ*13.10 (s), 8.45–8.24 (m), 7.90 (dd, *J* = 8.6, 2.1 Hz), 6.85 (d, *J* = 8.6 Hz), 6.32 (s). ^13^C-NMR (151 MHz, DMSO-*d_6_*) *δ* 164.69, 164.39, 163.15, 151.01, 133.85, 129.99, 114.55, 106.53.

##### 4-Amino-3-nitro-*N*-(5-sulfamoyl-1,3,4-thiadiazol-2-yl)benzamide (**12f**)

Yield 42.4%, ^1^H-NMR (600 MHz, DMSO-*d_6_*) *δ* 13.49 (s), 8.99 (d, *J* = 2.1 Hz), 8.35 (s), 8.13–8.02 (m), 7.11 (d, *J* = 9.0 Hz). ^13^C-NMR (151 MHz, DMSO-*d_6_*) *δ* 165.04, 164.18, 162.77, 149.40, 135.84, 134.93, 130.18, 128.47, 119.81.

##### 4-Amino-3-methyl-*N*-(5-sulfamoyl-1,3,4-thiadiazol-2-yl)benzamide (**12g**)

Yield 57.3%, ^1^H-NMR (400 MHz, DMSO-*d_6_*) *δ* 12.86 (s, 1H), 8.27 (s, 2H), 7.85 (d, *J* = 1.5 Hz, 1H), 7.80 (dd, *J* = 8.5, 2.1 Hz, 1H), 6.66 (d, *J* = 8.5 Hz, 1H), 5.90 (s, 2H), 2.11 (s, 3H). ^13^C-NMR (151 MHz, DMSO-*d_6_*) *δ* 165.23, 164.68, 162.98, 152.64, 131.56, 128.78, 120.51, 116.93, 113.17, 17.83.

##### 4-Amino-3-methoxy-*N*-(5-sulfamoyl-1,3,4-thiadiazol-2-yl)benzamide (**12h**)

Yield 63.0%, ^1^H-NMR (600 MHz, DMSO-*d_6_*) *δ* 13.02 (s), 8.26 (s), 7.64 (d, *J* = 10.2 Hz), 6.69 (d, *J* = 8.0 Hz), 5.77 (s), 3.87 (s). ^13^C-NMR (151 MHz, DMSO-*d_6_*) *δ* 165.61, 164.29, 163.75, 145.58, 124.17, 112.55, 110.64, 55.99.

#### 3.1.8. General Procedure of (2R,3R,4S,5R,6R)-2-((Benzoyloxy)methyl)-6-(4-oxo-4-((4-((5-sulfamoyl-1,3,4-thiadiazol-2-yl)carbamoyl)phenyl)amino)butanamido)tetrahydro-2H-pyran-3,4,5-triyl Tribenzoate Derivatives (**13a–p**)

Intermediate **5a (b)** (1.0 mmol) and EDCI (0.29 g, 1.5 mmol) were dissolved in 25 mL of anhydrous pyridine. After stirring for 1 h, corresponding intermediate **12a–h** (1.0 mmol) was added and reacted at room temperature for 8 h. After the reaction completed, the solution was diluted with ethyl acetate, then the mixture was washed with water and saturated sodium chloride solution successively. The solution was dried over Na_2_SO_4_, filtered, and concentrated to obtain the intermediate **13a–h**. The products were directly used without purification.

#### 3.1.9. General Procedure of *N*^1^-(4-((5-Sulfamoyl-1,3,4-thiadiazol-2-yl)carbamoyl)phenyl)-*N*^5^-((2R,3R,4S,5S,6R)-3,4,5-trihydroxy-6-(hydroxymethyl)tetrahydro-2H-pyran-2-yl)glutaramide Derivatives (**14a**–**p**)

Dissolved **13a–p** in 22 mL of a mixed solution of methanol/triethylamine/water (8:2:1) and reacted at room temperature for 12 h. After the reaction completed, the solvent was removed under reduced pressure, and the obtained solid was separated by column chromatography (dichloromethane: methanol = 8:1) to obtain compound **14a–p**, some compounds contained a small amount of stereoisomer that were difficult to separate.

##### *N*^1^-(4-((5-Sulfamoyl-1,3,4-thiadiazol-2-yl)carbamoyl)phenyl)-*N*^5^-((2*R*,3*R*,4*S*,5*S*,6*R*)-3,4,5-trihydroxy-6-(hydroxymethyl)tetrahydro-2*H*-pyran-2-yl)glutaramide (**14a**)

Two-step yield 12.2%, ^1^H-NMR (600 MHz, DMSO-*d_6_*) *δ* 10.05 (s, 1H), 8.35 (d, *J* = 9.1 Hz, 1H), 8.24 (s, 1H), 8.07 (d, *J* = 8.7 Hz, 2H), 7.74 (s, 2H), 7.64 (d, *J* = 8.6 Hz, 2H), 4.72 (t, *J* = 9.1 Hz, 1H), 3.64 (dd, *J* = 11.7, 1.8 Hz, 1H), 3.41 (dd, *J* = 11.8, 5.4 Hz, 1H), 3.17 (t, *J* = 8.8 Hz, 1H), 3.12–2.99 (m, 3H), 2.36 (dd, *J* = 13.9, 6.6 Hz, 2H), 2.18 (ddq, *J* = 22.5, 15.0, 7.5 Hz, 2H), 1.93–1.72 (m, 2H). ^13^C-NMR (151 MHz, DMSO-*d_6_*) *δ* 172.84, 171.62, 170.62, 159.85, 141.55, 132.95, 129.54, 129.22, 118.37, 79.82, 78.87, 77.72, 72.69, 70.30, 61.29, 36.17, 34.99, 21.22.; HRMS (ESI): Calcd. for [M − H]^−^ C_20_H_25_N_6_O_10_S_2_: 573.1152, Found 573.1143.

##### *N*^1^-(3-((5-Sulfamoyl-1,3,4-thiadiazol-2-yl)carbamoyl)phenyl)-*N*^5^-((2*R*,3*R*,4*S*,5*S*,6*R*)-3,4,5-trihydroxy-6-(hydroxymethyl)tetrahydro-2*H*-pyran-2-yl)glutaramide (**14b**)

Two-step yield 10.9%, ^1^H-NMR (600 MHz, DMSO-*d_6_*) *δ*13.48 (s, 1H), 10.12 (s, 1H), 8.35 (d, *J* = 2.0 Hz, 2H), 8.19 (s, 2H), 7.83 (d, *J* = 7.9 Hz, 2H), 7.45 (t, *J* = 7.9 Hz, 1H), 4.96 (d, *J* = 4.1 Hz, 1H), 4.91–4.81 (m, 2H), 4.72 (t, *J* = 9.1 Hz, 1H), 4.49 (t, *J* = 5.1 Hz, 1H), 3.64 (dd, *J* = 11.0, 3.9 Hz, 1H), 3.44–3.38 (m, 1H), 3.17 (td, *J* = 8.7, 3.5 Hz, 1H), 3.11–3.01 (m, 3H), 2.37 (t, *J* = 7.4 Hz, 2H), 2.27–2.07 (m, 2H), 1.88–1.78 (m, 2H). ^13^C-NMR (151 MHz, DMSO-*d_6_*) *δ* 174.66, 172.79, 171.57, 163.54, 139.62, 129.09, 123.39, 122.84, 119.84, 119.74, 79.81, 78.91, 77.78, 72.74, 70.31, 61.29, 36.12, 35.00, 21.25; HRMS (ESI): Calcd. for [M − H]^−^ C_20_H_25_N_6_O_10_S_2_: 573.1152, Found 573.1139.

##### *N*^1^-(2-Fluoro-4-((5-sulfamoyl-1,3,4-thiadiazol-2-yl)carbamoyl)phenyl)-*N*^5^-((2*R*,3*R*,4*S*,5*S*,6*R*)-3,4,5-trihydroxy-6-(hydroxymethyl)tetrahydro-2*H*-pyran-2-yl)glutaramide (**14c**)

Two-step yield 9.2%, ^1^H-NMR (600 MHz, DMSO-*d_6_*) *δ* 9.77 (s, 1H), 8.36 (d, *J* = 9.1 Hz, 1H), 8.25 (s, 1H), 8.05–7.97 (m, 1H), 7.92 (d, *J* = 8.4 Hz, 1H), 7.86 (d, *J* = 12.1 Hz, 1H), 7.70 (s, 2H), 4.71 (dt, *J* = 13.4, 9.1 Hz, 2H), 3.63 (d, *J* = 14.1 Hz, 2H), 3.44–3.37 (m, 2H), 3.22–3.13 (m, 2H), 3.12–3.00 (m, 4H), 2.44 (t, *J* = 7.1 Hz, 2H), 2.24–2.16 (m, 2H), 1.82 (dt, *J* = 14.7, 7.3 Hz, 2H). ^13^C-NMR (151 MHz, DMSO-*d_6_*) *δ*172.52, 171.47, 169.51, 166.33, 159.28, 153.37, 151.75, 135.45, 127.76, 124.13, 122.47, 114.77, 114.64, 79.24, 78.25, 77.11, 72.09, 69.71, 60.71, 35.10, 34.43, 20.74; HRMS (ESI): Calcd. for [M − H]^−^ C_20_H_24_FN_6_O_10_S_2_: 591.1058, Found 591.1050.

##### *N*^1^-(2-Chloro-4-((5-sulfamoyl-1,3,4-thiadiazol-2-yl)carbamoyl)phenyl)-*N*^5^-((2*R*,3*R*,4*S*,5*S*,6*R*)-3,4,5-trihydroxy-6-(hydroxymethyl)tetrahydro-2*H*-pyran-2-yl)glutaramide (**14d**)

Two-step yield 7.7%, ^1^H-NMR (600 MHz, DMSO-*d_6_*) *δ* 12.58 (s, 1H), 8.23 (d, *J* = 9.1 Hz, 1H), 8.10 (s, 1H), 7.85 (dd, *J* = 8.6, 1.8 Hz, 1H), 6.84 (d, *J* = 8.6 Hz, 1H), 6.29 (s, 2H), 4.88 (d, *J* = 41.0 Hz, 3H), 4.70 (t, *J* = 9.1 Hz, 1H), 4.54 (s, 1H), 3.62 (d, *J* = 11.7 Hz, 1H), 3.40 (d, *J* = 11.4 Hz, 3H), 3.16 (t, *J* = 8.7 Hz, 1H), 3.11–2.99 (m, 4H), 2.07 (dq, *J* = 14.6, 7.4 Hz, 2H), 2.00 (t, *J* = 7.3 Hz, 2H), 1.63 (p, *J* = 7.3 Hz, 2H). ^13^C-NMR (151 MHz, DMSO-*d_6_*) *δ*179.27, 173.47, 167.81, 164.19, 161.93, 149.53, 130.40, 129.26, 118.94, 116.62, 114.60, 79.83, 78.80, 77.63, 72.58, 70.24, 61.21, 38.60, 35.41, 21.94; HRMS (ESI): Calcd. for [M − H]^−^ C_20_H_24_ClN_6_O_10_S_2_: 607.0762, Found 607.0756.

##### *N*^1^-(2-Bromo-4-((5-sulfamoyl-1,3,4-thiadiazol-2-yl)carbamoyl)phenyl)-*N*^5^-((2*R*,3*R*,4*S*,5*S*,6*R*)-3,4,5-trihydroxy-6-(hydroxymethyl)tetrahydro-2*H*-pyran-2-yl)glutaramide (**14e**)

Two-step yield 5.9%, ^1^H-NMR (600 MHz, DMSO-*d_6_*) *δ* 8.32 (d, *J* = 9.0 Hz), 8.20 (d, *J* = 1.6 Hz), 7.86 (dd, *J* = 8.5, 1.7 Hz), 6.81 (d, *J* = 8.5 Hz), 5.99 (s), 4.69 (t, *J* = 9.0 Hz), 3.62 (d, *J* = 11.7 Hz), 3.41 (dd, *J* = 11.8, 5.0 Hz), 3.17 (t, *J* = 8.6 Hz), 3.07 (dt, *J* = 18.0, 9.3 Hz), 2.06 (dd, *J* = 15.2, 7.4 Hz), 2.00 (t, *J* = 7.2 Hz), 1.70–1.52 (m). ^13^C-NMR (151 MHz, DMSO-*d_6_*) *δ* 179.22, 173.53, 173.37, 167.99, 149.56, 133.48, 129.60, 122.88, 114.47, 106.58, 79.95, 78.85, 77.57, 72.51, 70.28, 61.27, 38.45, 35.33, 22.12; HRMS (ESI): Calcd. for [M − H]^−^ C_20_H_24_BrN_6_O_10_S_2_: 651.0257, Found 651.0250.

##### *N*^1^-(2-Nitro-4-((5-sulfamoyl-1,3,4-thiadiazol-2-yl)carbamoyl)phenyl)-*N*^5^-((2*R*,3*R*,4*S*,5*S*,6*R*)-3,4,5-trihydroxy-6-(hydroxymethyl)tetrahydro-2*H*-pyran-2-yl)glutaramide (**14f**)

Two-step yield 8.3%, ^1^H-NMR (600 MHz, DMSO-*d_6_*) *δ* 13.55 (d, *J* = 81.3 Hz, 1H), 8.99 (dd, *J* = 6.6, 2.1 Hz, 1H), 8.42–8.29 (m, 2H), 8.10 (d, *J* = 9.0 Hz, 3H), 7.14 (d, *J* = 9.0 Hz, 1H), 4.87 (s, 1H), 4.67 (t, *J* = 9.0 Hz, 1H), 3.65–3.57 (m, 1H), 3.40 (dd, *J* = 11.8, 4.9 Hz, 2H), 3.16 (t, *J* = 8.6 Hz, 1H), 3.11–2.99 (m, 2H), 2.35 (t, *J* = 7.1 Hz, 2H), 2.10 (ddt, *J* = 30.3, 15.2, 7.5 Hz, 2H), 1.76–1.60 (m, 2H). ^13^C-NMR (151 MHz, DMSO-*d_6_*) *δ* 172.68 (s), 164.31 (s), 163.86 (s), 149.25 (s), 134.91 (s), 130.11 (s), 128.51 (s), 119.73 (s), 117.40 (s), 79.81 (s), 78.80 (s), 77.61 (s), 72.57 (s), 70.22 (s), 61.20 (s), 35.44 (s), 34.49 (s), 20.00 (s); HRMS (ESI): Calcd. for [M − H]^−^ C_20_H_24_N_7_O_12_S_2_: 618.1003, Found 618.0984.

##### *N*^1^-(2-Methyl-4-((5-sulfamoyl-1,3,4-thiadiazol-2-yl)carbamoyl)phenyl)-*N*^5^-((2*R*,3*R*,4*S*,5*S*,6*R*)-3,4,5-trihydroxy-6-(hydroxymethyl)tetrahydro-2*H*-pyran-2-yl)glutaramide (**14g**)

Two-step yield 10.4%, ^1^H-NMR (600 MHz, DMSO-*d_6_*) *δ* 13.21 (s, 1H), 9.37 (s, 1H), 8.35 (d, *J* = 9.1 Hz, 1H), 8.15 (s, 2H), 8.03 (d, *J* = 1.3 Hz, 1H), 7.96 (dd, *J* = 8.4, 1.6 Hz, 1H), 7.70 (dd, *J* = 23.7, 8.3 Hz, 1H), 4.97 (d, *J* = 0.5 Hz, 1H), 4.87 (dd, *J* = 11.1, 4.8 Hz, 2H), 4.73 (t, *J* = 9.1 Hz, 1H), 4.49 (t, *J* = 5.1 Hz, 1H), 3.64 (dd, *J* = 11.5, 2.4 Hz, 1H), 3.45–3.38 (m, 1H), 3.18 (t, *J* = 8.4 Hz, 1H), 3.13–2.98 (m, 3H), 2.43 (t, *J* = 7.1 Hz, 2H), 2.30 (s, 3H), 2.27–2.11 (m, 2H), 1.84 (p, *J* = 7.3 Hz, 2H). ^13^C-NMR (151 MHz, DMSO-*d_6_*) *δ* 172.91, 171.77, 167.19, 165.52, 163.65, 140.71, 131.17, 130.78, 126.93, 126.07, 123.97, 79.80, 78.86, 77.71, 72.70, 70.30, 61.29, 35.71, 35.03, 21.43, 18.41; HRMS (ESI): Calcd. for [M − H]^−^ C_21_H_27_N_6_O_10_S_2_: 587.1308, Found 587.1293.

##### *N*^1^-(2-Methoxy-4-((5-sulfamoyl-1,3,4-thiadiazol-2-yl)carbamoyl)phenyl)-*N*^5^-((2*R*,3*R*,4*S*,5*S*,6*R*)-3,4,5-trihydroxy-6-(hydroxymethyl)tetrahydro-2*H*-pyran-2-yl)glutaramide (**14h**)

Two-step yield 11.9%, ^1^H-NMR (600 MHz, DMSO-*d_6_*) *δ* 13.44 (s, 1H), 9.38 (s, 1H), 8.36 (s, 2H), 8.34 (d, *J* = 9.1 Hz, 1H), 8.30 (d, *J* = 8.4 Hz, 1H), 7.85 (d, *J* = 1.8 Hz, 1H), 7.78 (dd, *J* = 8.5, 1.8 Hz, 1H), 4.90 (s, 1H), 4.72 (t, *J* = 9.1 Hz, 1H), 3.97 (s, 3H), 3.64 (dd, *J* = 11.7, 1.7 Hz, 1H), 3.41 (dd, *J* = 11.8, 5.4 Hz, 1H), 3.18 (t, *J* = 8.8 Hz, 1H), 3.14–2.95 (m, 3H), 2.54–2.51 (m, 2H), 2.29–2.01 (m, 2H), 1.91–1.71 (m, 2H). ^13^C-NMR (151 MHz, DMSO-*d_6_*) *δ* 172.88, 172.33, 165.03, 162.63, 148.93, 132.83, 125.69, 122.29, 120.46, 111.13, 79.81, 78.87, 77.73, 72.71, 70.32, 61.30, 56.59, 36.05, 34.99, 21.31; HRMS (ESI): Calcd. for [M − H]^−^ C_21_H_27_N_6_O_11_S_2_: 603.1257, Found 603.1232.

##### *N*^1^-(4-((5-Sulfamoyl-1,3,4-thiadiazol-2-yl)carbamoyl)phenyl)-*N*^4^-((2*R*,3*R*,4*S*,5*S*,6*R*)-3,4,5-trihydroxy-6-(hydroxymethyl)tetrahydro-2*H*-pyran-2-yl)succinimide (**14i**)

Two-step yield 9.9%, ^1^H-NMR (600 MHz, DMSO-*d_6_*) *δ* 13.36 (s, 1H), 10.39 (s, 1H), 8.44 (d, *J* = 9.1 Hz, 1H), 8.35 (s, 2H), 8.13 (d, *J* = 8.7 Hz, 2H), 7.78 (d, *J* = 8.7 Hz, 2H), 4.87 (s, 1H), 4.71 (t, *J* = 9.1 Hz, 1H), 3.67–3.59 (m, 1H), 3.41 (dd, *J* = 11.8, 5.1 Hz, 1H), 3.35 (s, 1H), 3.17 (t, *J* = 8.6 Hz, 1H), 3.11–3.01 (m, 3H), 2.65–2.59 (m, 2H), 2.56–2.51 (m, 2H). ^13^C-NMR (151 MHz, DMSO-*d_6_*) *δ* 174.26, 172.21, 172.13, 171.89, 171.38, 142.90, 131.11, 129.90, 118.41, 79.98, 78.91, 77.74, 72.84, 70.26, 61.21, 31.74, 30.59; HRMS (ESI): Calcd. for [M − H]^−^ C_19_H_23_N_6_O_10_S_2_: 559.0995, Found 559.0921.

##### *N*^1^-(3-((5-Sulfamoyl-1,3,4-thiadiazol-2-yl)carbamoyl)phenyl)-*N*^4^-((2*R*,3*R*,4*S*,5*S*,6*R*)-3,4,5-trihydroxy-6-(hydroxymethyl)tetrahydro-2*H*-pyran-2-yl)succinimide (**14j**)

Two-step yield 9.0%, ^1^H-NMR (600 MHz, DMSO-*d_6_*) *δ* 13.55 (s, 1H), 10.24 (s, 1H), 8.44 (d, *J* = 9.1 Hz, 1H), 8.35 (d, *J* = 18.7 Hz, 3H), 7.94–7.74 (m, 2H), 7.49 (t, *J* = 7.9 Hz, 1H), 4.98 (d, *J* = 4.5 Hz, 1H), 4.87 (dd, *J* = 11.3, 5.2 Hz, 2H), 4.71 (t, *J* = 9.1 Hz, 1H), 4.51 (t, *J* = 5.7 Hz, 1H), 3.63 (dd, *J* = 11.5, 4.0 Hz, 1H), 3.48–3.37 (m, 1H), 3.17 (td, *J* = 8.6, 4.4 Hz, 1H), 3.12–2.99 (m, 3H), 2.66–2.56 (m, 2H), 2.50–2.44 (m, 2H). ^13^C-NMR (151 MHz, DMSO-*d_6_*) *δ* 172.22, 171.32, 166.16, 165.06, 162.59, 140.06, 131.96, 129.65, 124.00, 123.20, 119.52, 79.97, 78.91, 77.73, 72.82, 70.26, 61.22, 31.58, 30.56; HRMS (ESI): Calcd. for [M − H]^−^ C_19_H_23_N_6_O_10_S_2_: 559.0995, Found 559.0979.

##### *N*^1^-(2-Fluoro-4-((5-sulfamoyl-1,3,4-thiadiazol-2-yl)carbamoyl)phenyl)-*N*^4^-((2*R*,3*R*,4*S*,5*S*,6*R*)-3,4,5-trihydroxy-6-(hydroxymethyl)tetrahydro-2*H*-pyran-2-yl)succinimide (**14k**)

Two-step yield 8.5%, ^1^H-NMR (600 MHz, DMSO-*d_6_*) *δ* 9.95 (s, 1H), 8.43 (d, *J* = 9.1 Hz, 1H), 8.12 (t, *J* = 7.9 Hz, 1H), 7.99–7.88 (m, 4H), 4.97 (s, 1H), 4.86 (dd, *J* = 9.3, 5.2 Hz, 3H), 4.75–4.65 (m, 2H), 4.51 (t, *J* = 6.0 Hz, 2H), 3.63 (dd, *J* = 10.3, 5.0 Hz, 2H), 3.45–3.38 (m, 2H), 3.21–3.13 (m, 2H), 3.11–2.99 (m, 5H), 2.72–2.62 (m, 2H), 2.46–2.32 (m, 2H).

##### *N*^1^-(2-Chloro-4-((5-sulfamoyl-1,3,4-thiadiazol-2-yl)carbamoyl)phenyl)-*N*^4^-((2*R*,3*R*,4*S*,5*S*,6*R*)-3,4,5-trihydroxy-6-(hydroxymethyl)tetrahydro-2*H*-pyran-2-yl)succinimide (**14l**)

Two-step yield 7.6%, ^1^H-NMR (600 MHz, DMSO-*d_6_*) *δ* 13.26 (s, 1H), 8.42 (d, *J* = 9.0 Hz, 1H), 8.14 (d, *J* = 2.0 Hz, 1H), 7.88 (dd, *J* = 8.6, 2.0 Hz, 1H), 6.87 (d, *J* = 8.6 Hz, 1H), 6.43 (s, 2H), 4.65 (t, *J* = 9.0 Hz, 1H), 3.60 (d, *J* = 11.3 Hz, 1H), 3.40 (dd, *J* = 11.8, 4.5 Hz, 1H), 3.25–3.10 (m, 1H), 3.03 (dd, *J* = 16.3, 7.9 Hz, 3H), 2.61–2.53 (m, 2H), 2.44–2.27 (m, 2H). ^13^C-NMR (151 MHz, DMSO-*d_6_*) *δ* 172.22, 171.62, 164.47, 164.04, 161.07, 150.01, 130.64, 129.55, 117.99, 116.64, 114.65, 79.87, 78.77, 77.58, 72.69, 70.14, 61.10, 30.92, 29.27; HRMS (ESI): Calcd. for [M − H]^−^ C_19_H_22_ClN_6_O_10_S_2_: 593.0606, Found 577.0600.

##### *N*^1^-(2-Bromo-4-((5-sulfamoyl-1,3,4-thiadiazol-2-yl)carbamoyl)phenyl)-*N*^4^-((2*R*,3*R*,4*S*,5*S*,6*R*)-3,4,5-trihydroxy-6-(hydroxymethyl)tetrahydro-2*H*-pyran-2-yl)succinimide (**14m**)

Two-step yield 6.6%, ^1^H-NMR (600 MHz, DMSO-*d_6_*) *δ* 13.25 (s, 1H), 8.39 (d, *J* = 9.1 Hz, 1H), 8.29 (d, *J* = 2.0 Hz, 1H), 7.90 (dd, *J* = 8.6, 2.0 Hz, 1H), 6.85 (d, *J* = 8.6 Hz, 1H), 6.37 (s, 2H), 4.66 (t, *J* = 9.1 Hz, 1H), 3.61 (d, *J* = 10.5 Hz, 1H), 3.39 (dd, *J* = 11.8, 4.9 Hz, 2H), 3.15 (dd, *J* = 15.2, 6.6 Hz, 1H), 3.10–2.95 (m, 3H), 2.56–2.51 (m, 2H), 2.35 (td, *J* = 16.8, 9.6 Hz, 2H). ^13^C-NMR (151 MHz, DMSO-*d_6_*) *δ* 172.29, 171.61, 164.30, 164.01, 161.17, 151.06, 133.90, 130.05, 118.50, 114.53, 106.46, 79.86, 78.78, 77.61, 72.71, 70.16, 61.11, 30.97, 29.30; HRMS (ESI): Calcd. for [M − H]^−^ C_19_H_22_BrN_6_O_10_S_2_: 637.0100, Found 637.0061.

##### *N*^1^-(2-Nitro-4-((5-sulfamoyl-1,3,4-thiadiazol-2-yl)carbamoyl)phenyl)-*N*^4^-((2*R*,3*R*,4*S*,5*S*,6*R*)-3,4,5-trihydroxy-6-(hydroxymethyl)tetrahydro-2*H*-pyran-2-yl)succinimide (**14n**)

Two-step yield 8.1%, ^1^H-NMR (600 MHz, DMSO-*d_6_*) *δ* 13.64 (s, 1H), 9.00 (d, *J* = 2.1 Hz, 1H), 8.41 (d, *J* = 9.0 Hz, 1H), 8.09 (dd, *J* = 8.9, 2.2 Hz, 3H), 7.13 (d, *J* = 9.0 Hz, 1H), 4.65 (t, *J* = 9.0 Hz, 1H), 3.60 (d, *J* = 10.5 Hz, 2H), 3.39 (dd, *J* = 11.8, 4.7 Hz, 2H), 3.21–3.11 (m, 1H), 3.11–2.97 (m, 3H), 2.58–2.51 (m, 3H), 2.43–2.28 (m, 2H). ^13^C-NMR (151 MHz, DMSO-*d_6_*) *δ* 172.29, 171.53, 164.30, 163.98, 161.29, 149.24, 134.95, 130.13, 128.51, 119.71, 117.39, 79.88, 78.82, 77.64, 72.73, 70.17, 61.12, 30.99, 29.32; HRMS (ESI): Calcd. for [M − H]^−^ C_19_H_22_N_7_O_12_S_2_: 604.0846, Found 604.0834.

##### *N*^1^-(2-Methy-4-((5-sulfamoyl-1,3,4-thiadiazol-2-yl)carbamoyl)phenyl)-*N*^4^-((2*R*,3*R*,4*S*,5*S*,6*R*)-3,4,5-trihydroxy-6-(hydroxymethyl)tetrahydro-2*H*-pyran-2-yl)succinimide (**14o**)

Two-step yield 10.2%, ^1^H-NMR (600 MHz, DMSO-*d_6_*) *δ* 13.36 (s, 1H), 9.50 (s, 1H), 8.45 (d, J = 9.0 Hz, 1H), 8.36 (s, 1H), 8.04 (s, 1H), 7.91 (dd, J = 83.8, 8.5 Hz, 2H), 4.72 (t, J = 9.0 Hz, 1H), 3.64 (d, J = 11.0 Hz, 1H), 3.42 (dd, J = 11.7, 5.1 Hz, 1H), 3.22–3.15 (m, 3H), 3.12–3.02 (m, 4H), 2.75 (dd, J = 11.7, 4.4 Hz, 2H), 2.68–2.61 (m, 2H), 2.33 (s, 3H). ^13^C-NMR (151 MHz, DMSO-*d_6_*) *δ* 172.46, 171.49, 165.56, 164.88, 163.45, 162.71, 141.64, 131.29, 130.59, 127.10, 123.54, 79.95, 78.85, 77.66, 72.80, 70.24, 61.22, 31.39, 30.81, 18.35; HRMS (ESI): Calcd. for [M − H]^−^ C_20_H_25_N_6_O_10_S_2_: 573.1152, Found 573.1126.

##### *N*^1^-(2-Methoxy-4-((5-sulfamoyl-1,3,4-thiadiazol-2-yl)carbamoyl)phenyl)-*N*^4^-((2*R*,3*R*,4*S*,5*S*,6*R*)-3,4,5-trihydroxy-6-(hydroxymethyl)tetrahydro-2*H*-pyran-2-yl)succinimide (**14p**)

Two-step yield 10.2%, ^1^H-NMR (600 MHz, DMSO-*d_6_*) *δ* 13.43 (s, 1H), 9.48 (s, 1H), 8.42 (d, *J* = 9.1 Hz, 1H), 8.36 (s, 2H), 8.30 (d, *J* = 8.3 Hz, 1H), 7.85 (s, 1H), 7.78 (d, *J* = 9.9 Hz, 1H), 4.85 (s, 1H), 4.71 (t, *J* = 9.1 Hz, 1H), 3.97 (s, 3H), 3.63 (d, *J* = 10.5 Hz, 1H), 3.60 (s, 1H), 3.41 (dd, *J* = 11.8, 5.1 Hz, 2H), 3.17 (t, *J* = 8.7 Hz, 1H), 3.12–3.01 (m, 3H), 2.77 (t, *J* = 6.6 Hz, 1H), 2.75–2.64 (m, 2H), 2.49–2.41 (m, 2H). ^13^C-NMR (151 MHz, DMSO-*d_6_*) *δ* 172.14, 171.94, 165.16, 162.75, 148.81, 133.08, 132.92, 122.37, 120.28, 111.19, 80.04, 79.02, 78.00, 73.02, 70.44, 61.36, 56.61, 31.84, 30.75; HRMS (ESI): Calcd. for [M − H]^−^ C_20_H_25_N_6_O_11_S_2_: 589.1101, Found 589.1077.

##### 3.2. hCA Enzyme Inhibition Assays

Carbonic anhydrase can catalyze the hydrolysis of 4-nitrophenyl acetate (4-NPA). According to the method reported in the literature, the reaction rate was monitored by measuring the change of absorbance at 405 nM [8,29,30]. The experimental buffer consisted of 15 mmol/L 4-(2-hydroxyethyl)-1-piperazineethanesulfonic acid (HEPES) (pH = 7.40), 0.01% tetraethylene glycol monododecyl ether (Brij), and 100 mmol/L NaCl. Recombinant human carbonic anhydrase II, IX and XII were commercially available (Sino Biological Inc, Beijing, China) and prepared in buffer with a concentration of 3.3, 10.0, and 10.0 ng/μL, respectively. Then, 18 μL of enzyme solution was transferred into 384-well plates in duplicate. Stock solutions of the inhibitors (10.0 mmol/L) were prepared using DMSO as solvent, and then diluted 1:3 with DMSO. The test concentration included 600, 200, 66.667, 22.222, 7.407, 2.469, 0.823, 0.274, 0.091 μM, a total of nine concentrations. Then 2.00 μL of each inhibitor was added to each well. All compounds were allowed to incubate with the enzyme for 15 min at 25 °C to form the Enzyme-Inhibitor (E-I) complex. After that, substrate 4-NPA (1.00 mmol/L, 20.00 μL, Sigma-Aldrich, St. Louis, MI, USA) was added into the E-I complex solution and incubated for 60, 90, 90 min at 25 °C, respectively. The absorbance of each compound was measured with Envision 2104 plate reader (Perkin Elmer, Waltham, MA, USA). The inhibitor AZM was used as standard to investigate the inhibitory activity of these compounds.

### 3.3. Preparation for Docking Studies and Prediction by TPSA

Molecular docking calculations using the ligand molecules with CA IX (PDB code: 5FL4) [31] and were conducted with the modified version of Autodock (version Autodock 4(Zn), Scripps Research Institute, San Diego, CA, USA) [32,33]. A grid box with a grid spacing of 0.375 Å was generated to define the binding pocket. 

AutoGrid 4 was used as the auxiliary program to generate affinity grid fields. Compound structures were built and minimized with the Accelrys Discovery Studio 3.0 software package with flexible torsions assigned, and all dihedral angles were able to freely rotate. We applied the Lamarckian genetic algorithm to determine the appropriate binding positions, orientations, and conformation of ligands. The optimized parameters were as follows: the maximum number of energy evaluations was increased to 25,000,000 per run, the iterations of Solis & Wets local search were 3000, the number of individuals in the population was 300, and the number of generations was 25. Results differing by < 2 Å in a positional root mean square deviation were clustered together. In each group, the lowest binding energy configuration with the highest percentage frequency was selected as the group representative. All other parameters were maintained as default.

ADME/T profile of the selected compounds was assessed using the standard descriptors protocol in the DS (version 3.0, Accelrys Inc., San Diego, CA, USA) [34]. In the ADME/T module, various parameters including TPSA were used to quantitatively predict the properties of a set of rules that specify the ADME/T characteristics of the compounds.

### 3.4. Preparation for Molecular Dynamics Simulation

The 100 ns molecular dynamics (MD) simulations were carried out of CA IX-compound 14a, CA IX-compound 14e, and CA IX-compound 14i, respectively. The simulations were performed by Desmond v3.8 module in the Schrödinger suite (version 9.6, Schrödinger Inc., NY, USA). The system was solvated with simple point charge (SPC) water and neutralized by adding an appropriate amount of counter ions in an orthorhombic box (10 Å × 10 Å × 10 Å) to form a buffer area between the protein atoms and the side of the box. Then, the Optimized Potentials for Liquid Simulation (OPLS_2005) force field was used to minimize the energy of the complex system, and the maximum number of iterations in the minimization process was set to 5000, and the convergence threshold was kept at 1.0 kcal/mol/Å. A mixed method of the steepest descent and the limited memory Broyden-Fletcher-Goldfarb-Shanno (LBFGS) algorithms with the maximum of 5000 steps were performed to minimize the system energy until a gradient threshold of 25 kcal/mol/Å was reached. Before MD simulations, a 100 ps simulation was performed to relax the whole system. The temperature was set to 300 K and maintained throughout by implementing Nose-Hoover thermostat with the pressure set to 1.01325 bar and maintained through Martyna-Tobias-Klein pressure bath. Finally, the 100 ns MD simulations were carried out, a time interval of every trajectory was recorded at every 100 ps.

### 3.5. CCK-8 Assay In Vitro

The MDA-MB-231, HT-29 and MG-63 cell lines were provided by KeyGEN BioTECH Ltd (Nanjing, Jiangsu, China). The cells were digested and counted to prepare a cell suspension of 5.0 × 10^4^ cells/mL, and then 100 μL of cell suspension was added to each well of the 96-well cell culture plate. The plates were placed in a 37 °C, 5% CO_2_ incubator (Corning Incorporated, Corning, New York, USA) for 24 h. The drug was diluted with the culture medium to the required concentration, ed100 μL of the corresponding drug-containing medium was added to each well, and a negative control group was set up at the same time. The 96-well cell culture plates were placed into a normoxic incubator (37 °C, 5% CO_2_) and hypoxic incubator (37 °C, 5% CO_2_, 0.5% O_2_, 94.5% N_2_) for 48 h. Finally, the 96-well plate was stained with CCK-8 and the OD value determined (λ = 450 nm).

### 3.6. Measurement of Extracellular pH

The measurement of the change of extracellular pH referred to the reported literature [26]. In brief, cancer cells were incubated for 12 h. After removing the medium, fresh medium was added to the plates and then incubated for 48 h under hypoxia or normoxia. The pH of the medium was measured immediately. The tested compounds were dissolved in DMSO and diluted with culture medium to the desired concentration. After the addition of tested compounds, the same procedure was repeated.

## 4. Conclusions

Herein, a series of saccharide-modified thiadiazole sulfonamide derivatives were reported. Intermediates **12a–h** and compounds **14a–p** were assayed for their inhibitory activities against three *h*CA isoforms of pharmacological relevance: tumor-associated transmembrane isoforms *h*CA IX and XII and cytosolic off-target isoform *h*CA II. The results indicated that all the tested compounds showed activity against *h*CA II, IX, and XII. Among them, the IC_50_ values for the saccharide-modified derivatives with high TPSA values for *h*CA II and IX ranged from 80.3–1403.1 nM and 29.0–1082.0 nM, respectively. Molecular docking results showed that the interaction with residue Gln92 and the distance between sulfanilamide and Zn^2+^ had important impacts on the activity. Further cell experiments showed that compounds **14a**, **14b**, **14h**, and **14p** had inhibitory effects on the viability of cancer cell lines with high expression of CA IX, and this viability inhibition was enhanced under hypoxic conditions. Compound **14a** had better inhibitory activity than the positive control AZM on MG-63 cells with high expression of CA IX and XII. In addition, compounds **14b**, **14h**, and **14p** significantly modified the acidic microenvironments of cancer cells. Based on the above results and the known importance of the acidic microenvironment for tumor growth, we believe that saccharide-modified CA IX inhibitors have prospects for further development as combination drugs for tumor treatment.

## Figures and Tables

**Table 1:** The IC_50_ against relevant *h*CA isoforms and the topological polar surface area (TPSA) of the intermediates **12a–h** and compounds **14a–p** (Comp. **12a–h** and **14a–p**).

Comp.	R	*h*CA II (IC_50_, nM)	*h*CA IX (IC_50_, nM)	*h*CA XII (IC_50_, nM)	TPSA (Å^2^)
**12a**	R = H	109.9 ± 6.9	44.6 ± 8.3	n.t.	140.3
**12b**	-	79.1 ± 5.4	63.8 ± 6.3	n.t.	140.3
**12c**	R = 3-F	242.4 ± 9.4	41.3 ± 6.2	n.t.	140.3
**12d**	R = 3-Cl	401.1 ± 12.3	36.5 ± 7.6	n.t.	140.3
**12e**	R = 3-Br	543.2 ± 10.3	65.9 ± 8.3	n.t.	140.3
**12f**	R = 3-NO_2_	701.3 ± 13.2	55.1 ± 11.5	n.t.	183.1
**12g**	R = 3-CH_3_	119.7 ± 14.8	43.8 ± 6.6	n.t.	140.3
**12h**	R = 3-OCH_3_	147.2 ± 15.3	36.4 ± 8.3	n.t.	149.2
**14a**	n = 3, R = H	140.9 ± 12.9	29.0 ± 4.2	140.8 ± 5.5	266.2
**14b**	n = 3	80.3 ± 11.5	29.0 ± 1.5	56.0 ± 8.5	266.2
**14c**	n = 3, R = 3-F	215.0 ± 9.5	76.6 ± 6.2	81.8 ± 11.1	266.2
**14d**	n = 3, R = 3-Cl	765.4 ± 15.1	279.8 ± 10.2	n.t.	266.2
**14e**	n = 3, R = 3-Br	885.6 ± 13.7	1082.0 ± 8.5	n.t.	266.2
**14f**	n = 3, R = 3-NO_2_	995.3 ± 10.6	117.1 ± 6.9	n.t.	309.0
**14g**	n = 3, R = 3-CH_3_	165.2 ± 9.1	53.4 ± 6.2	70.5 ± 10.9	266.2
**14h**	n = 3, R = 3-OCH_3_	271.7 ± 9.0	68.8 ± 10.2	84.9 ± 7.6	275.1
**14i**	n = 2, R = H	197.4 ± 7.9	54.9 ± 6.2	52.8 ± 11.4	266.2
**14j**	n = 2	260.3 ± 12.6	69.4 ± 5.7	40.6 ± 6.9	266.2
**14k**	n = 2, R = 3-F	474.4 ± 7.1	141.1 ± 8.1	n.t.	266.2
**14l**	n = 2, R = 3-Cl	612.0 ± 8.5	255.9 ± 7.9	n.t.	266.2
**14m**	n = 2, R = 3-Br	823.7 ± 11.1	865.0 ± 10.7	n.t.	266.2
**14n**	n = 2, R = 3-NO_2_	1403.1 ± 24.6	290.9 ± 10.9	n.t.	309.0
**14o**	n = 2, R = 3-CH_3_	269.8 ± 13.9	135.0 ± 7.0	n.t.	266.2
**14p**	n = 2, R = 3-OCH_3_	332.1 ± 16.8	47.9 ± 7.1	42.3 ± 9.4	275.1
AZM		16.7 ± 4.7	50.2 ± 12.8	38.2 ± 10.3	113.8

## Data Availability

Not applicable.

## Supplementary Materials

The following are available online at https://www.mdpi.com/article/10.3390/ijms22115482/s1.

## References

[1] Supuran C.T. (2020). Experimental carbonic anhydrase inhibitors for the treatment of hypoxic tumors. J. Exp. Pharmacol..

[2] Angeli A., Carta F., Nocentini A., Winum J.Y., Zalubovskis R., Akdemir A., Onnis V., Eldehna W.M., Capasso C., De Simone G. (2020). Carbonic Anhydrase Inhibitors Targeting Metabolism and Tumor Microenvironment. Metabolites.

[3] Winum J.Y., Colinas P. (2015). Chapter 21-Carbonic Anhydrases as Esterases and Their Biotechnological Applications. CARBONIC Anhydrases as Biocatalysts.

[4] Akgul O., Mannelli L.C., Vullo D., Angeli A., Ghelardini C., Bartolucci G., Altamimi A.S.A., Scozzafava A., Supuran C.T., Carta F. (2018). Discovery of novel nonsteroidal anti-inflammatory drugs and carbonic anhydrase inhibitors hybrids (NSAIDs-CAIs) for the management of rheumatoid arthritis. J. Med. Chem..

[5] Andring J.T., Fouch M., Akocak S., Angeli A., Supuran C.T., Ilies M.A., McKenna R. (2020). Structural basis of nanomolar inhibition of tumor-associated carbonic anhydrase IX: X-ray crystallographic and inhibition study of lipophilic inhibitors with acetazolamide backbone. J. Med. Chem..

[6] Alterio V., Di Fiore A., D’Ambrosio K., Supuran C.T., De Simone G. (2012). Multiple binding modes of inhibitors to carbonic anhydrases: How to design specific drugs targeting 15 different isoforms?. Chem. Rev..

[7] Ali M., Bozdag M., Farooq U., Angeli A., Carta F., Berto P., Zanotti G., Supuran C.T. (2020). Benzylaminoethyureido-tailed benzenesulfonamides: Design, synthesis, kinetic and X-ray Investigations on human carbonic anhydrases. Int. J. Mol. Sci..

[8] Zhang Z.P., Yin Z.F., Li J.Y., Wang Z.P., Wu Q.J., Wang J., Liu Y., Cheng M.S. (2019). Synthesis, molecular docking analysis, and carbonic anhydrase inhibitory evaluations of benzenesulfonamide derivatives containing thiazolidinone. Molecules.

[9] Mam Y.M., Brian P.M., Robert M., Susan C.F. (2018). Carbonic anhydrases: Role in pH control and cancer. Metabolites.

[10] Supuran C.T. (2017). Carbonic anhydrase inhibition and the management of hypoxic tumors. Metabolites.

[11] Lee S.H., McIntyre D., Honess D., Hulikova A., Pacheco-Torres J., Cerdán S., Swietach P., Harris A.L., Griffiths J.R. (2018). Carbonic anhydrase IX is a pH-stat that sets an acidic tumour extracellular pH in vivo. Br. J. Cancer.

[12] Galati S., Yonchev D., Rodríguez-Pérez R., Vogt M., Tuccinardi T., Bajorath J. (2021). Predicting isoform-selective carbonic anhydrase inhibitors via machine learning and rationalizing structural features important for selectivity. ACS Omega.

[13] Strapcova S., Takacova M., Csaderova L., Martinelli P., Lukacikova L., Gal V., Kopacek J., Svastova E. (2020). Clinical and pre-clinical evidence of carbonic anhydrase IX in pancreatic cancer and its high expression in pre-cancerous lesions. Cancers.

[14] A Study of SLC-0111 and Gemcitabine for Metastatic Pancreatic Ductal Cancer in Subjects Positive for CAIX. https://clinicaltrials.gov/ct2/show/NCT03450018.

[15] Pacchiano F., Carta F., McDonald P.C., Lou Y., Vullo D., Scozzafava A., Dedhar S., Supuran C.T. (2011). Ureido-substituted benzenesulfonamides potently inhibit carbonic anhydrase IX and show antimetastatic activity in a model of breast cancer metastasis. J. Med. Chem..

[16] Bonardi A., Nocentini A., Bua S., Combs J., Lomelino C., Andring J., Lucarini L., Sgambellone S., Masini E., McKenna R. (2020). Sulfonamide inhibitors of human carbonic anhydrases designed through a three-tails approach: Improving ligand/isoform matching and selectivity of action. J. Med. Chem..

[17] Supuran C.T. (2012). Structure-based drug discovery of carbonic anhydrase inhibitors. J. Enzym. Inhib. Med. Chem..

[18] Nakamura S., Yamashita M., Yokota D., Hirano I., Ono T., Fujie M., Shibata K., Niimi T., Suyama T., Maddali K. (2010). Development and pharmacologic characterization of deoxybromophospha sugar derivatives with antileukemic activity. Investig. New Drugs.

[19] Khalaf H.S., Tolan H.E.M., El-Bayaa M.N., Radwan M.A.A., El-Manawaty M., El-Sayed W.A. (2020). Synthesis and anticancer activity of new pyridine-thiophene and pyridine-furan hybrid compounds, their sugar hydrazone, and glycosyl derivatives. Russ. J. Gen. Chem..

[20] Nassar I.F., El-Farargy A.F., Abdelrazek F.M., Hamza Z. (2020). Synthesis of new uracil derivatives and their sugar hydrazones with potent antimicrobial, antioxidant and anticancer activities. Nucleos. Nucleot. Nucl..

[21] D’Andrea F., Sartini S., Piano I., Franceschi M., Quattrini L., Guazzelli L., Ciccone L., Orlandini E., Gargini C., Motta C.L. (2020). Oxy-imino saccharidic derivatives as a new structural class of aldose reductase inhibitors endowed with anti-oxidant activity. J. Enzym. Inhib. Med. Chem..

[22] Chen X., Gu H., Lyu Z., Liu X., Wang L., Chen H., Brash J.L. (2018). Sulfonate groups and saccharides as essential structural elements in heparin-mimicking polymers used as surface modifiers: Optimization of relative contents for anti-thrombogenic properties. ACS Appl. Mater. Interfaces.

[23] Guler O.O., De Sımone G., Supuran C.T. (2010). Drug design studies of the novel antitumor targets carbonic anhydrase IX and XII. Curr. Med. Chem..

[24] Mishra C.B., Tiwari M., Supuran C.T. (2020). Progress in the development of human carbonic anhydrase inhibitors and their pharmacological applications: Where are we today?. Med. Res. Rev..

[25] Hou Z., Lin B., Bao Y., Yan H.N., Zhang M., Chang X.W., Zhang X.X., Wang Z.J., Wei G.F., Cheng M.S. (2017). Dual-tail approach to discovery of novel carbonic anhydrase IX inhibitors by simultaneously matching the hydrophobic and hydrophilic halves of the active site. Eur. J. Med. Chem..

[26] Nocentini A., Trallori E., Singh S., Lomelino C.L., Bartolucci G., Di C.M.L., Ghelardini C., Mckenna R., Gratteri P., Supuran C.T. (2018). 4-Hydroxy-3-nitro-5-ureido-benzenesulfonamides selectively target the tumor-associated carbonic anhydrase isoforms IX and XII showing hypoxia-enhanced antiproliferative profiles. J. Med. Chem..

[27] Akocak S., Güzel-Akdemir Ö., Sanku R.K.K., Russom S.S., Iorga B.I., Supuran C.T., Ilies M.A. (2020). Pyridinium derivatives of 3-aminobenzenesulfonamide are nanomolarpotent inhibitors of tumor-expressed carbonic anhydrase isozymes CA IX and CA XII. Bioorg. Chem..

[28] Kumar D., Mishra K.B., Mishra B.B., Mondal S., Tiwari V.K. (2014). Click chemistry inspired highly facile synthesis of triazolyl ethisterone glycoconjugates. Steroids.

[29] Li F.R., Fan Z.F., Qi S.J., Wang Y.S., Wang J., Liu Y., Cheng M.S. (2017). Design, synthesis, molecular docking analysis, and carbonic anhydrase IX inhibitory evaluations of novel N-Substituted-β-d-glucosamine derivatives that incorporate benzenesulfonamides. Molecules.

[30] Verpoorte J.A., Mehta S., Edsall J.T. (1967). Esterase activities of human carbonic anhydrases B and C. J. Boil. Chem..

[31] Leitans J., Kazaks A., Balode A., Ivanova J., Zalubovskis R., Supuran C.T., Tars K. (2015). Efficient expression and crystallization system of cancer-associated carbonic anhydrase isoform IX. J. Med. Chem..

[32] Morris G.M., Huey R., Lindstrom W., Sanner M.F., Belew R.K., Goodsell D.S., Olson A.J. (2009). AutoDock4 and AutoDockTools4: Automated docking with selective receptor flexibility. J. Comput. Chem..

[33] Diogo S.M., Stefano F., Maria J.R., Arthur J.O. (2014). AutoDock4Zn: An improved autodock force field for small-molecule docking to zinc metalloproteins. J. Chem. Inf. Model..

[34] (2008). Discovery Studio User Manual.

