# Supplementary material for: Synthesis, Molecular Docking Analysis and Biological Evaluations of Saccharide-Modified Thiadiazole Sulfonamide Derivatives
Source: Int J Mol Sci. 2021 May 22;22(11):5482. doi: 10.3390/ijms22115482 (PMC8196973; doi:10.3390/ijms22115482)
Supplement: Supplementary file 1 [file ijms-22-05482-s001.zip › ijms-1200458-supplementary.pdf]

Supporting Information

Synthesis, Molecular Docking Analysis and Biological Evaluations of Saccharide-modified Thiadiazole Sulfonamide Derivatives

Zuo-Peng Zhang, Ye Zhong, Zhen-Bin Han, Lin Zhou, Hua-Sheng Su, Jian Wang, Yang Liu*, Mao-Sheng Cheng*

Table of Contents

^{1}H-NMR and ^{13}C-NMR Spectra........2

2D-NMR Spectra........29

Modes of binding figures........32

Supplementary data........33

^{1}H-NMR and ^{13}C-NMR Spectra

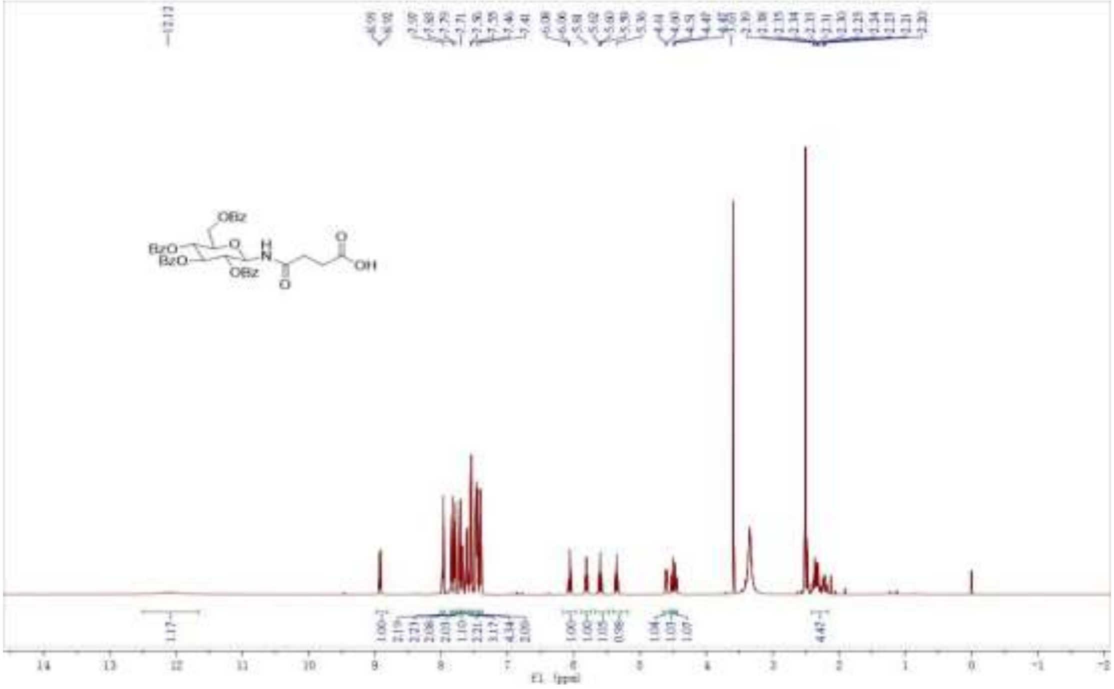

Figure S1. The ^{1}H-NMR of intermediate 5a.

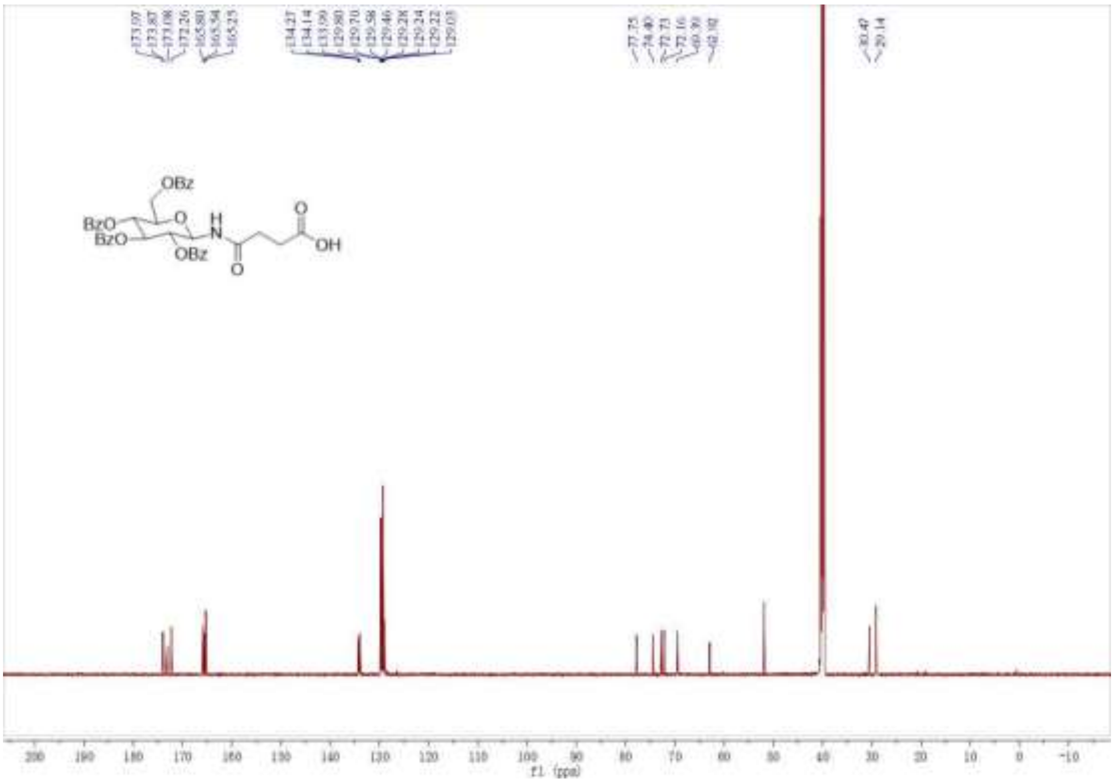

Figure S2. The ^{13}C-NMR of intermediate 5a.

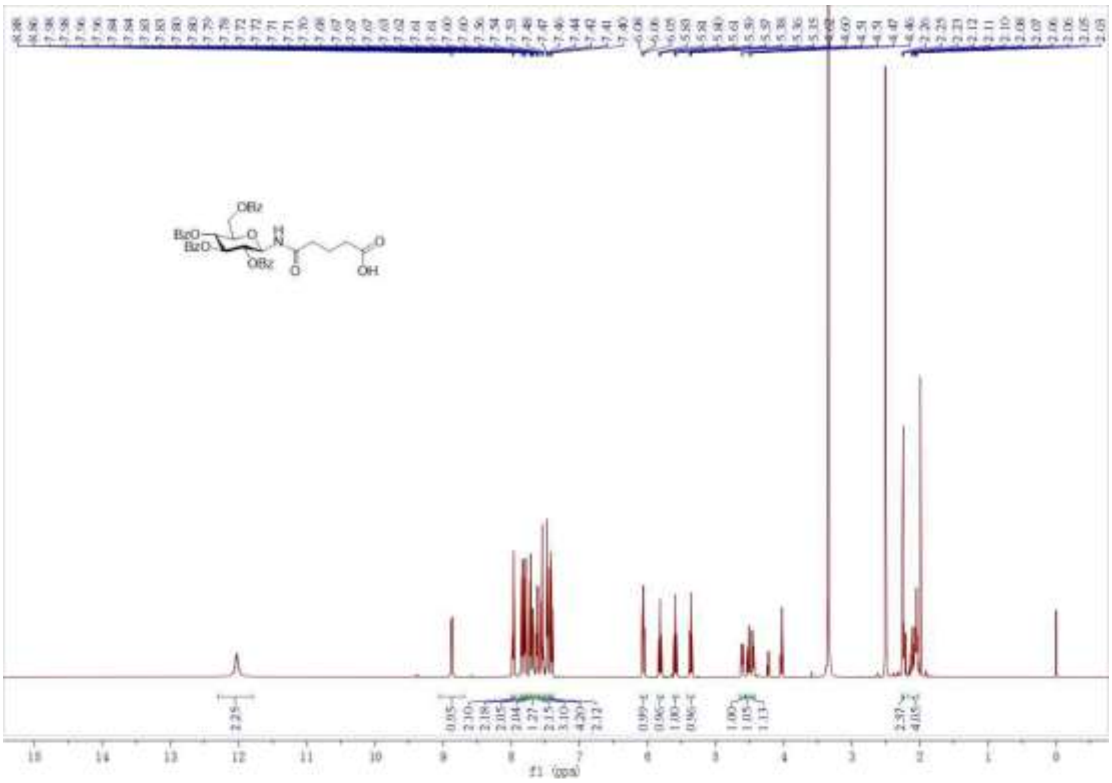

Figure S3. The ¹H-NMR of intermediate 5b.

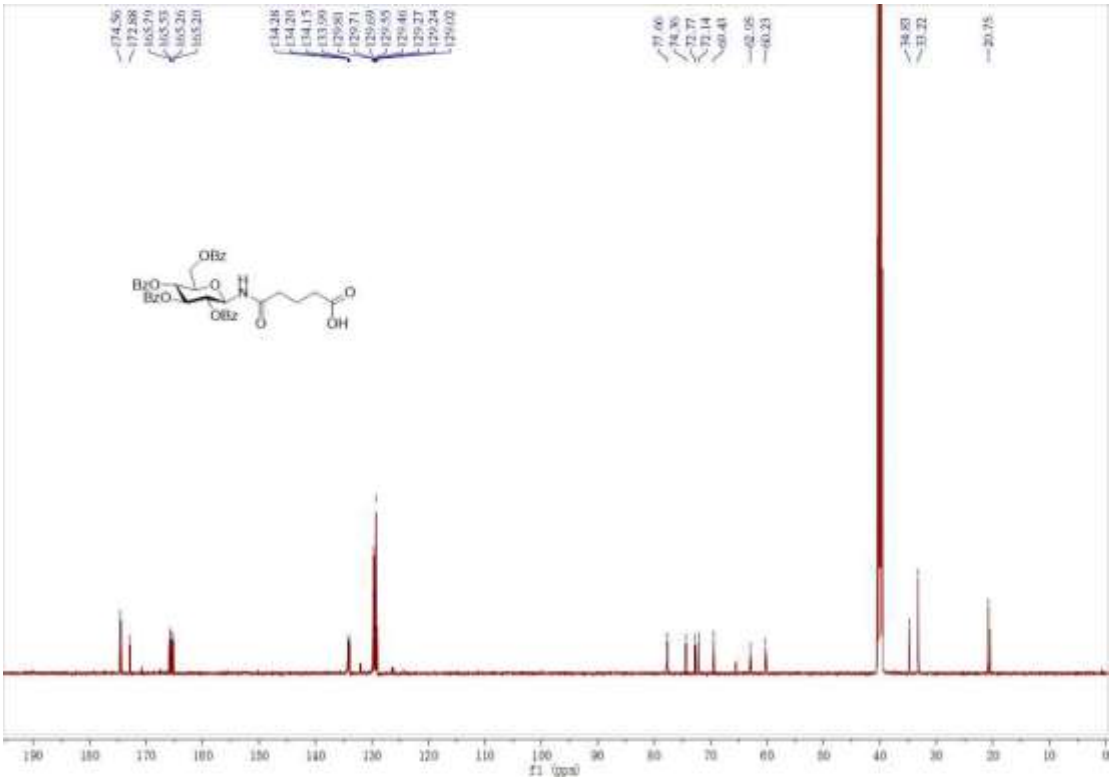

Figure 4. The ¹³C-NMR of intermediate 5b.

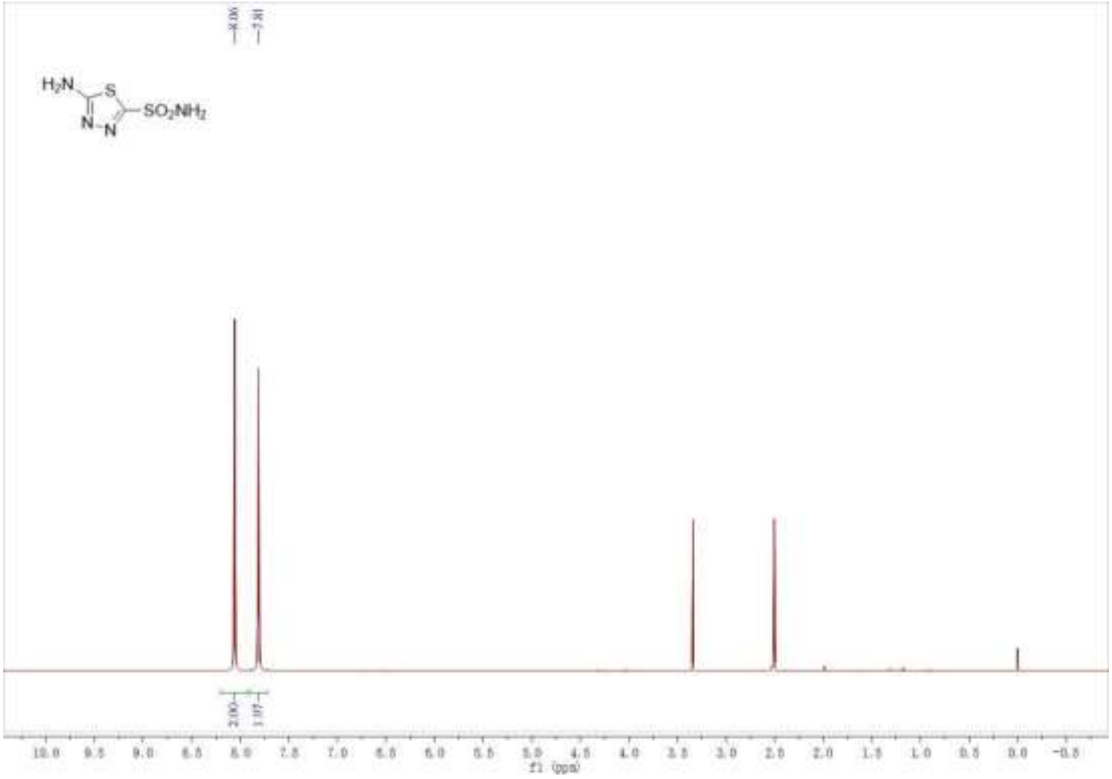

Figure S5. The ^{1}H-NMR of intermediate 10.

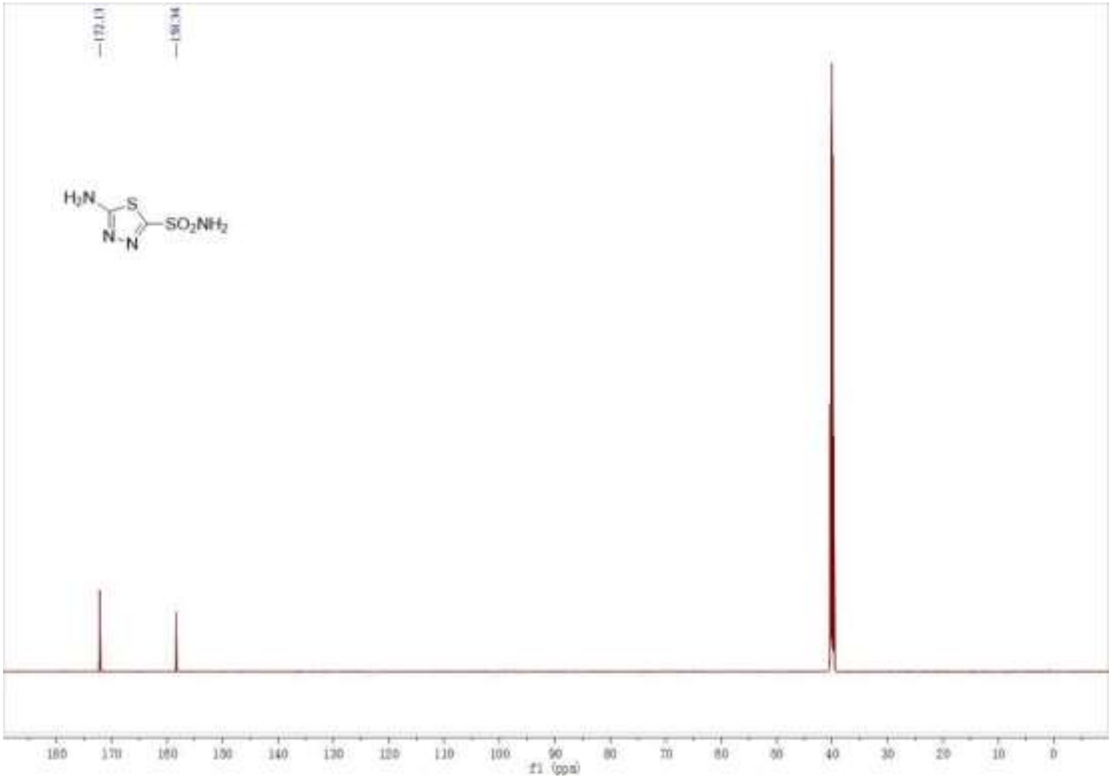

Figure S6. The ^{13}C-NMR of intermediate 10.

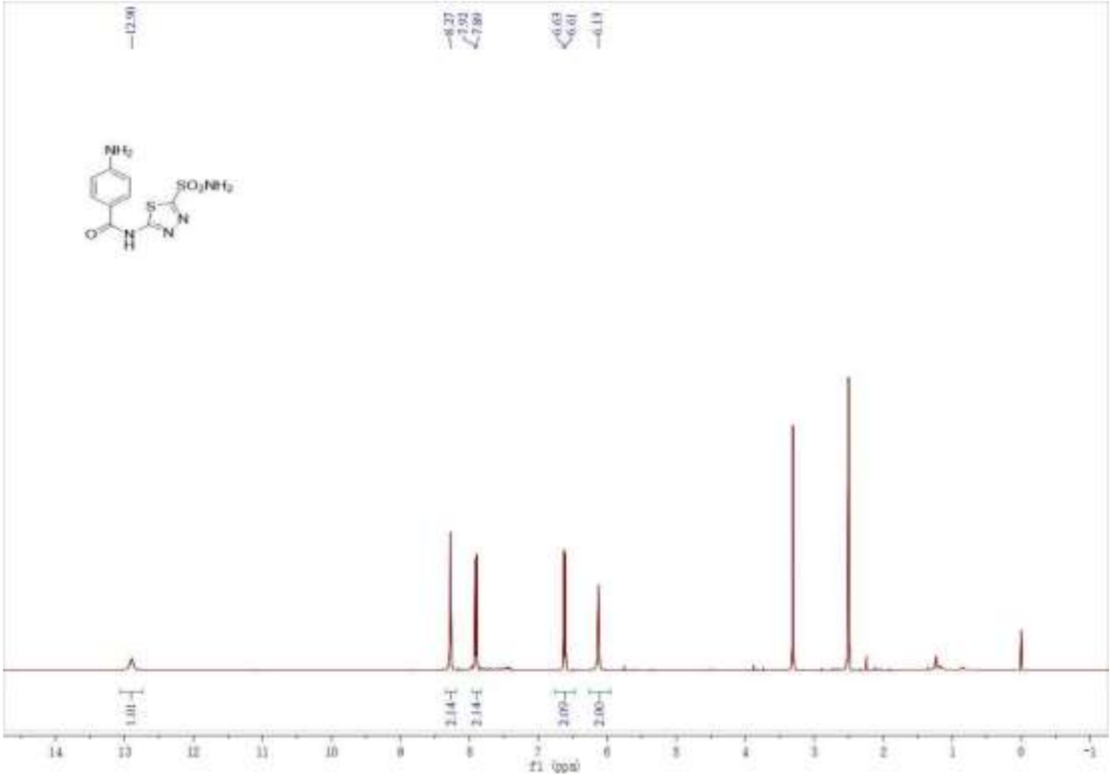

Figure S7. The ^{1}H-NMR of intermediate 12a.

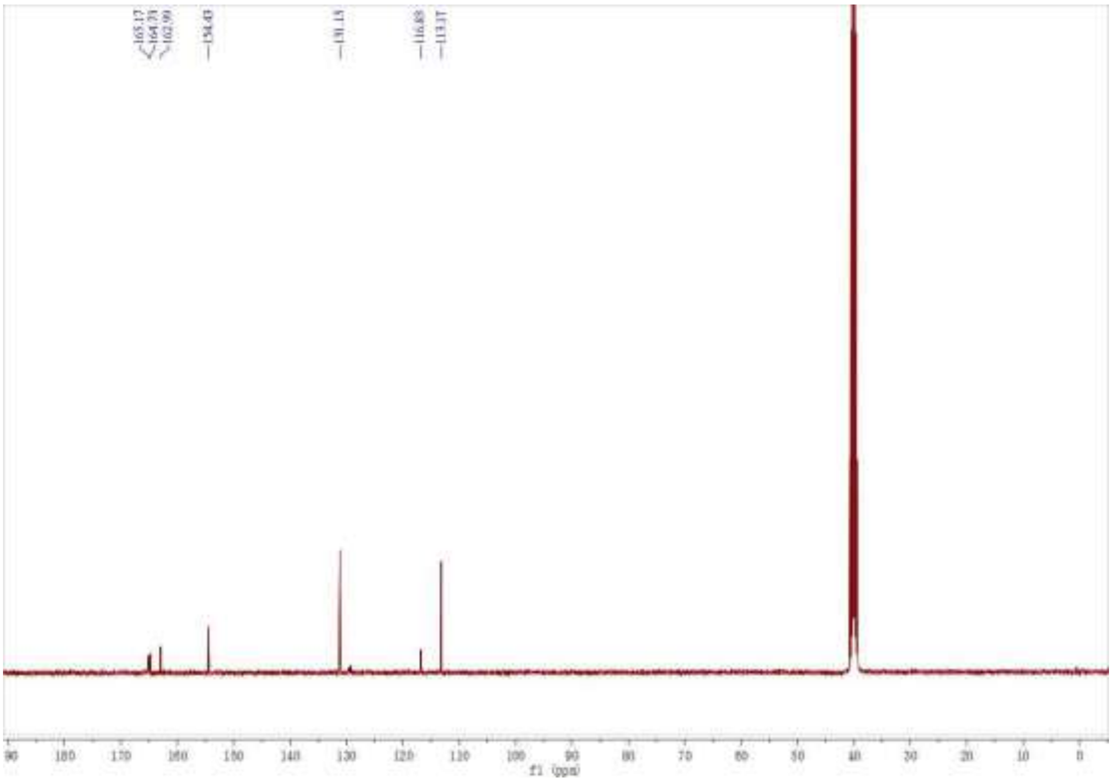

Figure S8. The ^{13}C-NMR of intermediate 12a.

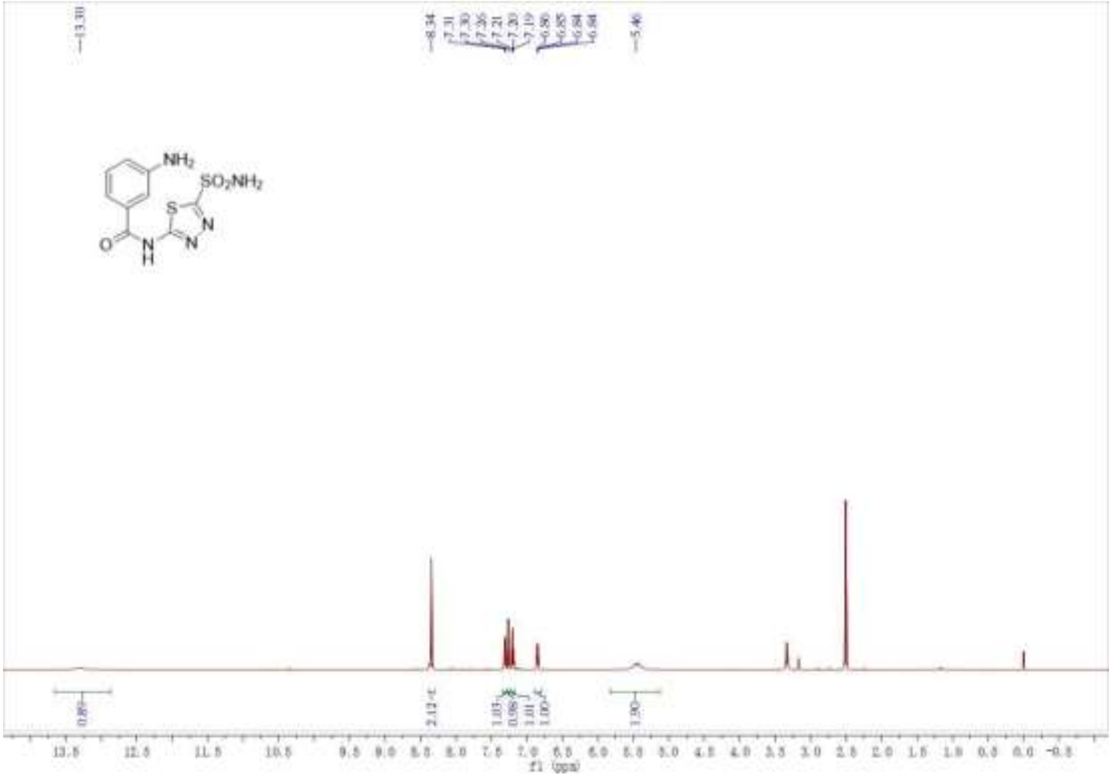

Figure S9. The ^{1}H-NMR of intermediate 12b.

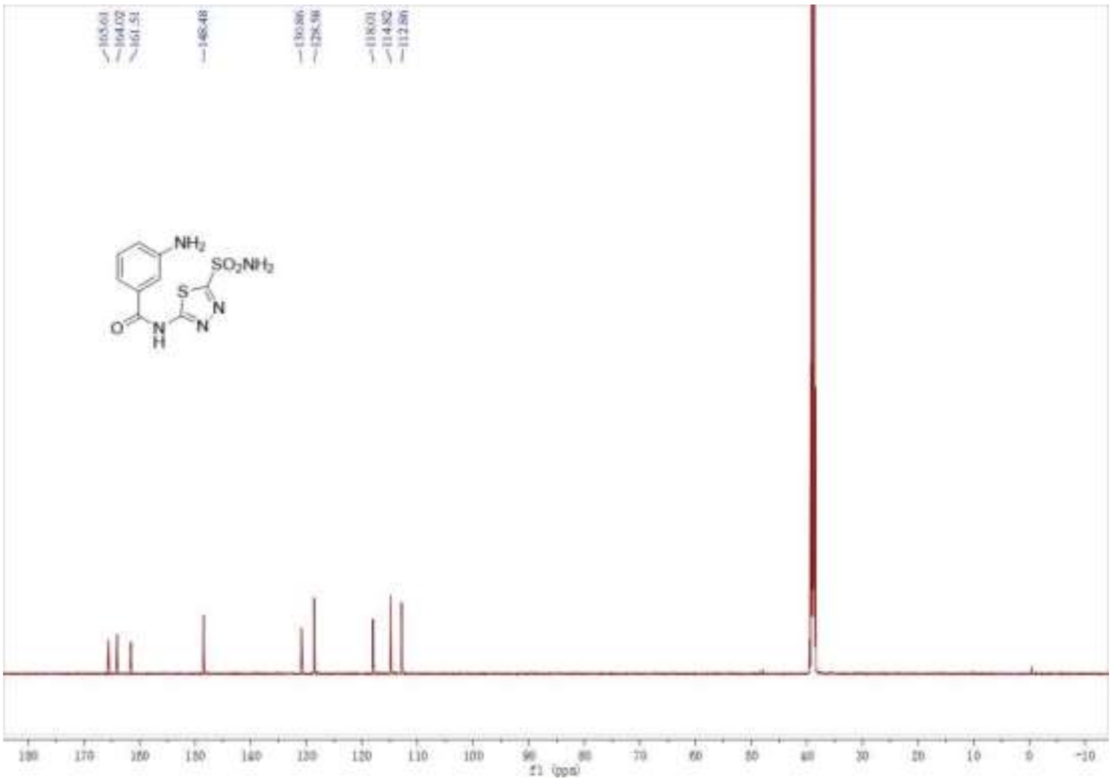

Figure S10. The ^{13}C-NMR of intermediate 12b.

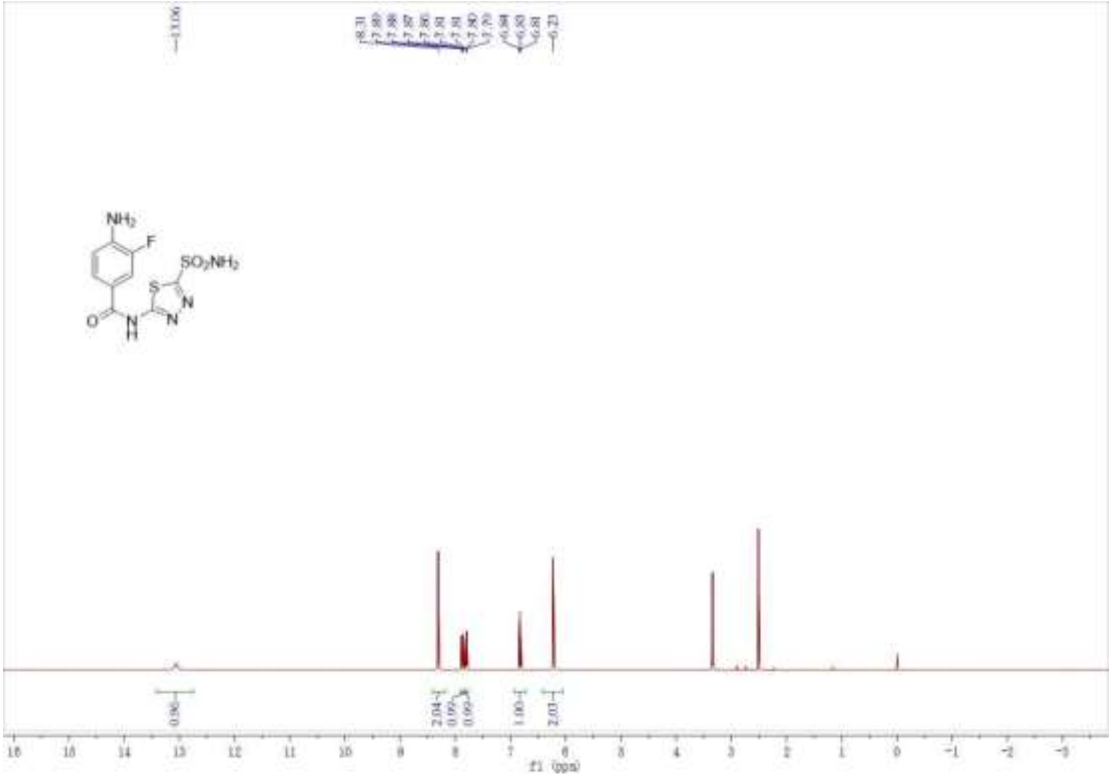

Figure S11. The ^{1}H-NMR of intermediate 12c.

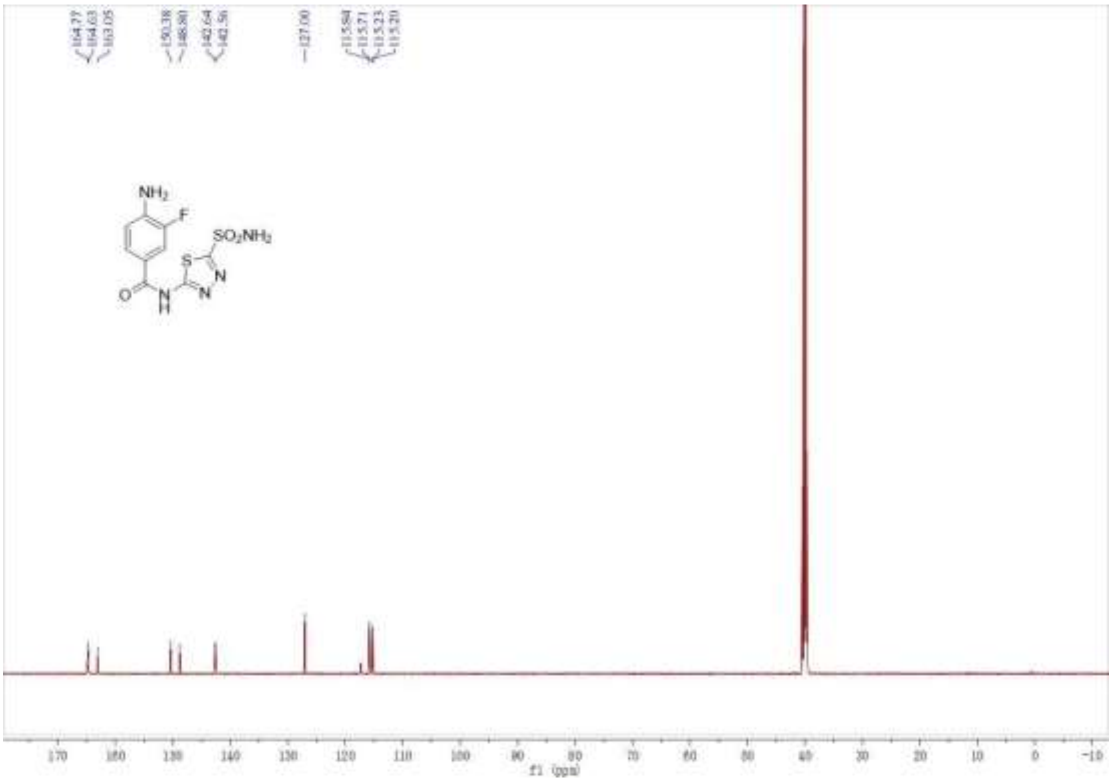

Figure S12. The ^{13}C-NMR of intermediate 12c

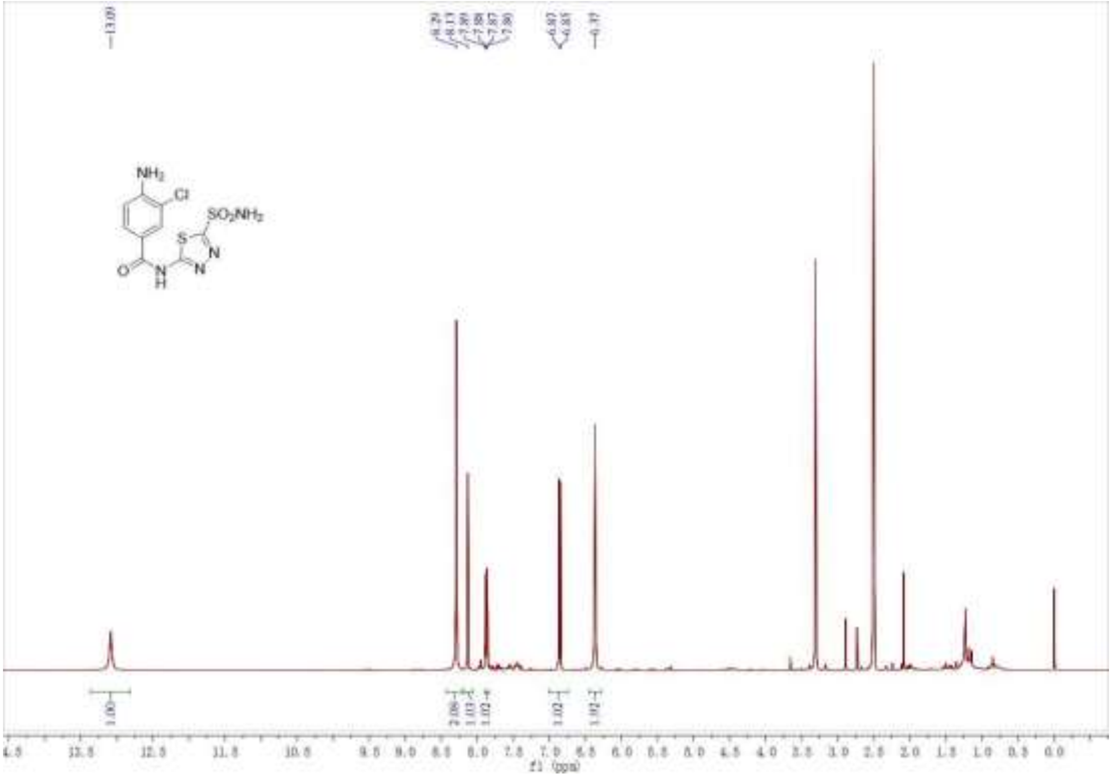

Figure S13. The ^{1}H-NMR of intermediate 12d.

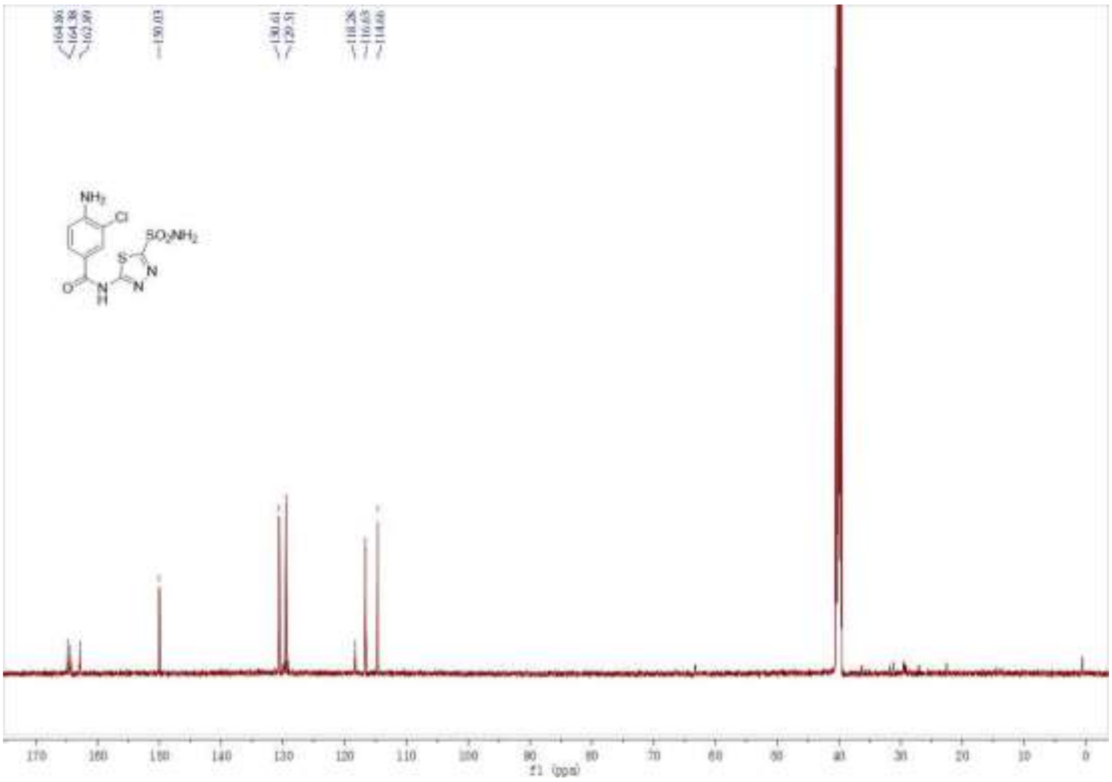

Figure S14. The ^{13}C-NMR of intermediate 12d.

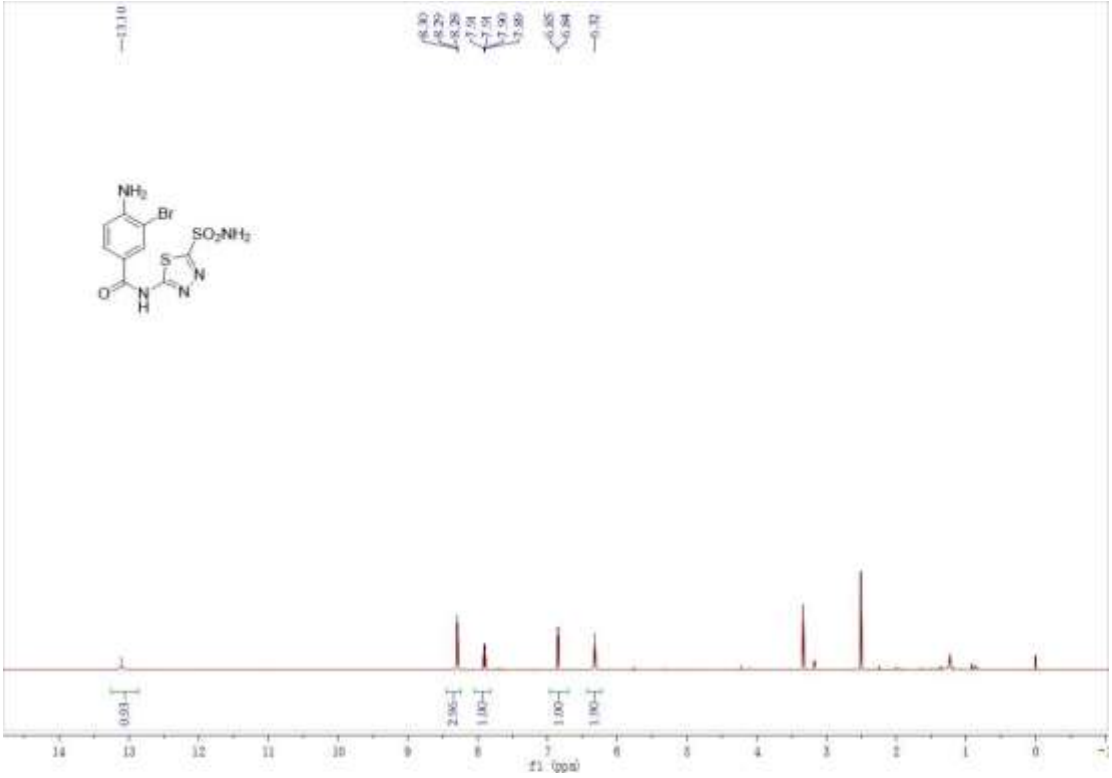

Figure S15. The ^{1}H-NMR of intermediate 12e.

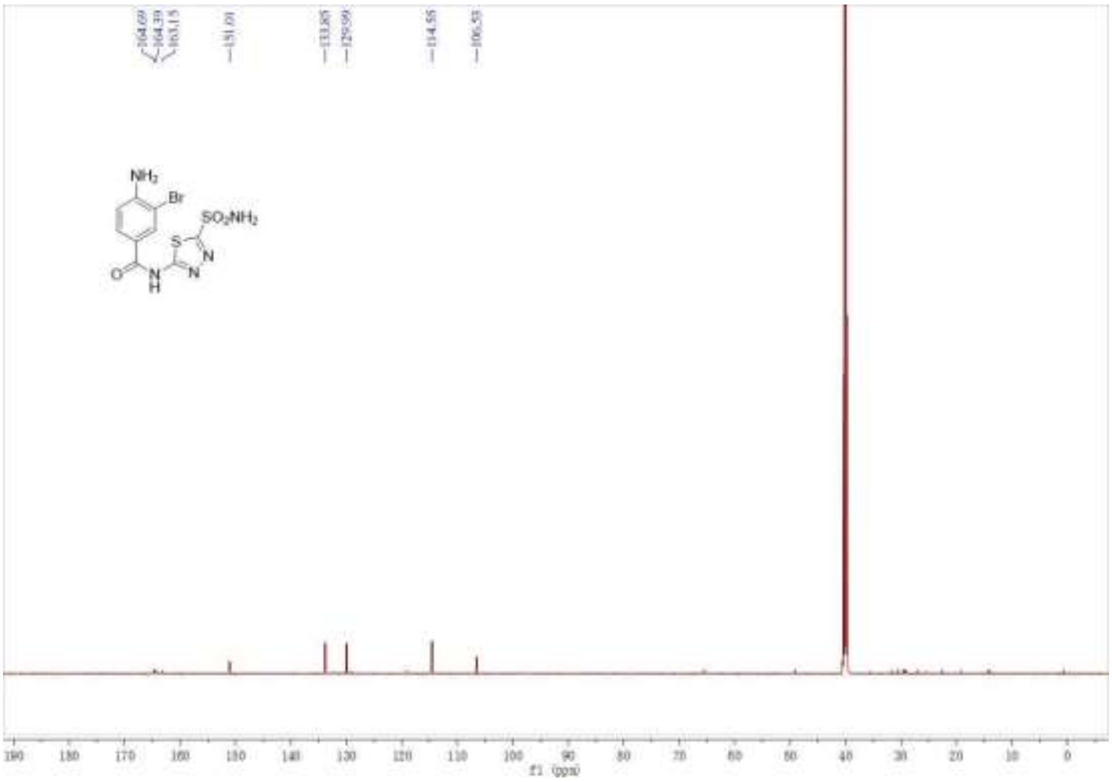

Figure S16. The ^{13}C-NMR of intermediate 12e.

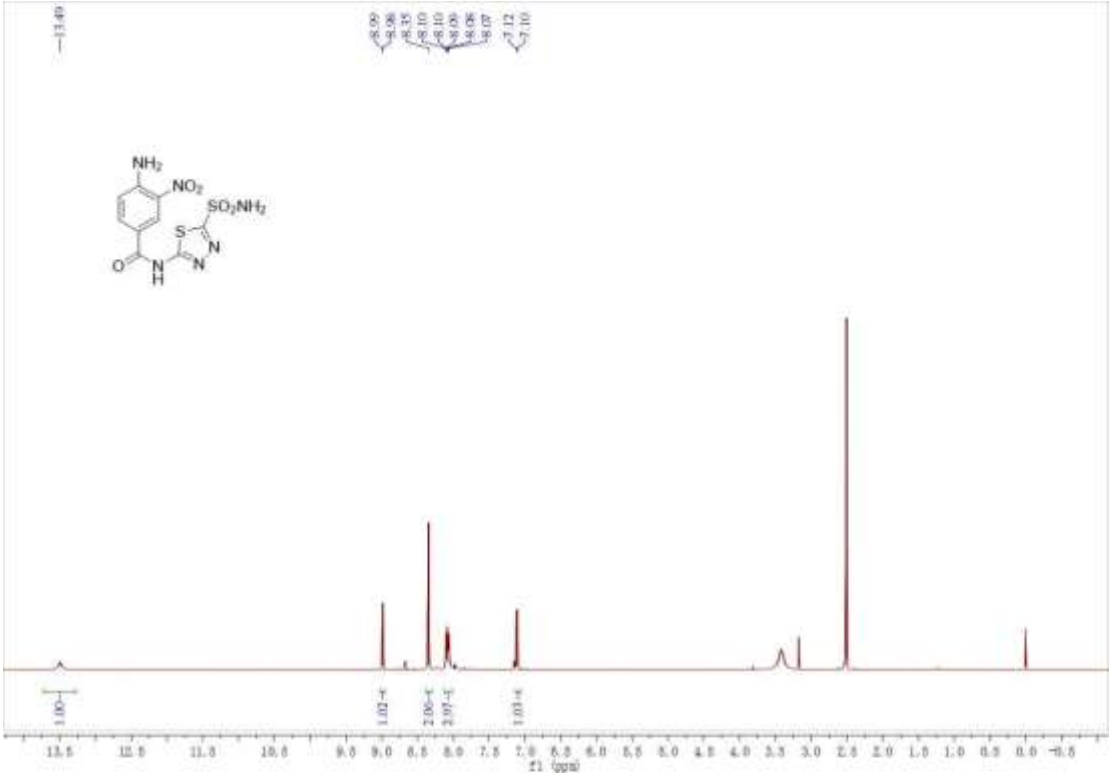

Figure S17. The ^{1}H-NMR of intermediate 12f.

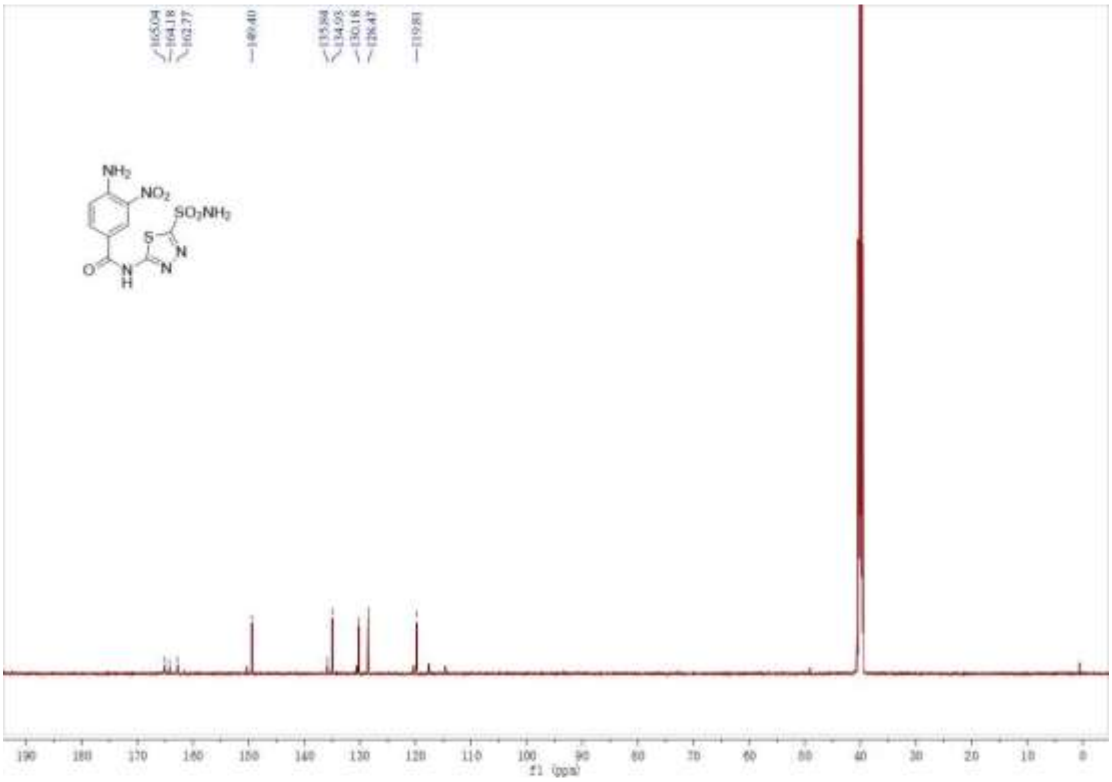

Figure S18. The ^{13}C-NMR of intermediate 12f.

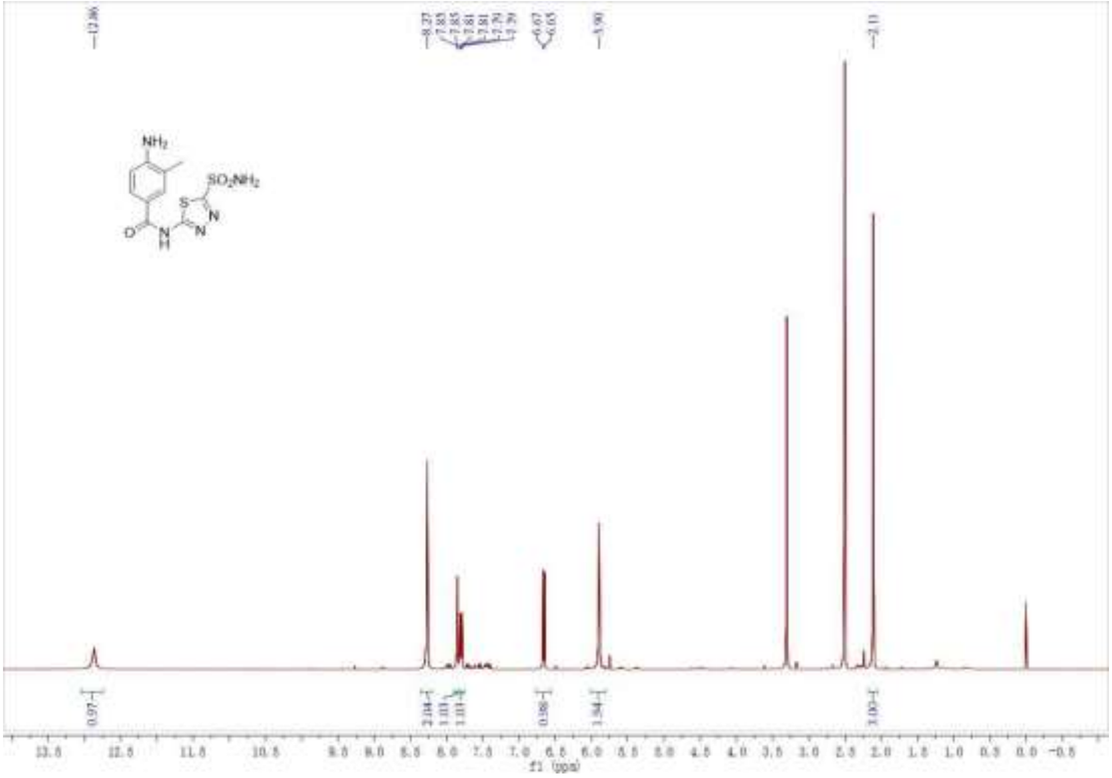

Figure S19. The ^{1}H-NMR of intermediate 12g.

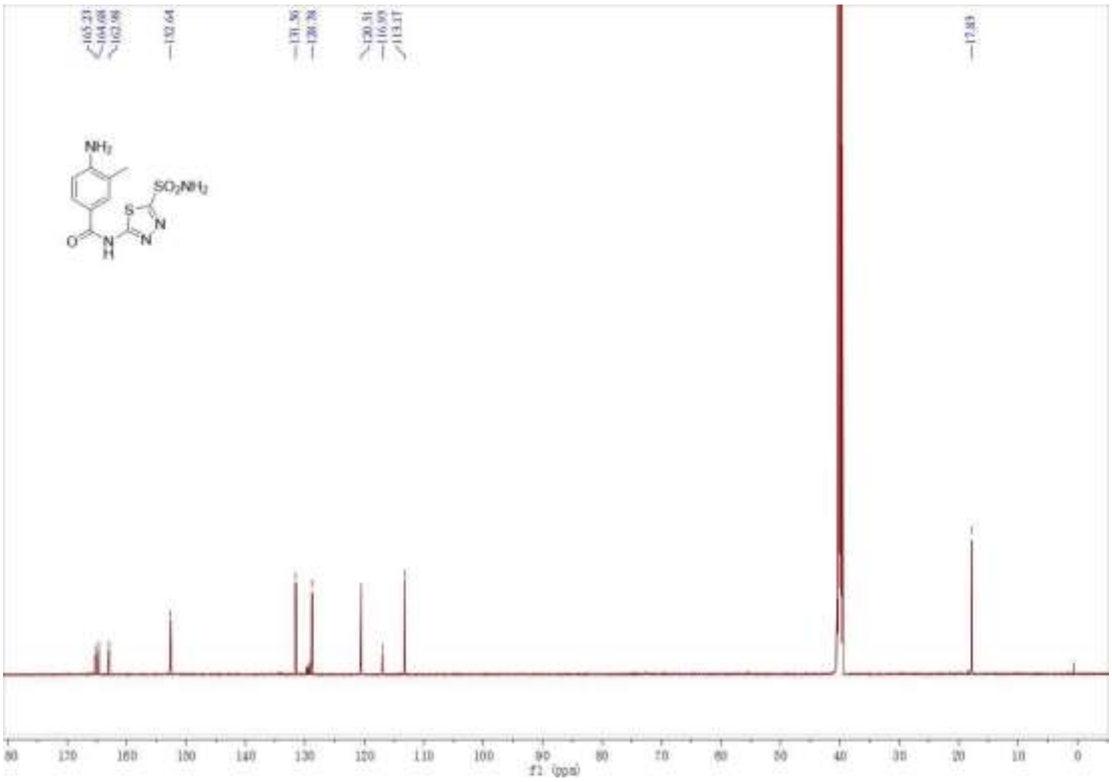

Figure S20. The ^{13}C-NMR of intermediate 12g.

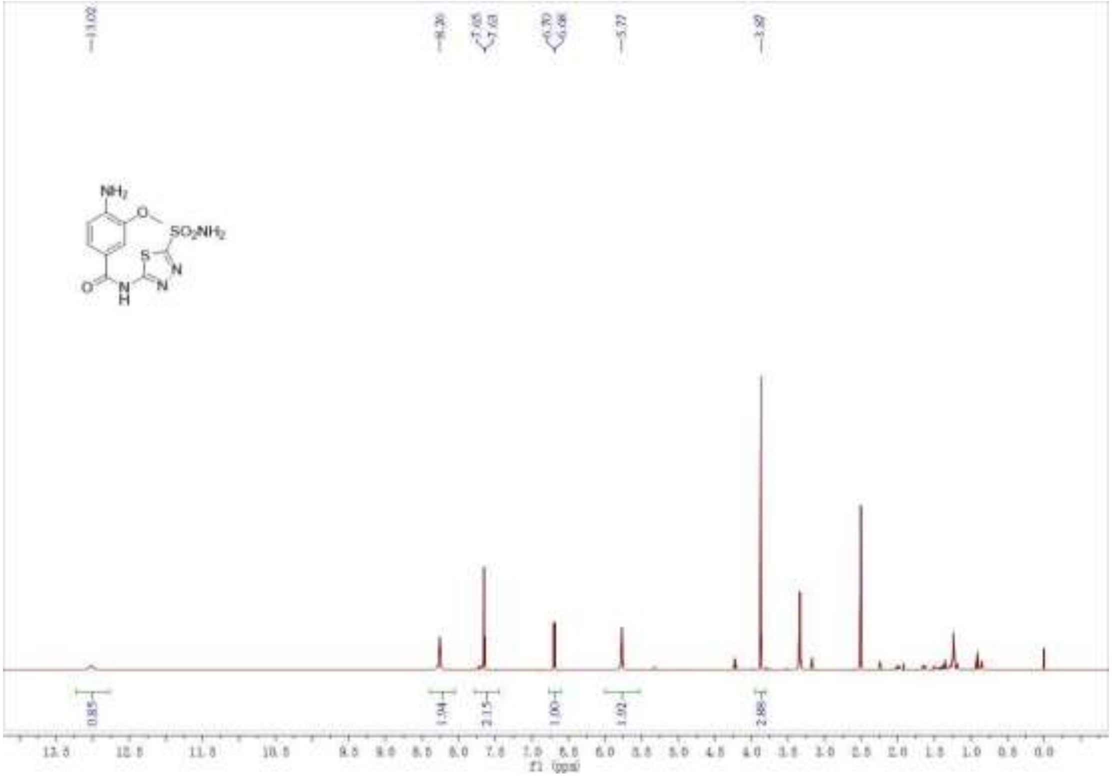

Figure S21. The ^{1}H-NMR of intermediate 12h.

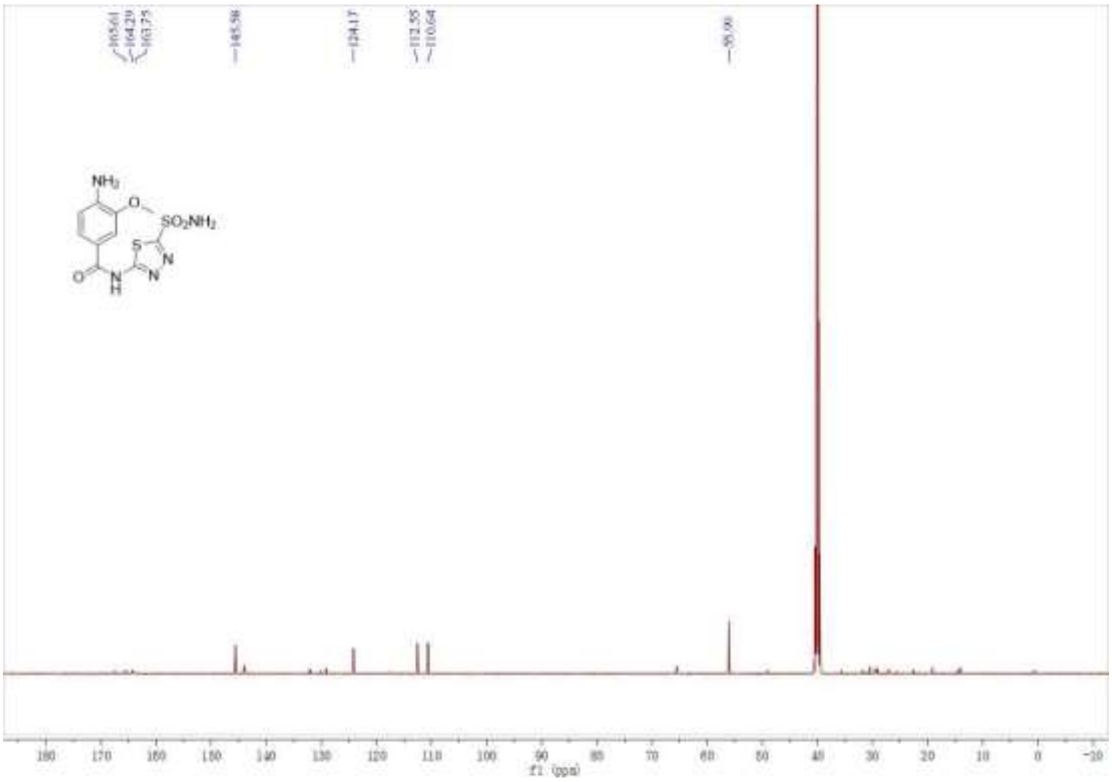

Figure S22. The ^{13}C-NMR of intermediate 12h.

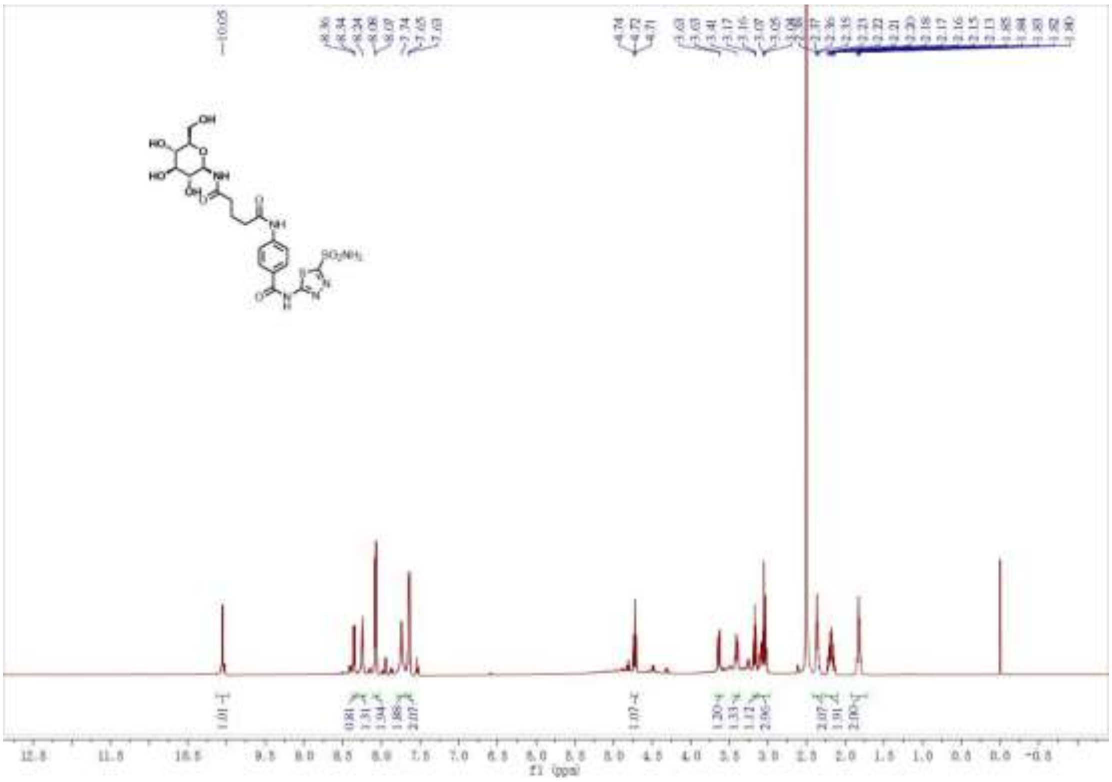

Figure S23. The ^{1}H-NMR of compound 14a.

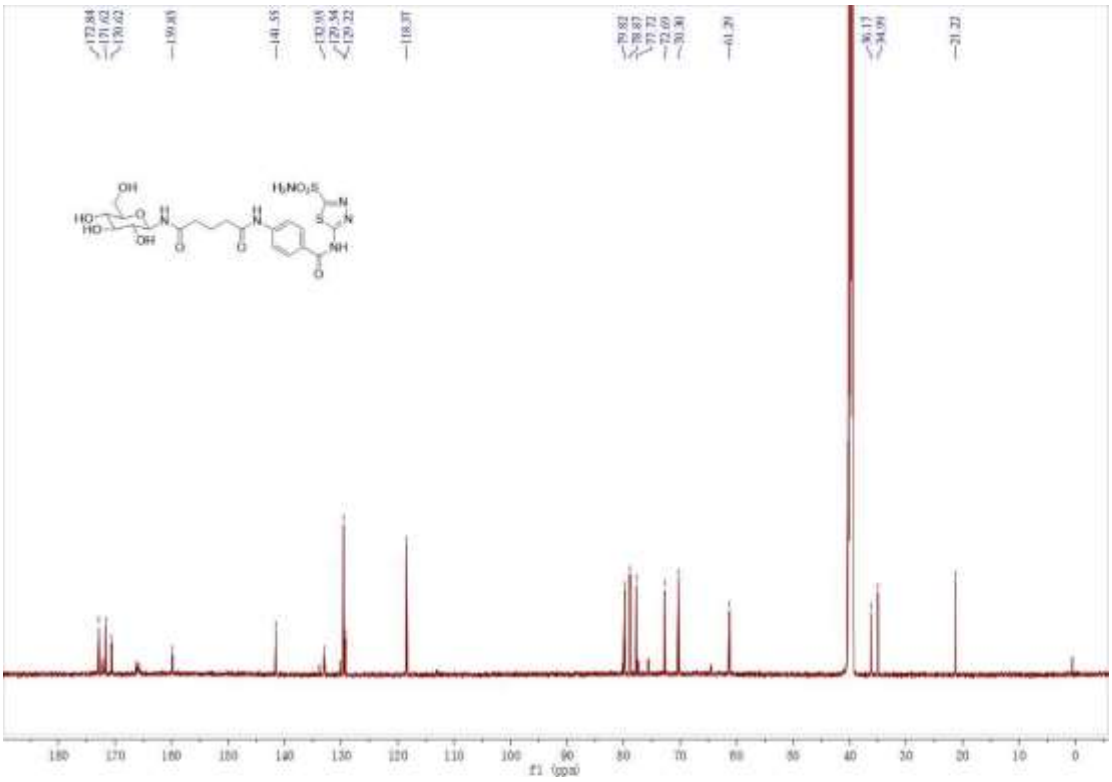

Figure S24. The ^{13}C-NMR of compound 14a.

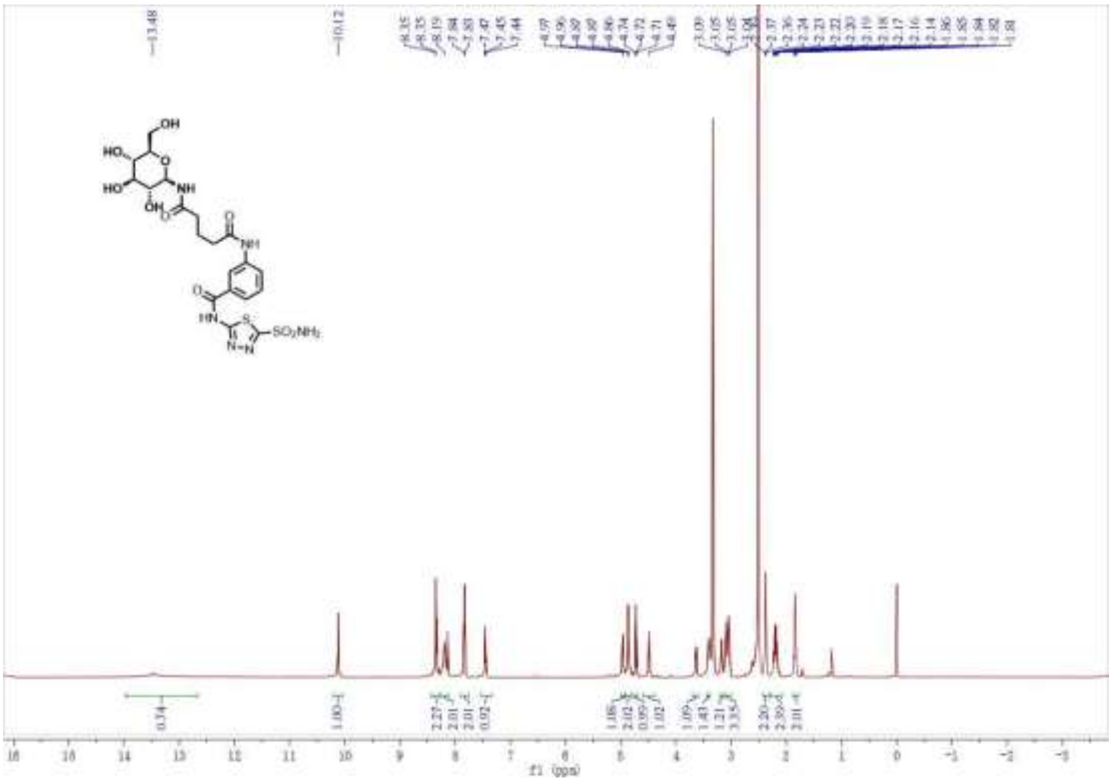

Figure S25. The ^{1}H-NMR of compound 14b.

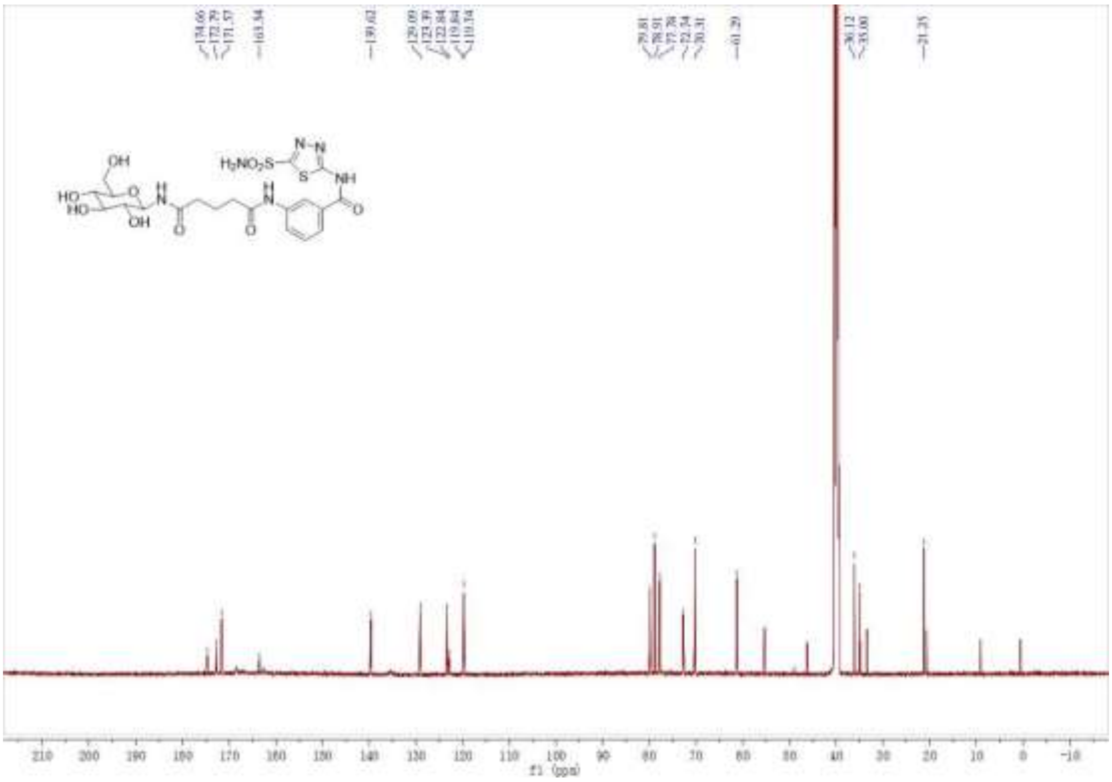

Figure S26. The ^{13}C-NMR of compound 14b.

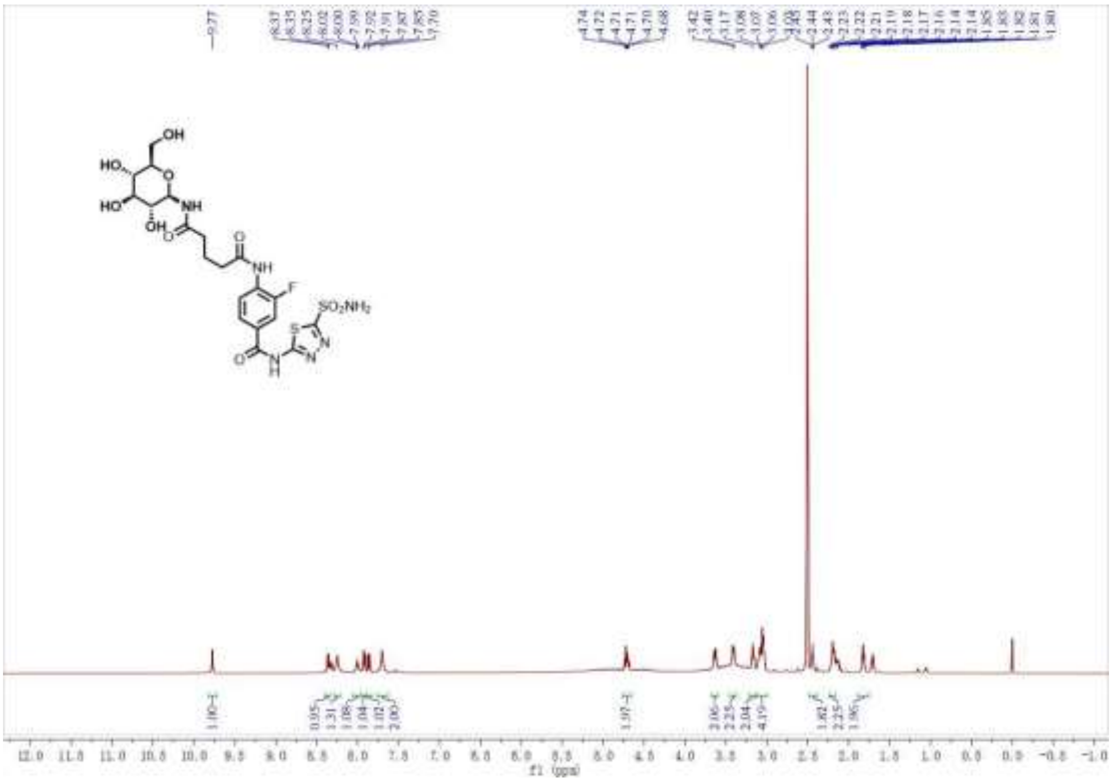

Figure S27. The ^{1}H-NMR of compound 14c.

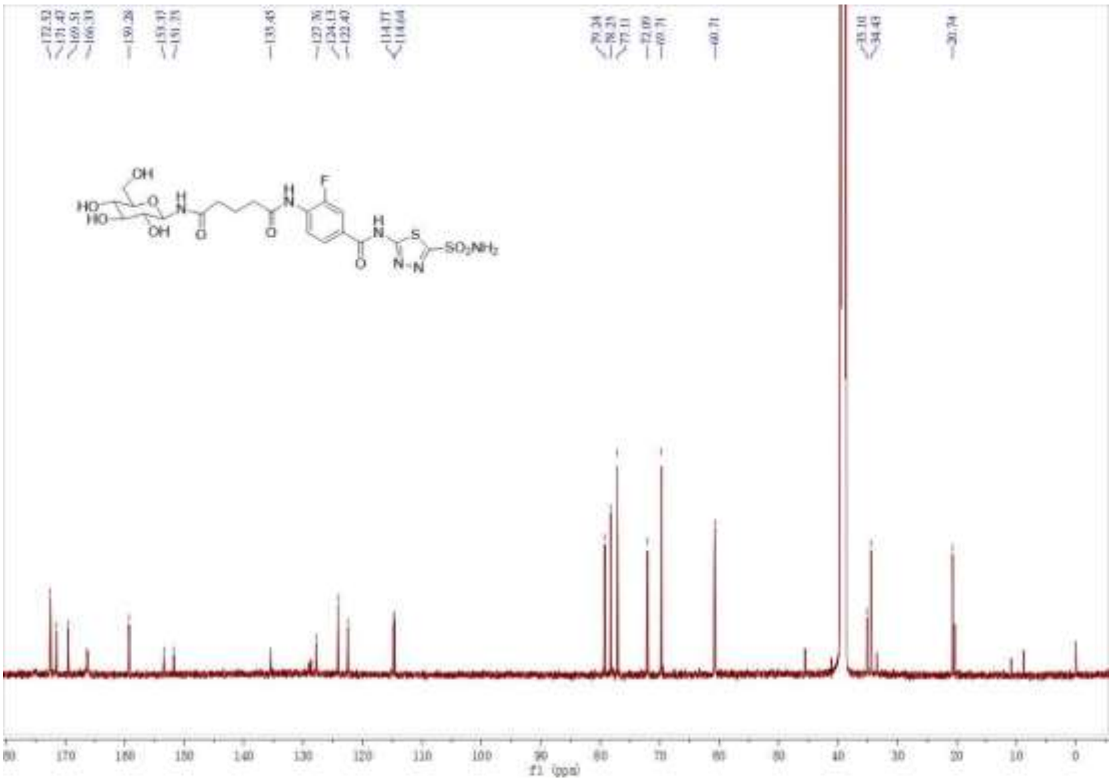

Figure S28. The ^{13}C-NMR of compound 14c.

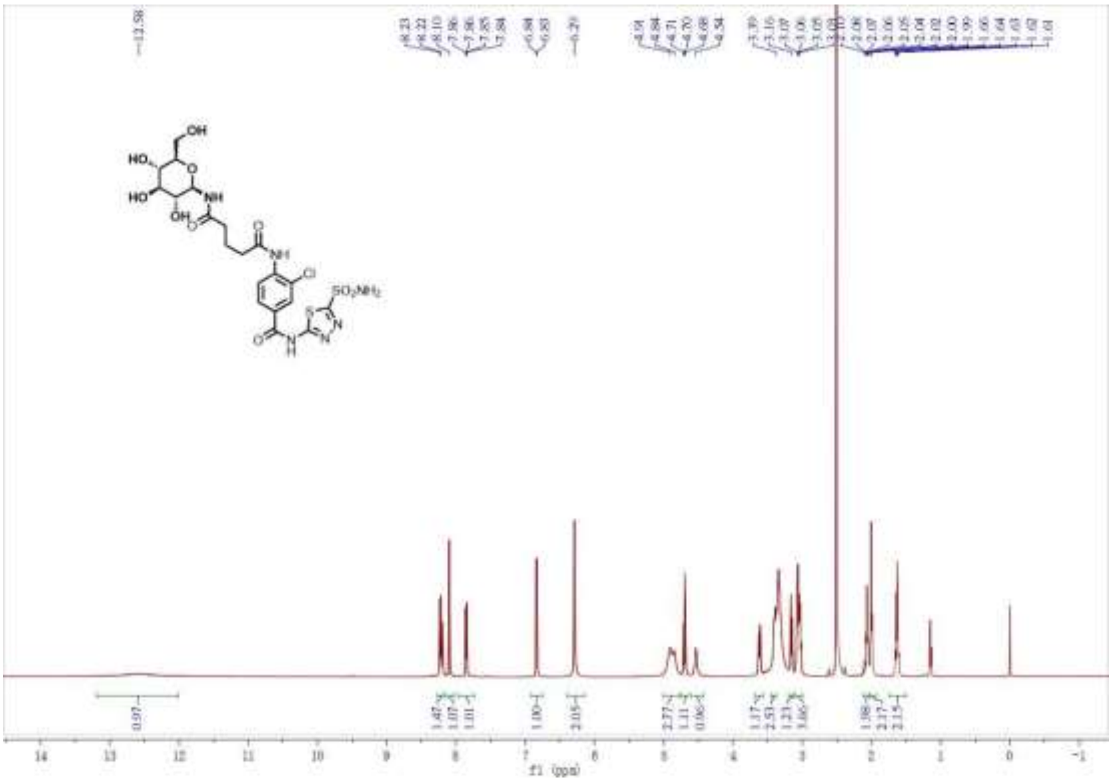

Figure S29. The ^{1}H-NMR of compound 14d.

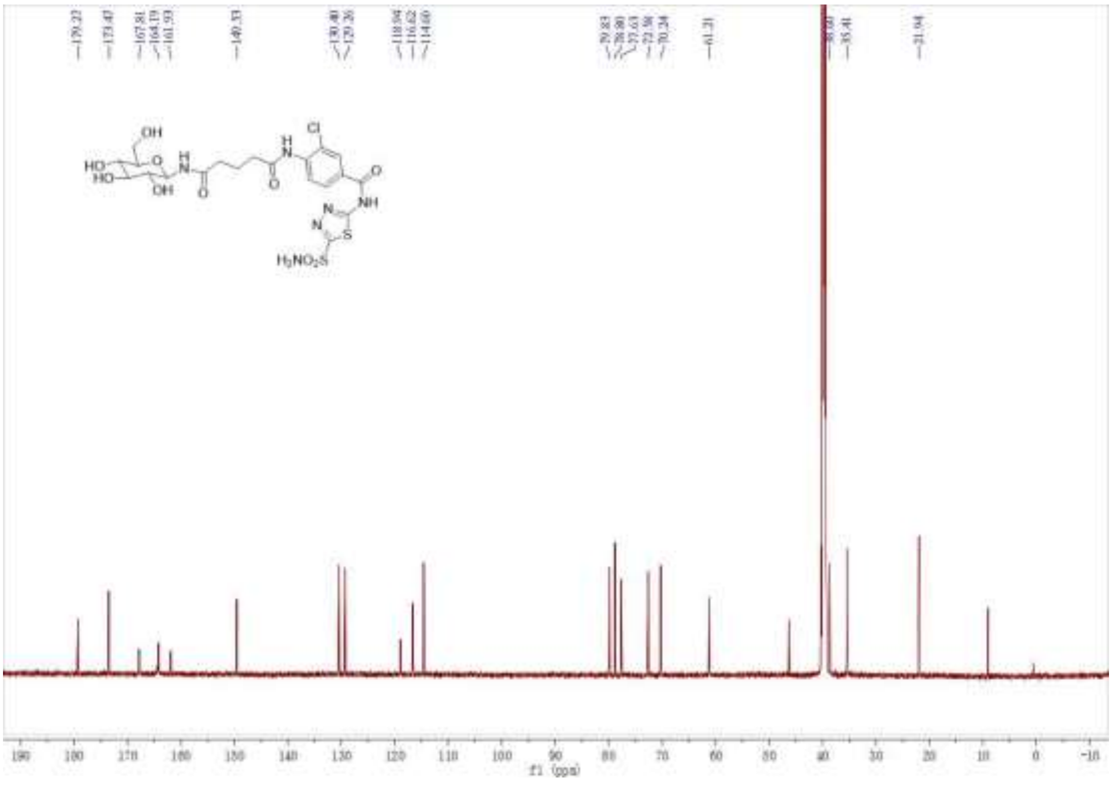

Figure S30. The ^{13}C-NMR of compound 14d.

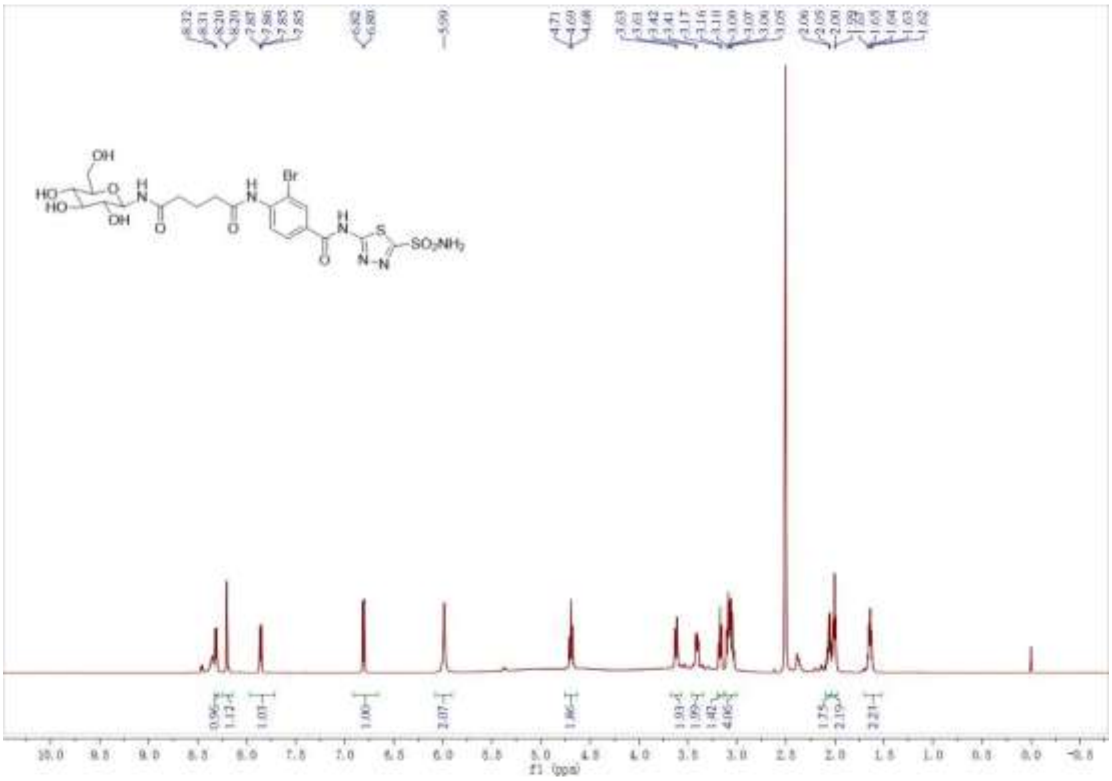

Figure S31. The ^{1}H-NMR of compound 14e.

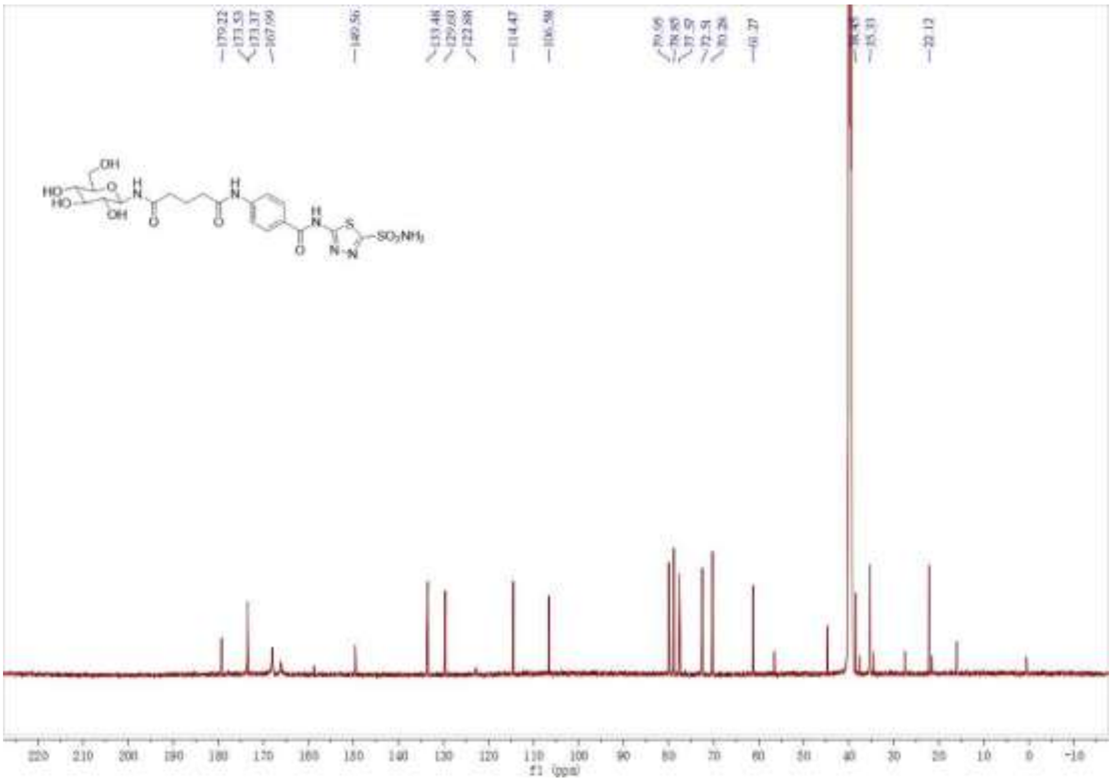

Figure S32. The ^{13}C-NMR of compound 14e.

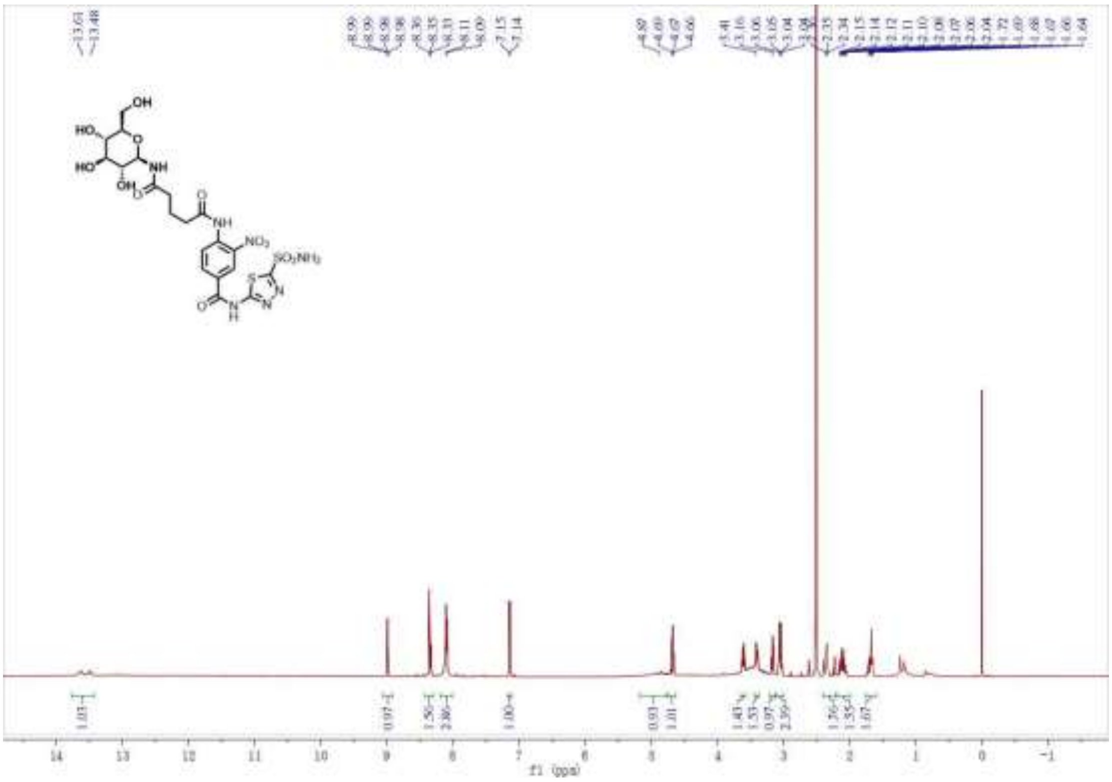

Figure S33. The ^{1}H-NMR of compound 14f.

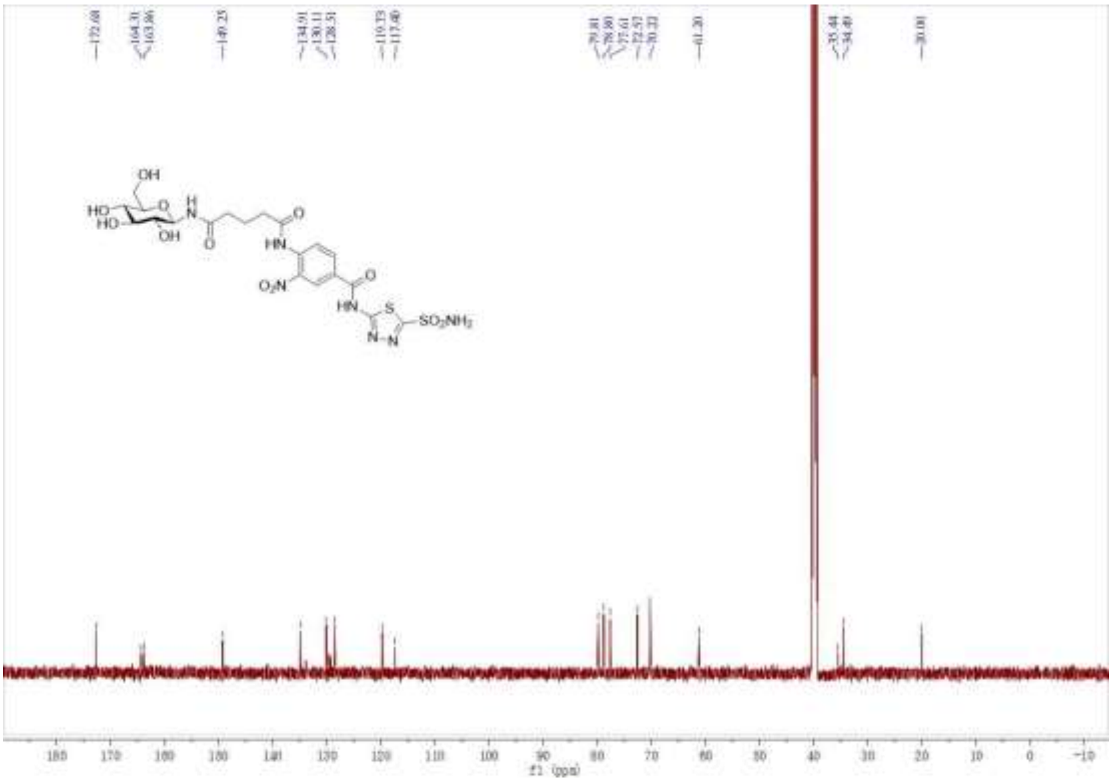

Figure S34. The ^{13}C-NMR of compound 14f.

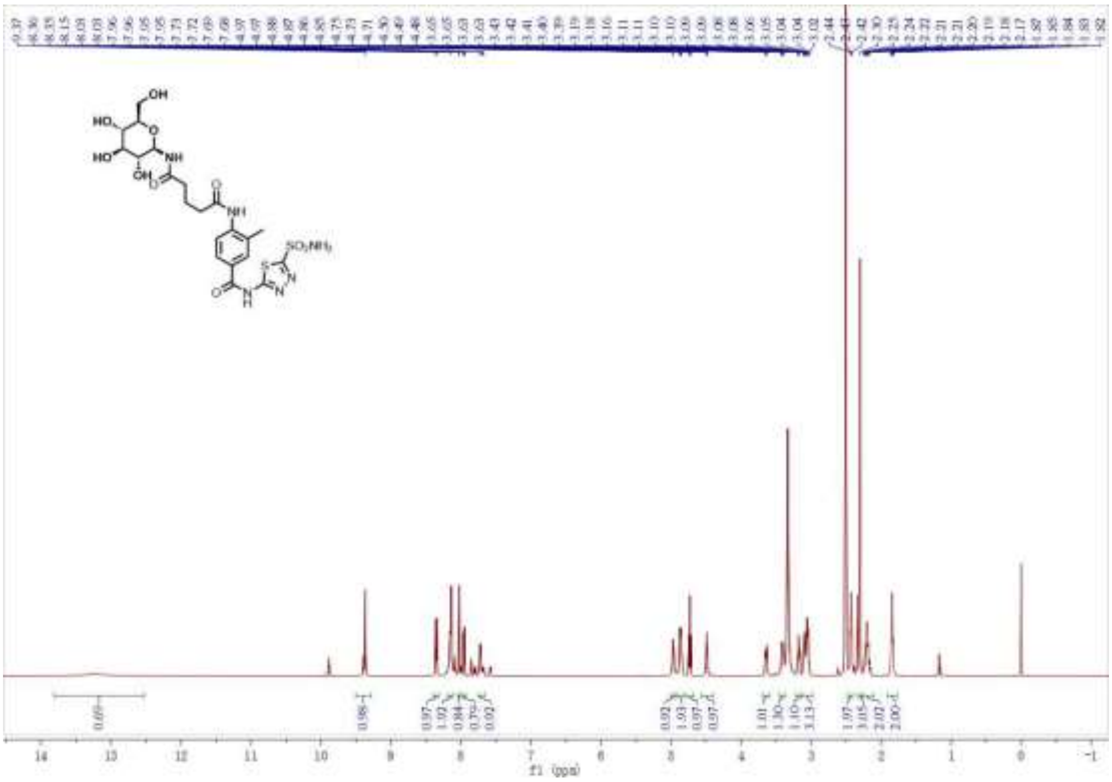

Figure S35. The ^{1}H-NMR of compound 14g.

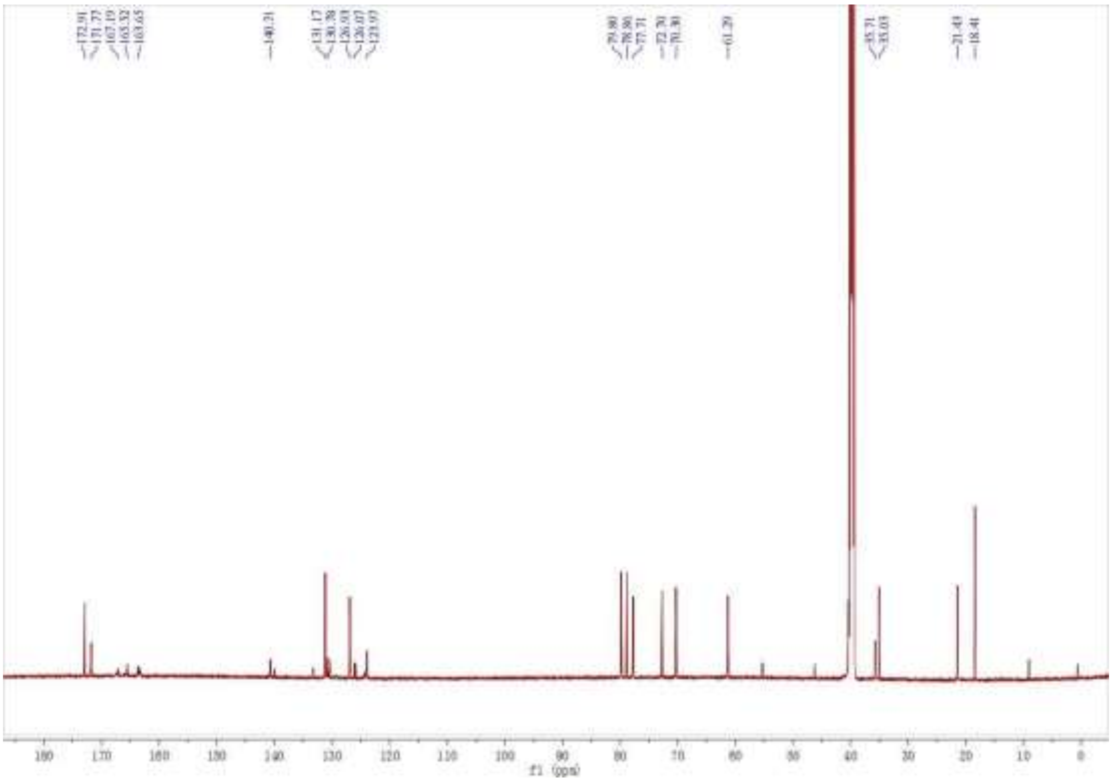

Figure S36. The ^{13}C-NMR of compound 14g.

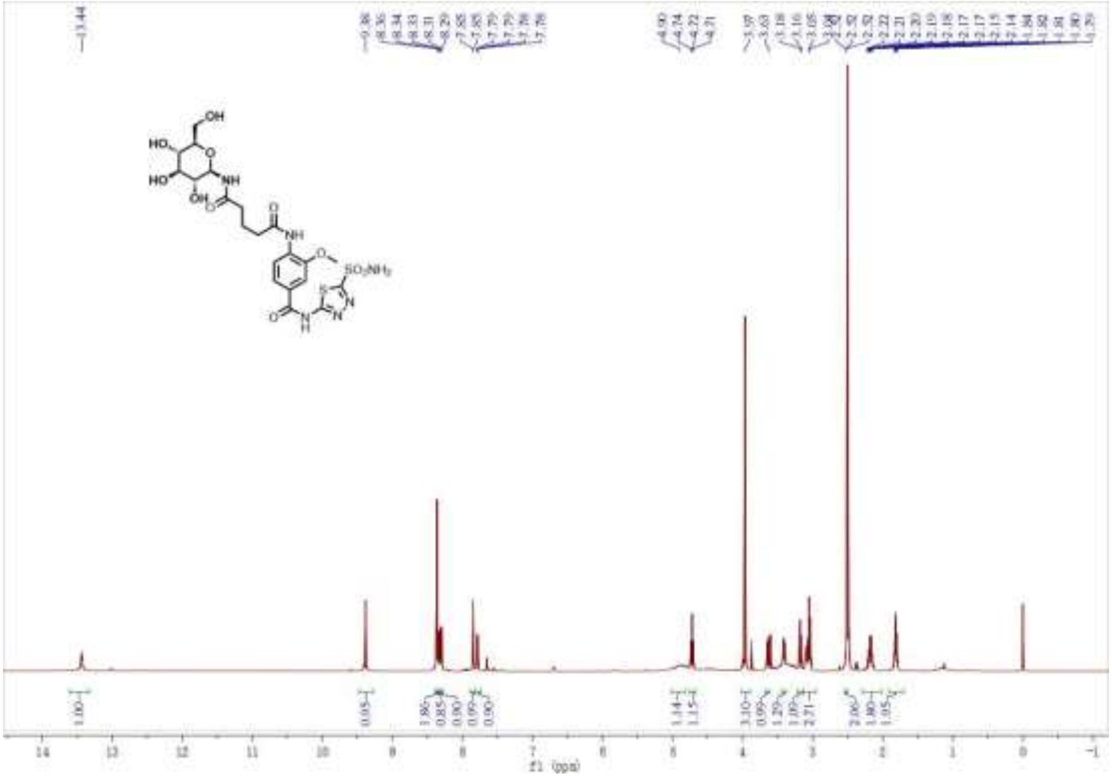

Figure S37. The ^{1}H-NMR of compound 14h.

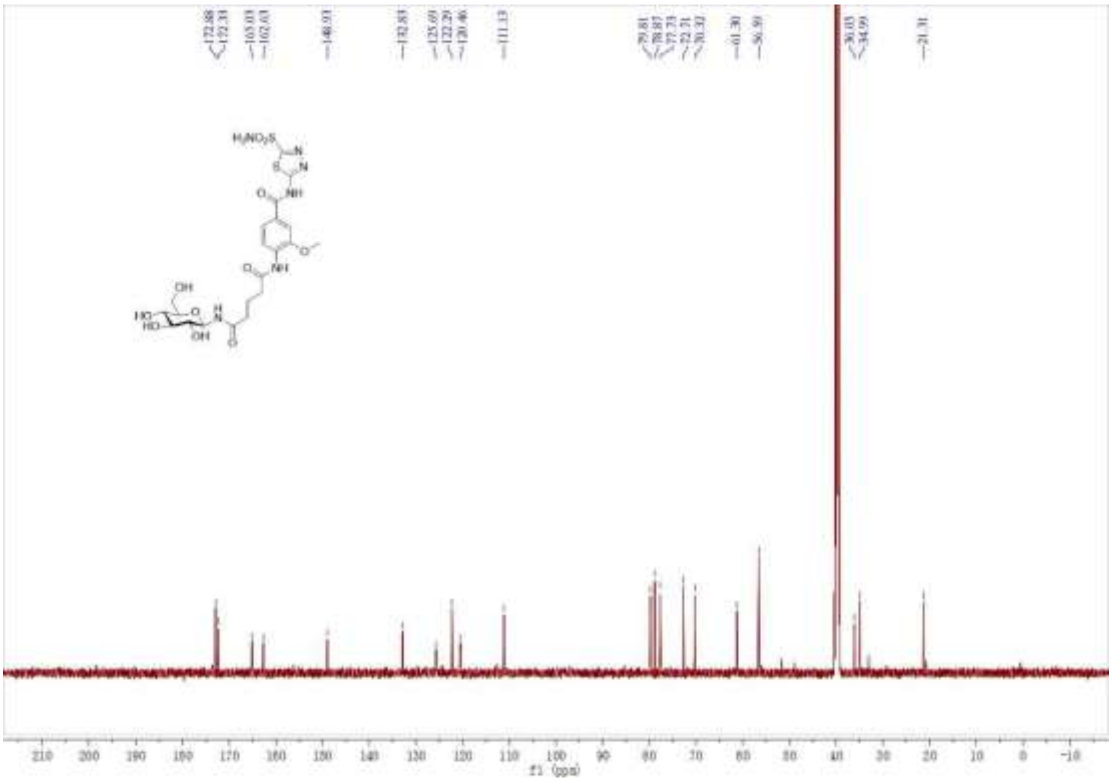

Figure S38. The ^{13}C-NMR of compound 14h.

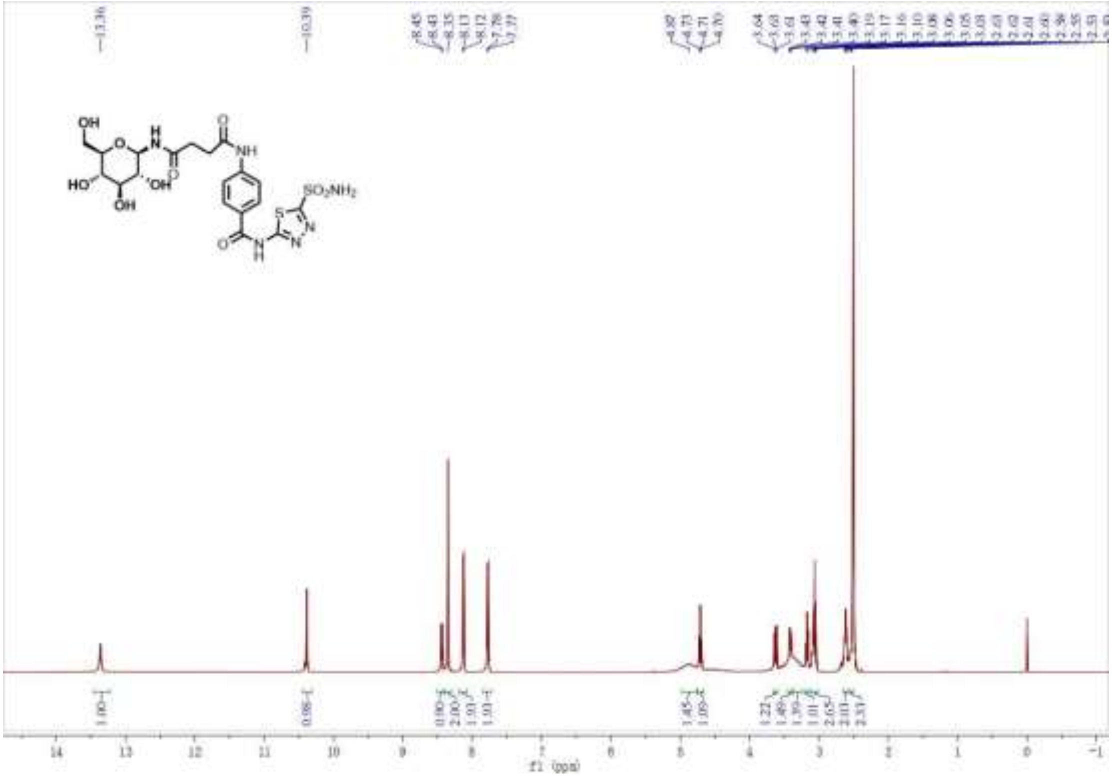

Figure S39. The ^{1}H-NMR of compound 14i.

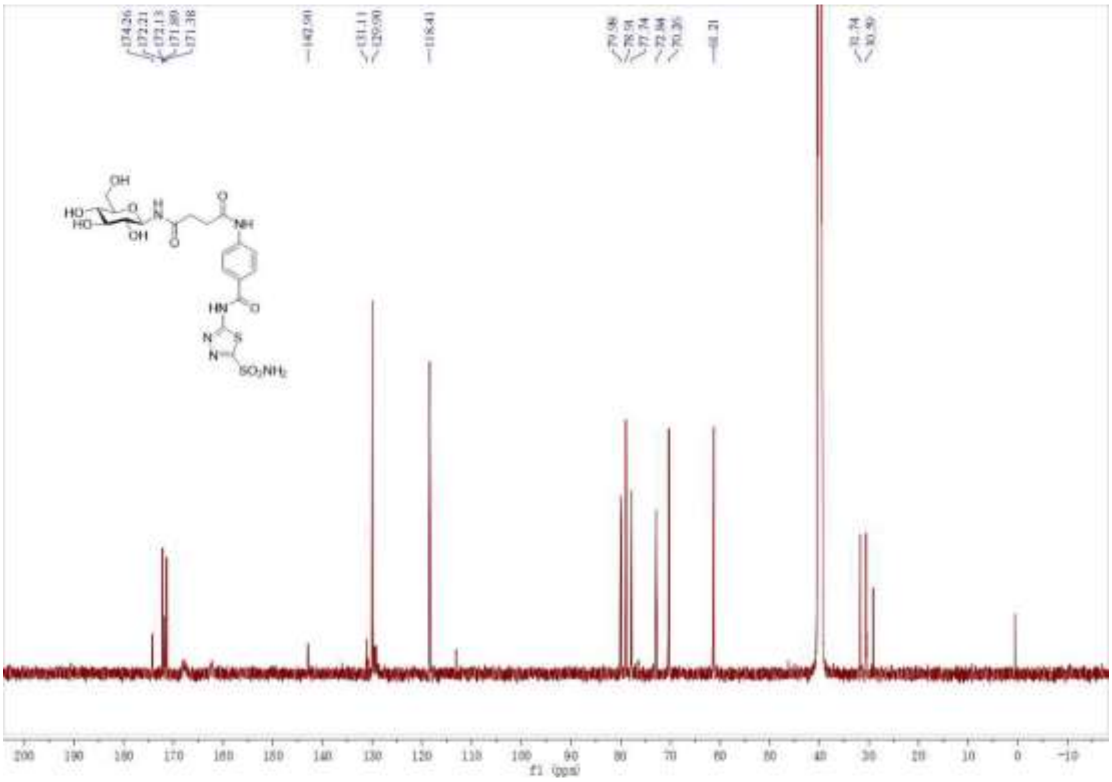

Figure S40. The ^{13}C-NMR of compound 14i.

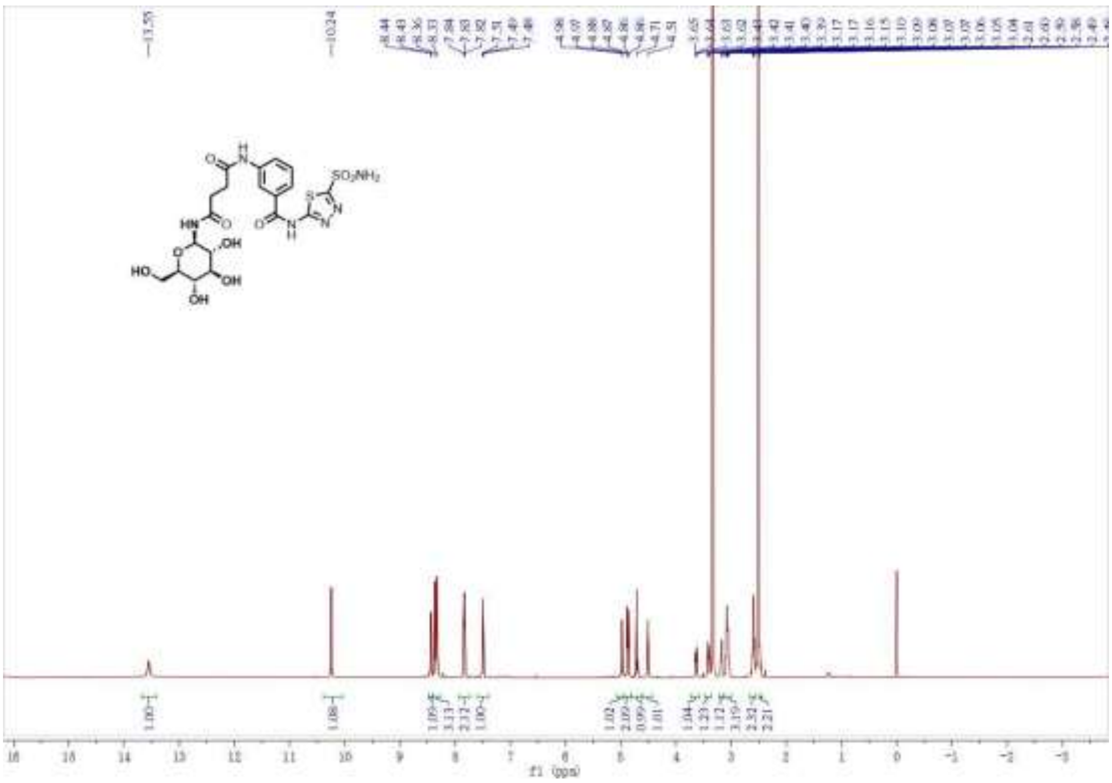

Figure S41. The ^{1}H-NMR of compound 14j.

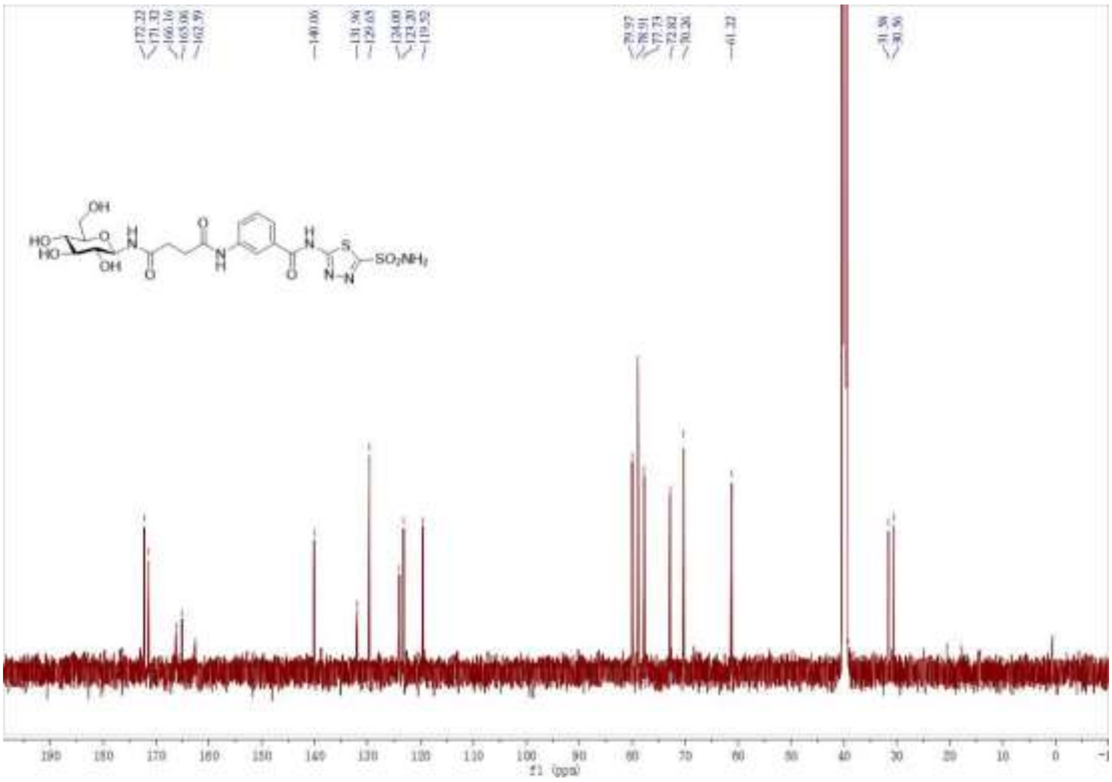

Figure S42. The ^{13}C-NMR of compound 14j.

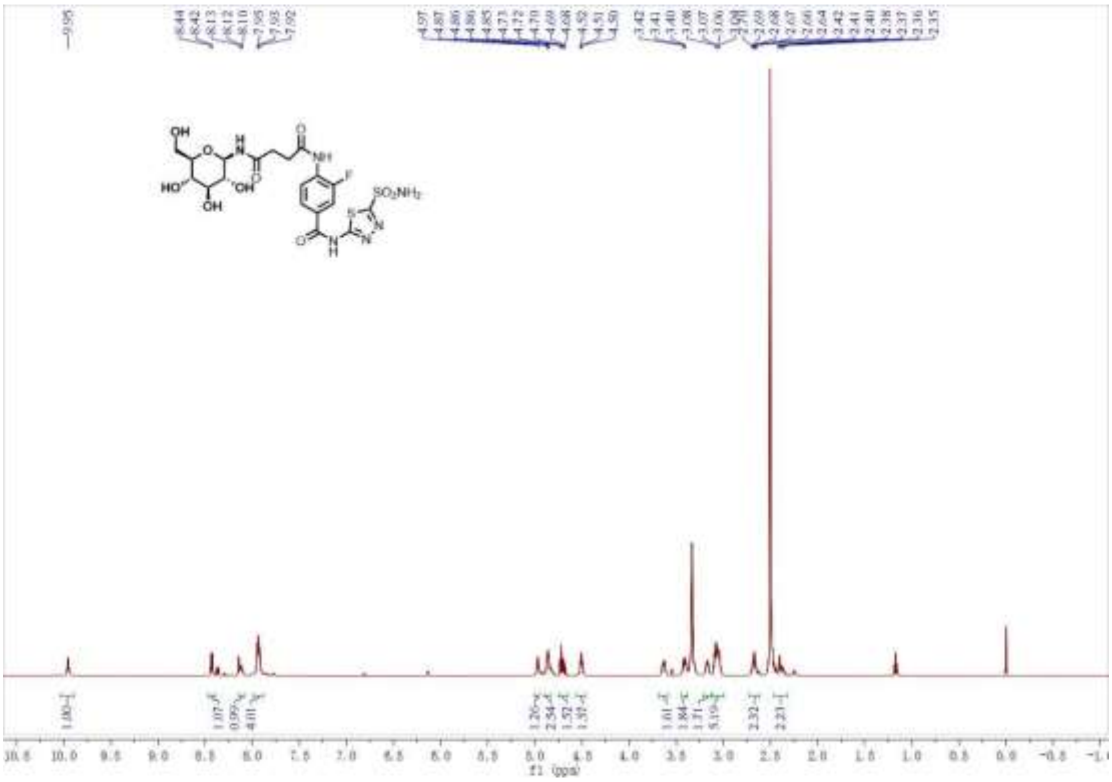

Figure S43. The ^{1}H-NMR of compound 14k.

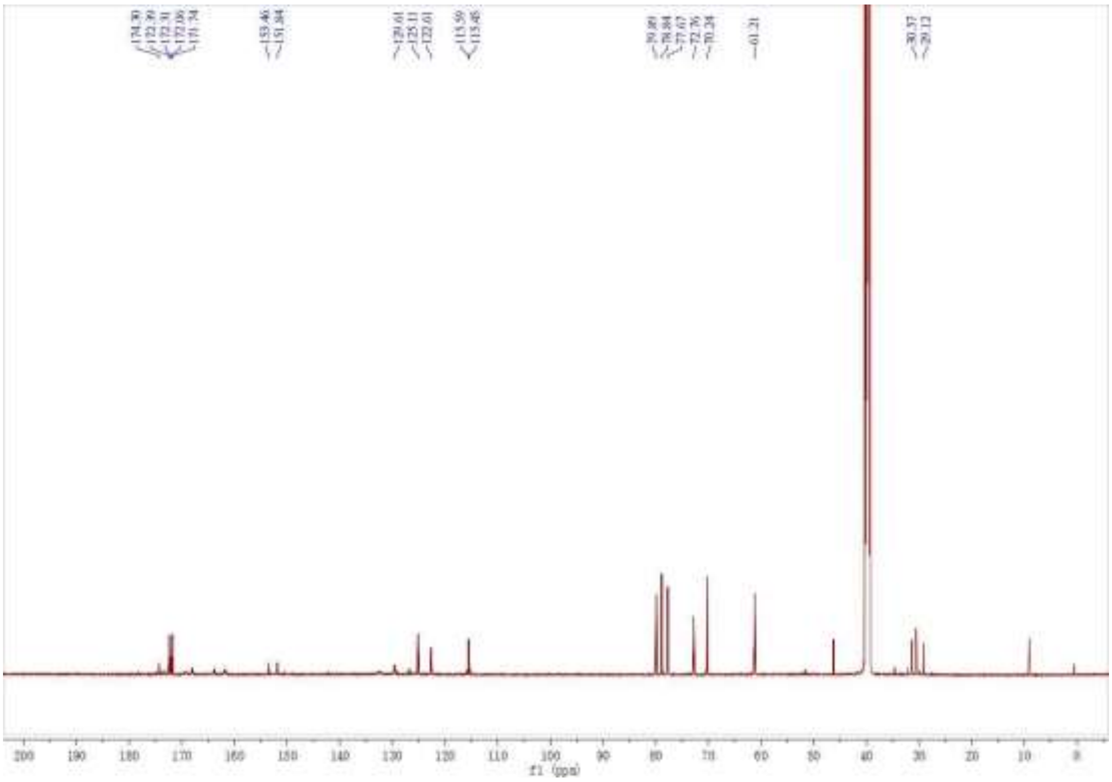

Figure S44. The ^{13}C-NMR of compound 14k.

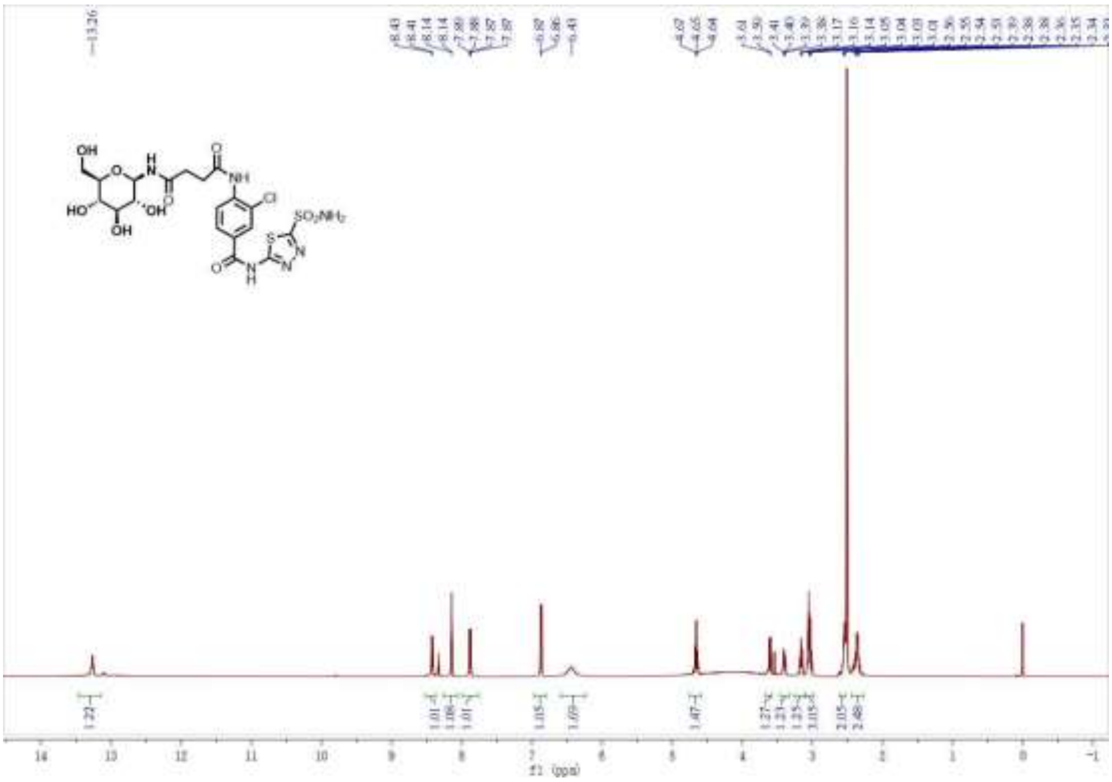

Figure S45. The ^{1}H-NMR of compound 14l.

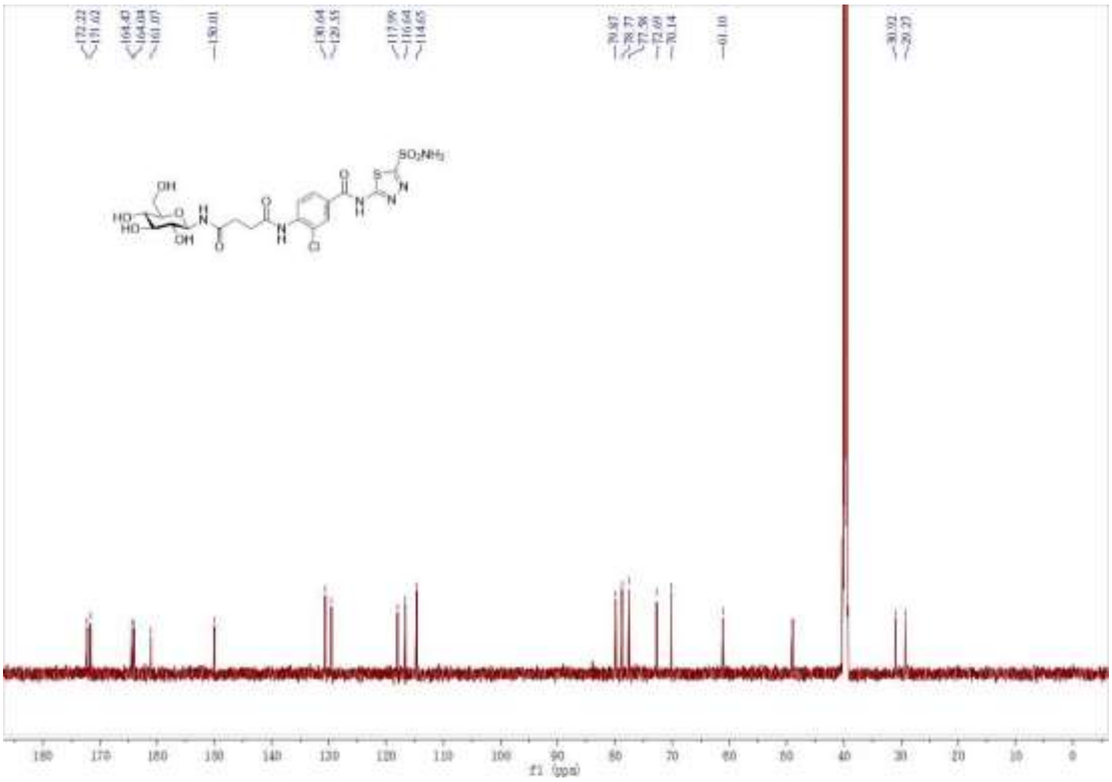

Figure S46. The ^{13}C-NMR of compound 14l.

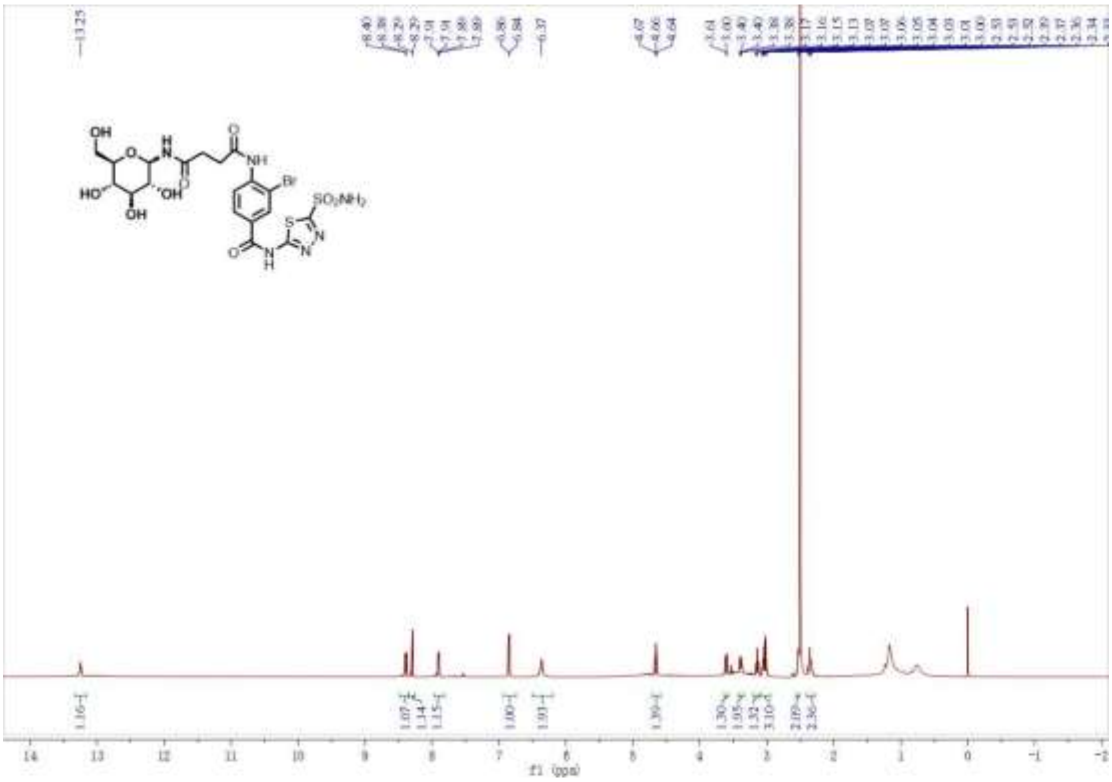

Figure S47. The ^{1}H-NMR of compound 14m.

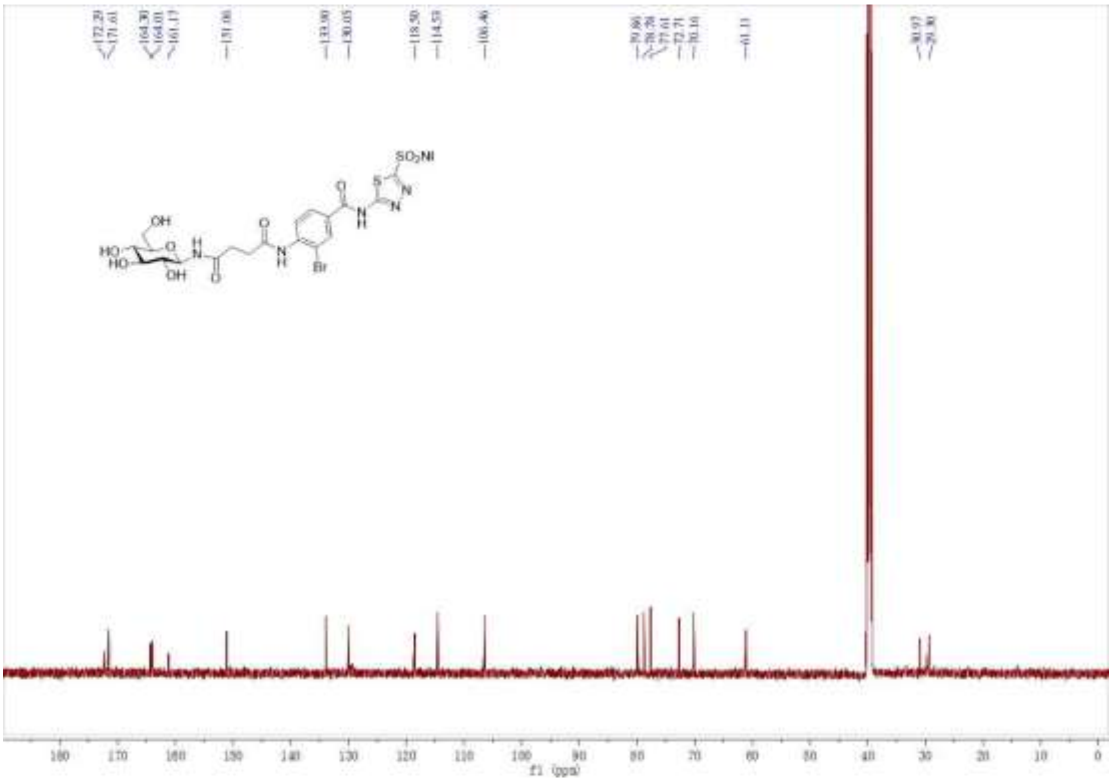

Figure S48. The ^{13}C-NMR of compound 14m.

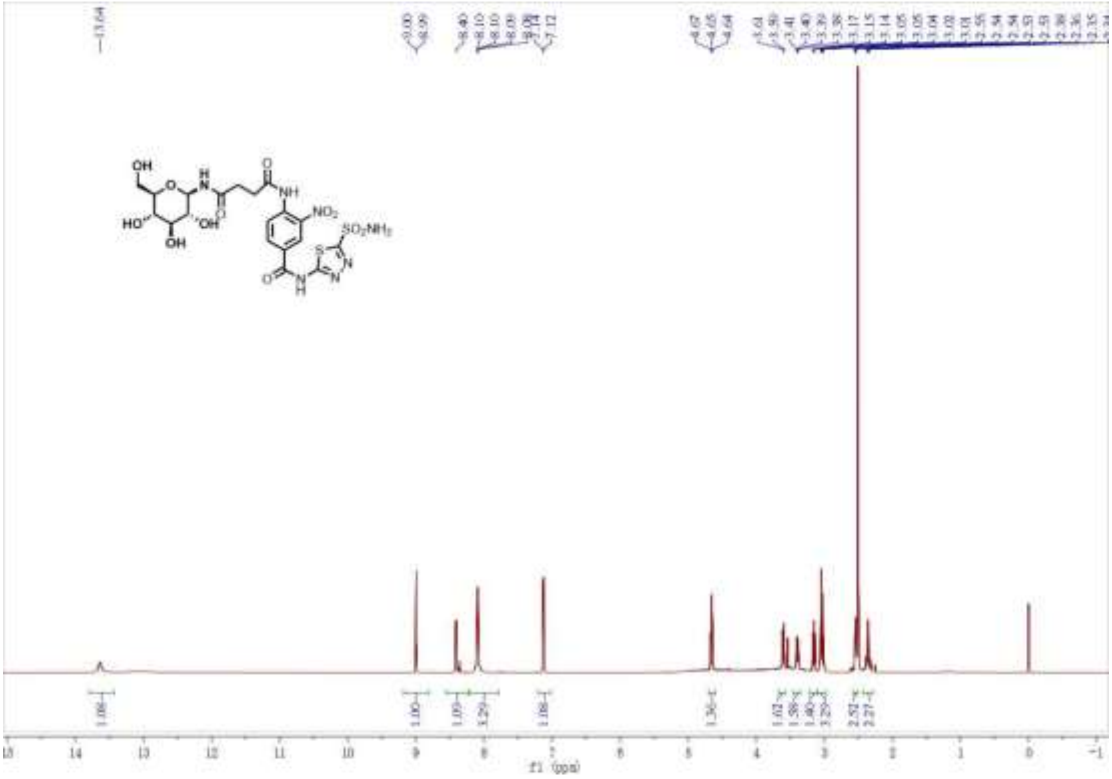

Figure S49. The ^{1}H-NMR of compound 14n.

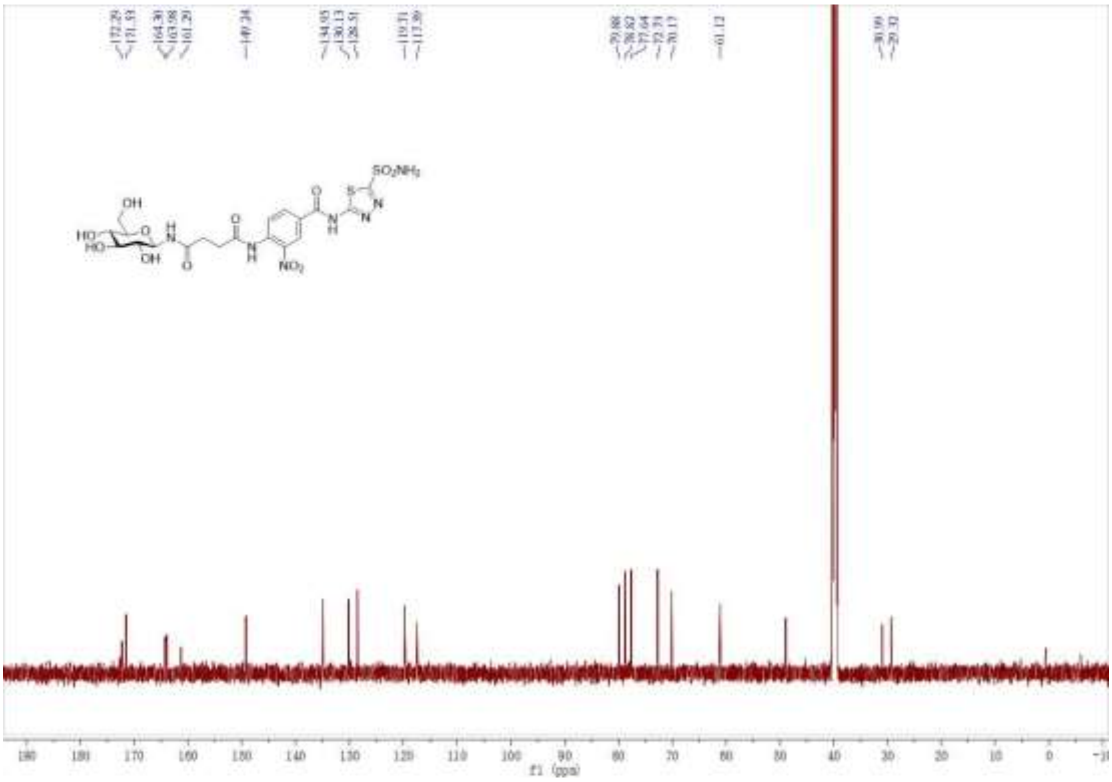

Figure S50. The ^{13}C-NMR of compound 14n.

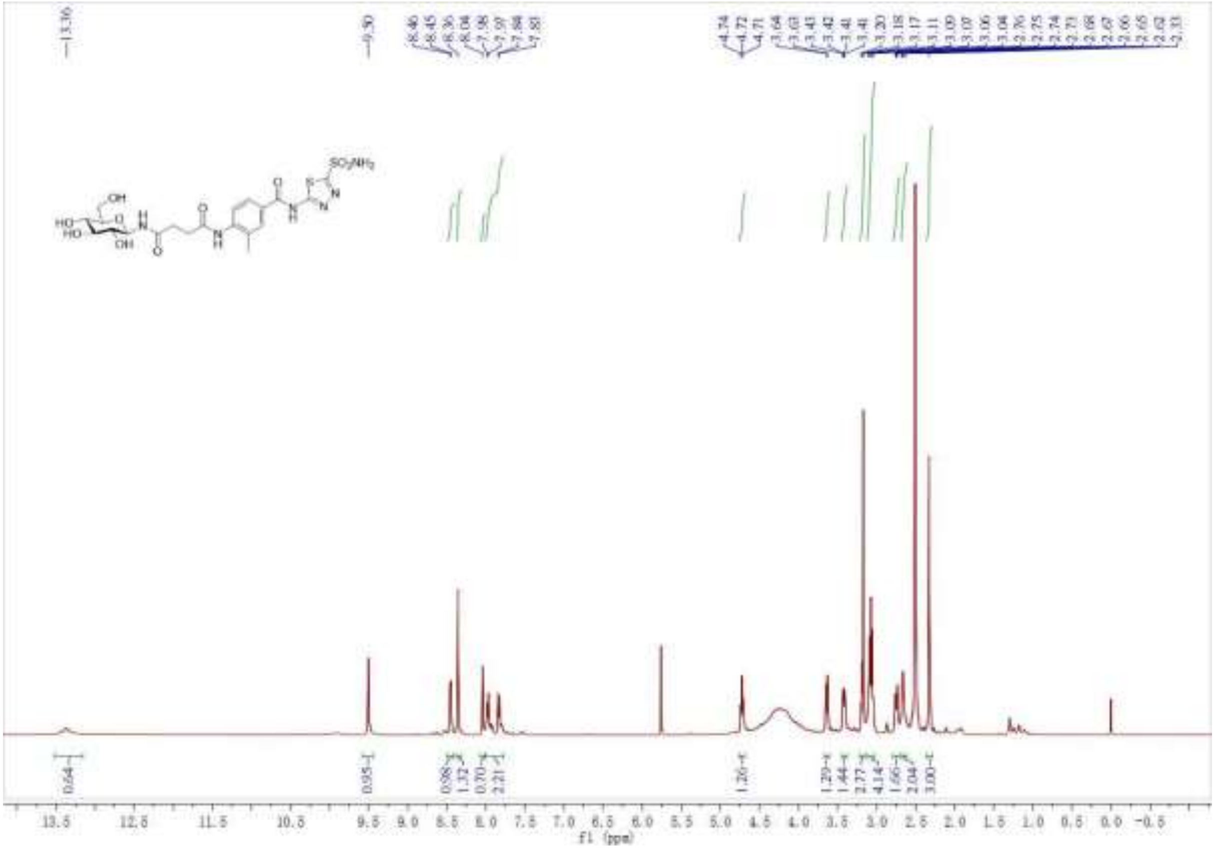

Figure S51. The ^{1}H-NMR of compound 14o.

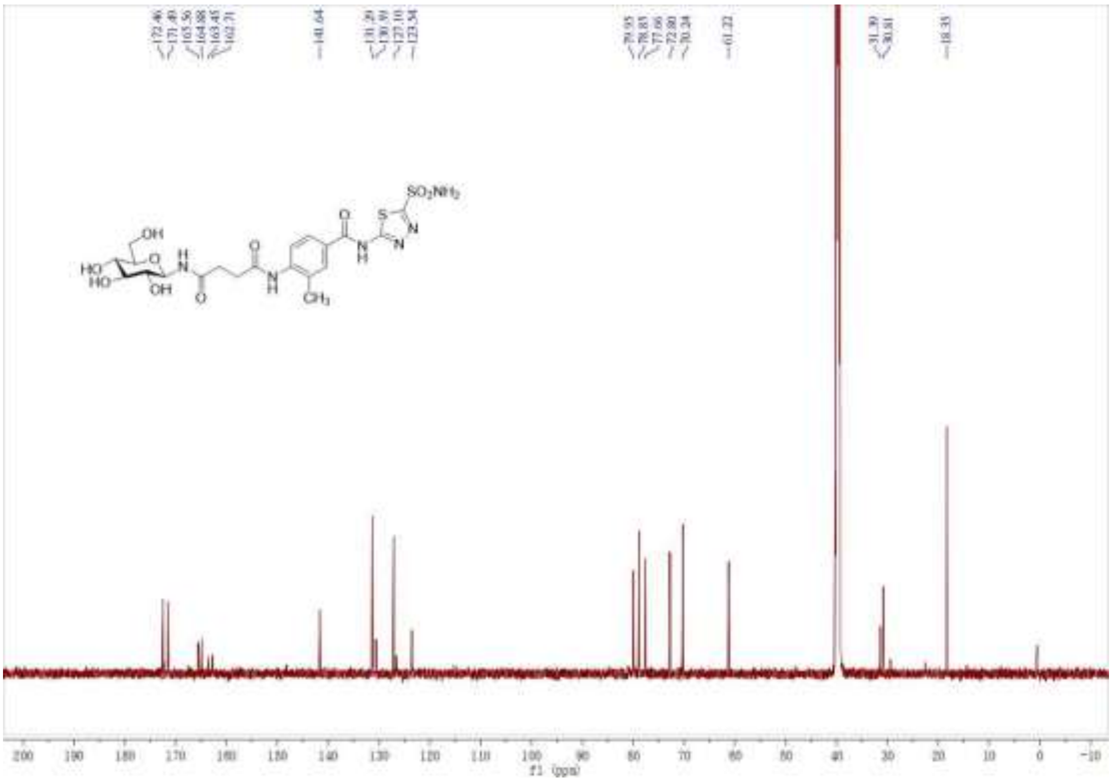

Figure S52. The ^{13}C-NMR of compound 14o.

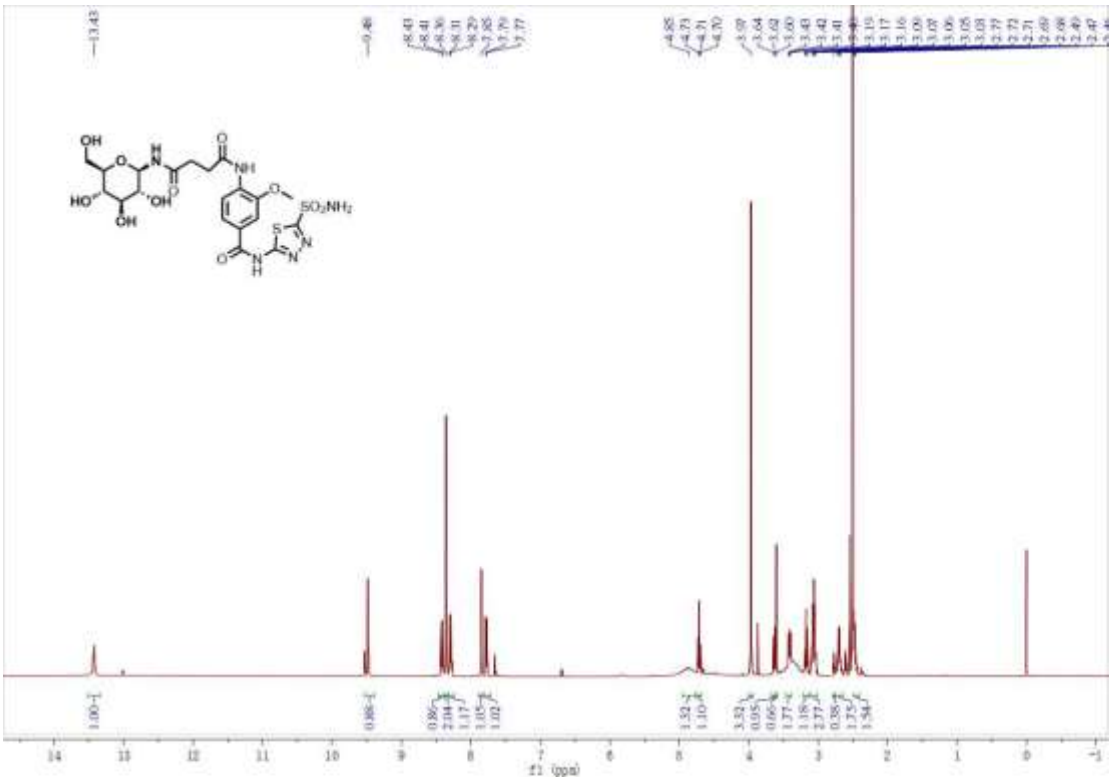

Figure S53. The ^{1}H-NMR of compound 14p.

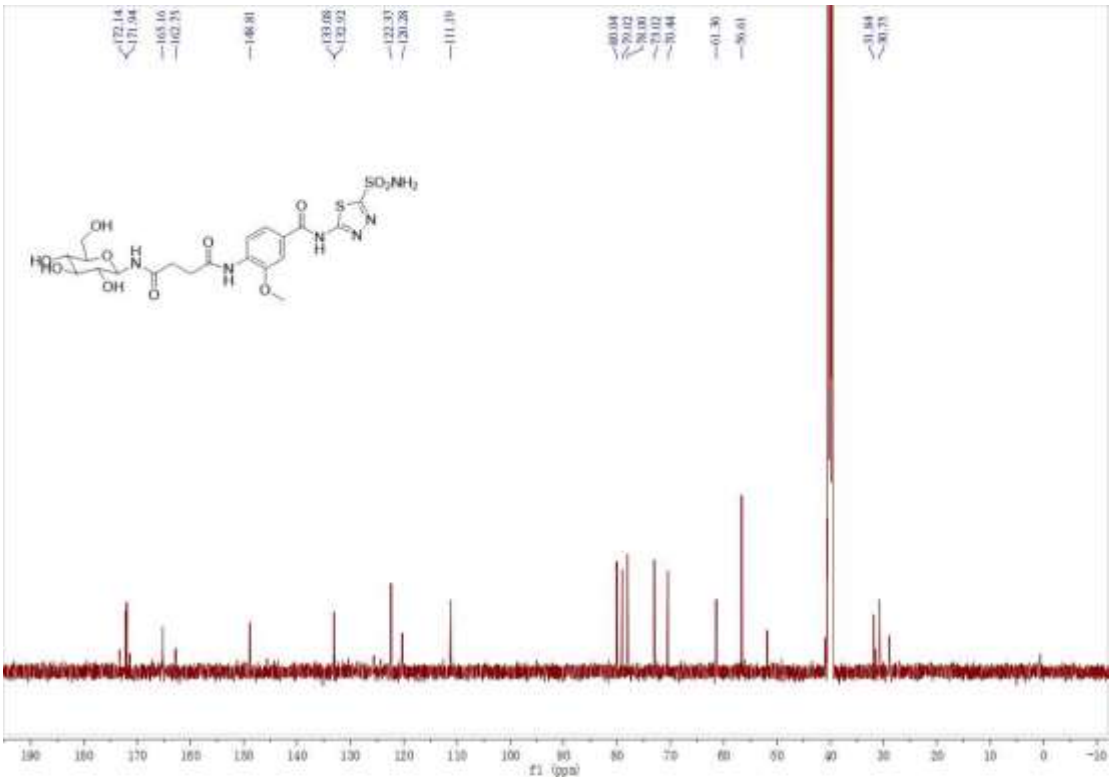

Figure S54. The ^{13}C-NMR of compound 14p.

2D-NMR Spectra

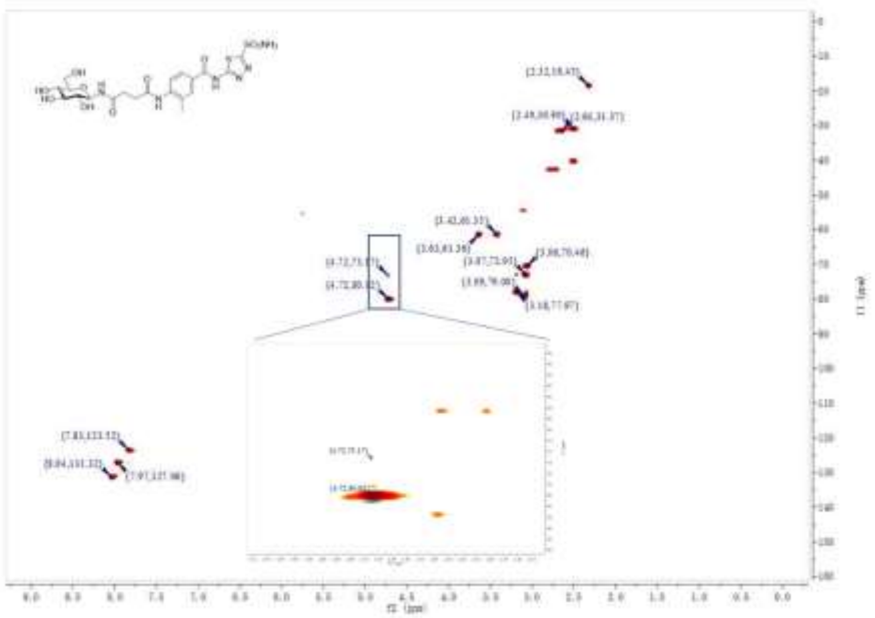

Figure S55. The HSQC spectra of compound 14o.

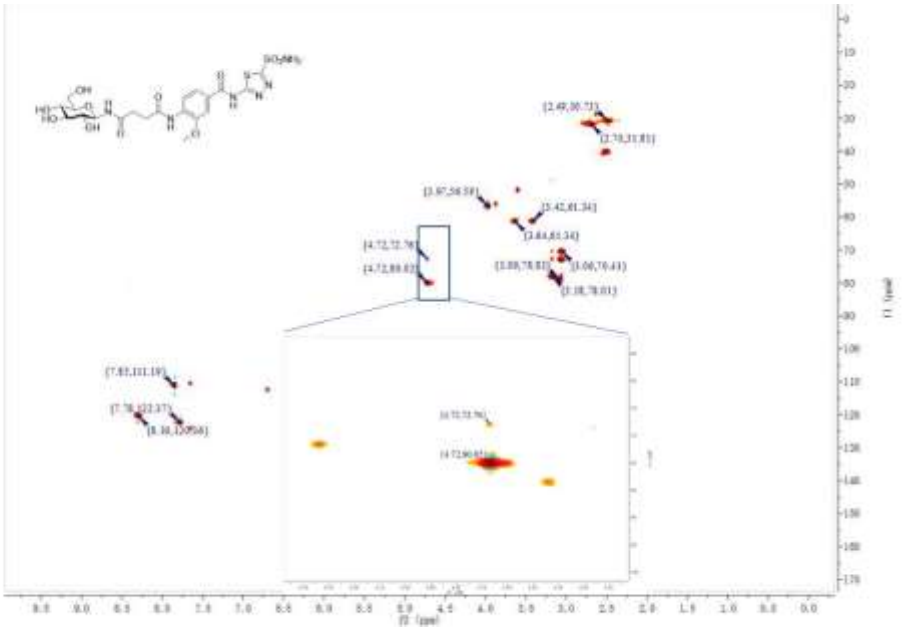

Figure S56. The HSQC spectra of compound 14p.

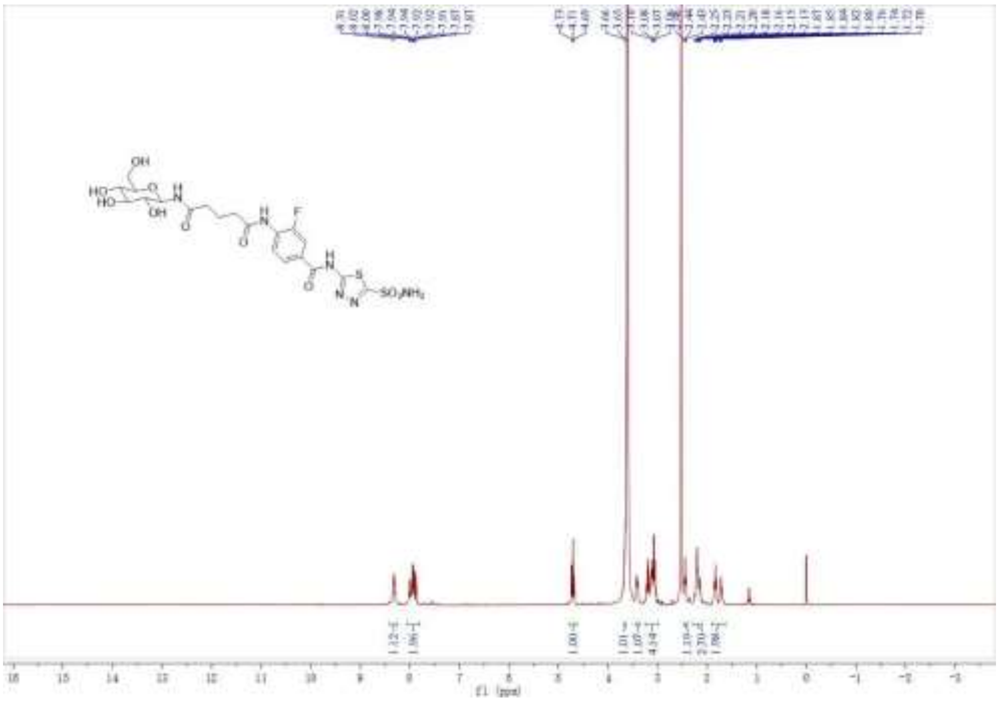

Figure S57. The ^{1}H-NMR of compound 14c with D_2O-exchange.

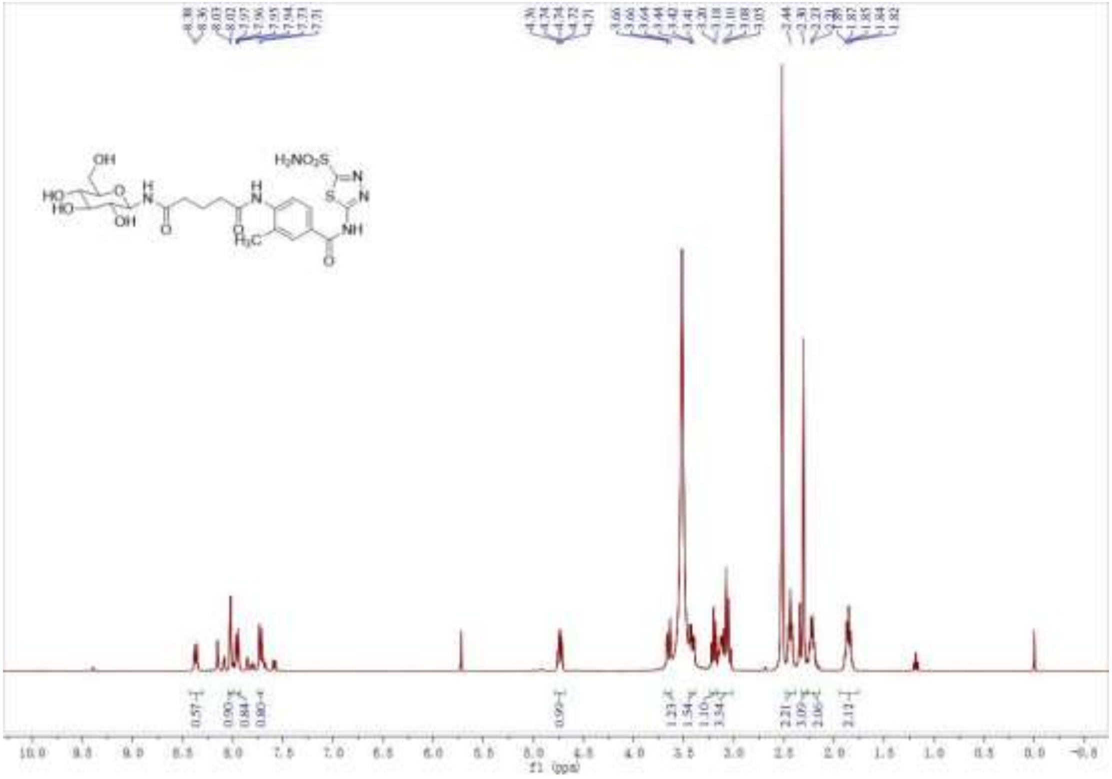

Figure S58. The ^{1}H-NMR of compound 14g with D_2O-exchange.

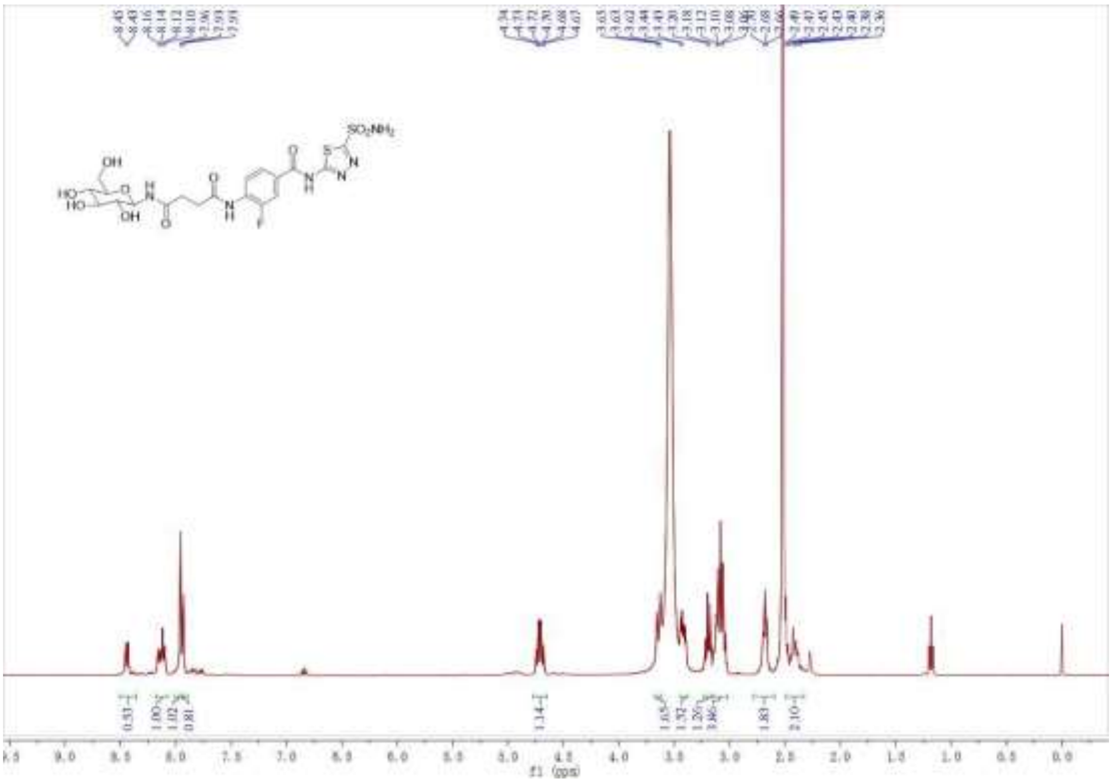

Figure S59. The ^{1}H-NMR of compound 14k with D_2O-exchange.

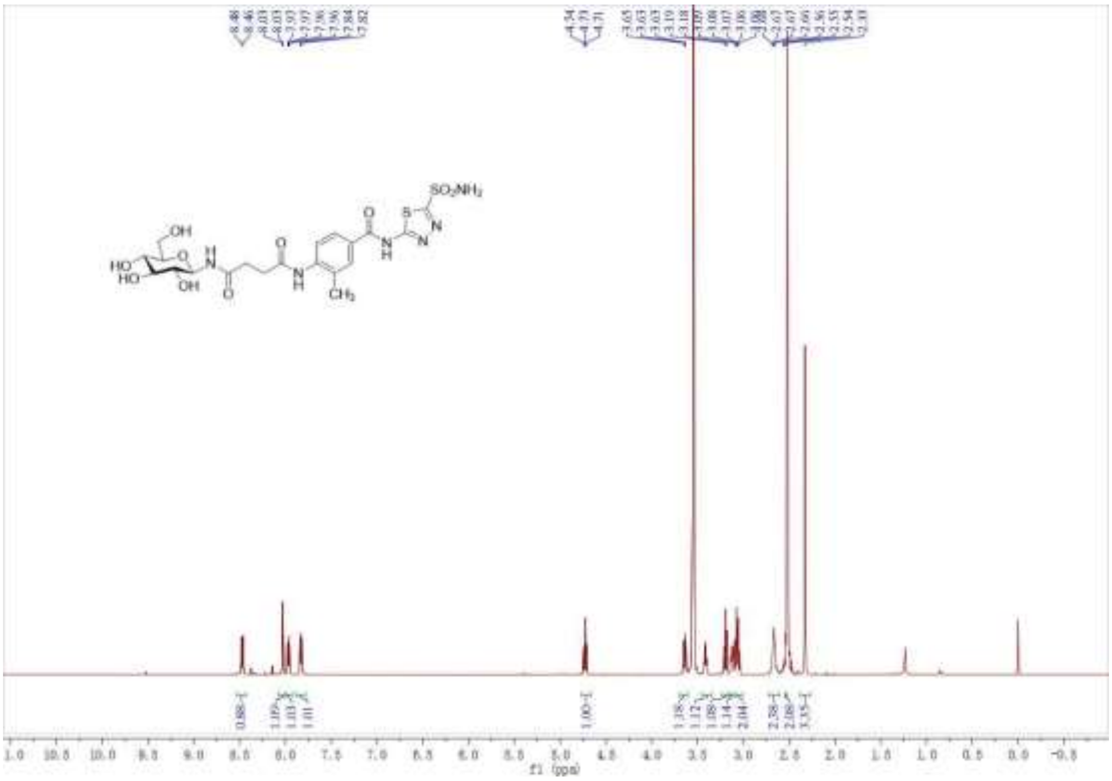

Figure S60. The ^{1}H-NMR of compound 14o with D_2O-exchange.

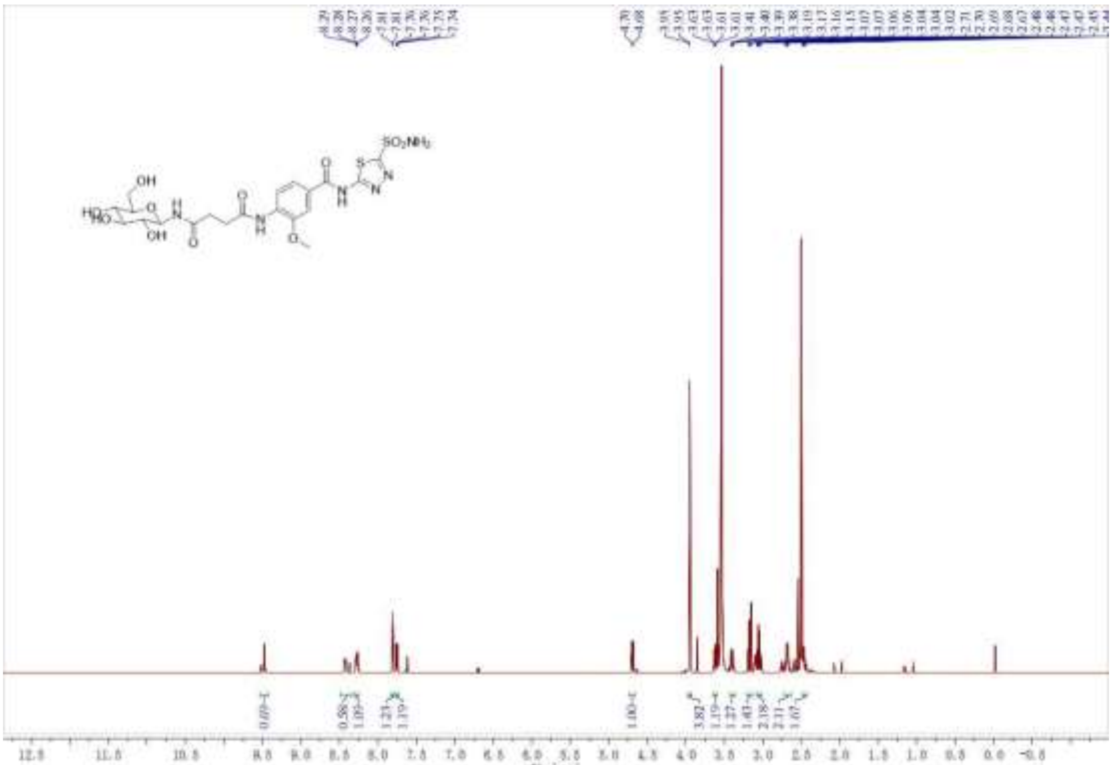

Figure S61. The ^{1}H-NMR of compound 14p with D_2O-exchange.

Modes of binding figures

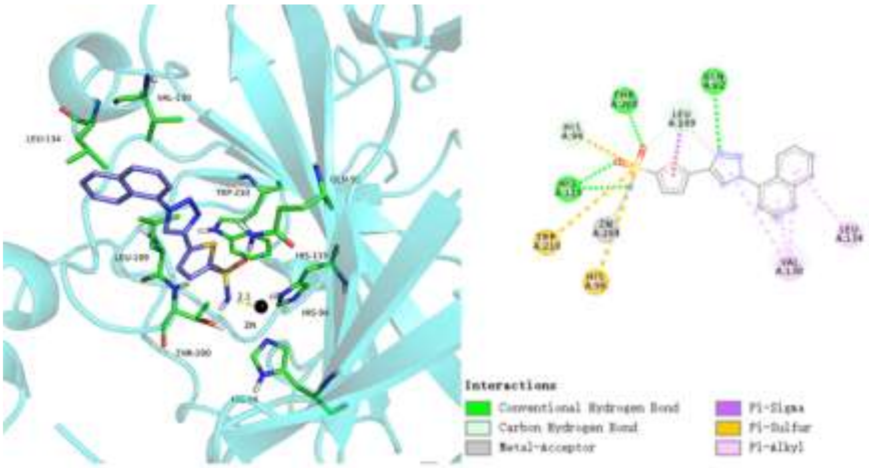

Figure S62. The modes of binding for co-crystal structure 5FL4 in CA IX.

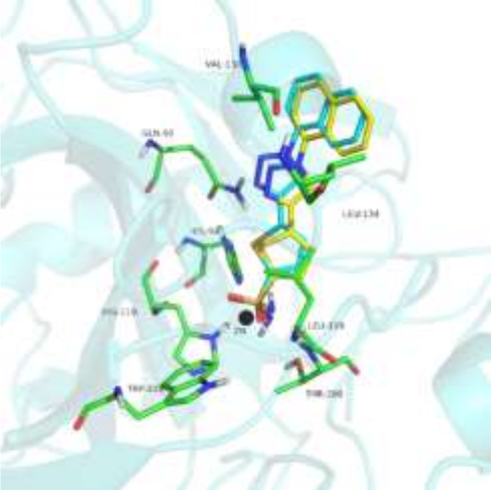

Figure S63. Co-crystalized pose (yellow) and the re-docked pose (blue) of 5FL4.

Supplementary data

Table S1. Binding energy, hydrogen bonding interacting residues, H-binding distance, hydrophobic interacting residues of 14a, 14e and 14i.

Compound	binding energy	hydrogen bonding interacting residues	H-binding distance (Å)	hydrophobic interacting residues
14a	-4.543	Gln71	2.28856	His68
		Gln92	3.08217	His94
		His119	2.75462	His96
		His119	2.55636	Leu199
		Thr200	1.78295	
14e	-3.130	Gln71	1.80873	His94
		His119	2.67012	His94
		Thr200	1.82586	Leu199
		Thr201	2.74211	
		Thr201	3.02351	
14i	-4.508	Gln71	1.8855	His94
		Gln92	2.25552	His96
		Gln92	2.62697	Val121
		Thr200	1.8108	Leu199
		Thr201	2.76773	
		Asp131	2.03706	
		Asp131	2.04039	

Table S2. The IC_{50} of Taxol

Compound	HT-29	MDA-MB-231
Taxol	111 ng/mL	99 ng/mL

Table S3. The calculated ADMET information of some compounds

Compounds	ADMET Solubility	ADMET Solubility Level	ADMET BBB Level	ADMET Absorption Level	ADMET clogP
14a	-4.668	2	4	3	-2.069
14b	-4.784	2	4	3	-2.069
14c	-5.6	2	4	3	-1.864
14g	-5.079	2	4	3	-1.583
14i	-5.23	2	4	3	-2.086
14j	-4.565	2	4	3	-2.526
14h	-4.675	2	4	3	-2.526
14p	-5.146	2	4	3	-2.542
